# Anti-Markovnikov Intermolecular Hydroamination of Alkenes and Alkynes: A Mechanistic View

**DOI:** 10.1021/acs.chemrev.2c00482

**Published:** 2023-07-05

**Authors:** Jorge Escorihuela, Agustí Lledós, Gregori Ujaque

**Affiliations:** † Departament de Química Orgànica, Universitat de València, 46100 Burjassot, Valencia, Spain; ‡ Departament de Química and Centro de Innovación en Química Avanzada (ORFEO-CINQA), Universitat Autònoma de Barcelona, 08193 Cerdanyola del Vallès, Barcelona, Catalonia, Spain

## Abstract

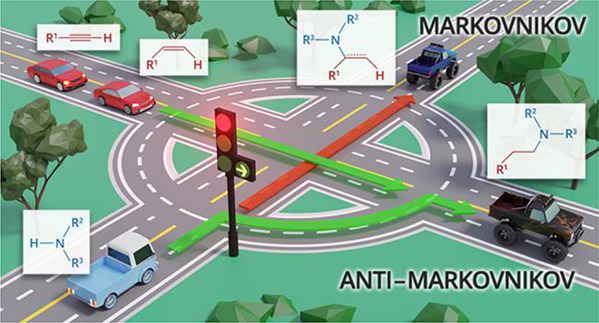

Hydroamination, the addition of an N–H bond across a C–C multiple bond, is a reaction with a great synthetic potential. Important advances have been made in the last decades concerning catalysis of these reactions. However, controlling the regioselectivity in the amine addition toward the formation of anti-Markovnikov products (addition to the less substituted carbon) still remains a challenge, particularly in intermolecular hydroaminations of alkenes and alkynes. The goal of this review is to collect the systems in which intermolecular hydroamination of terminal alkynes and alkenes with anti-Markovnikov regioselectivity has been achieved. The focus will be placed on the mechanistic aspects of such reactions, to discern the step at which regioselectivity is decided and to unravel the factors that favor the anti-Markovnikov regioselectivity. In addition to the processes entailing direct addition of the amine to the C–C multiple bond, alternative pathways, involving several reactions to accomplish anti-Markovnikov regioselectivity (formal hydroamination processes), will also be discussed in this review. The catalysts gathered embrace most of the metal groups of the Periodic Table. Finally, a section discussing radical-mediated and metal-free approaches, as well as heterogeneous catalyzed processes, is also included.

## 1 Introduction

**Scheme 1:**
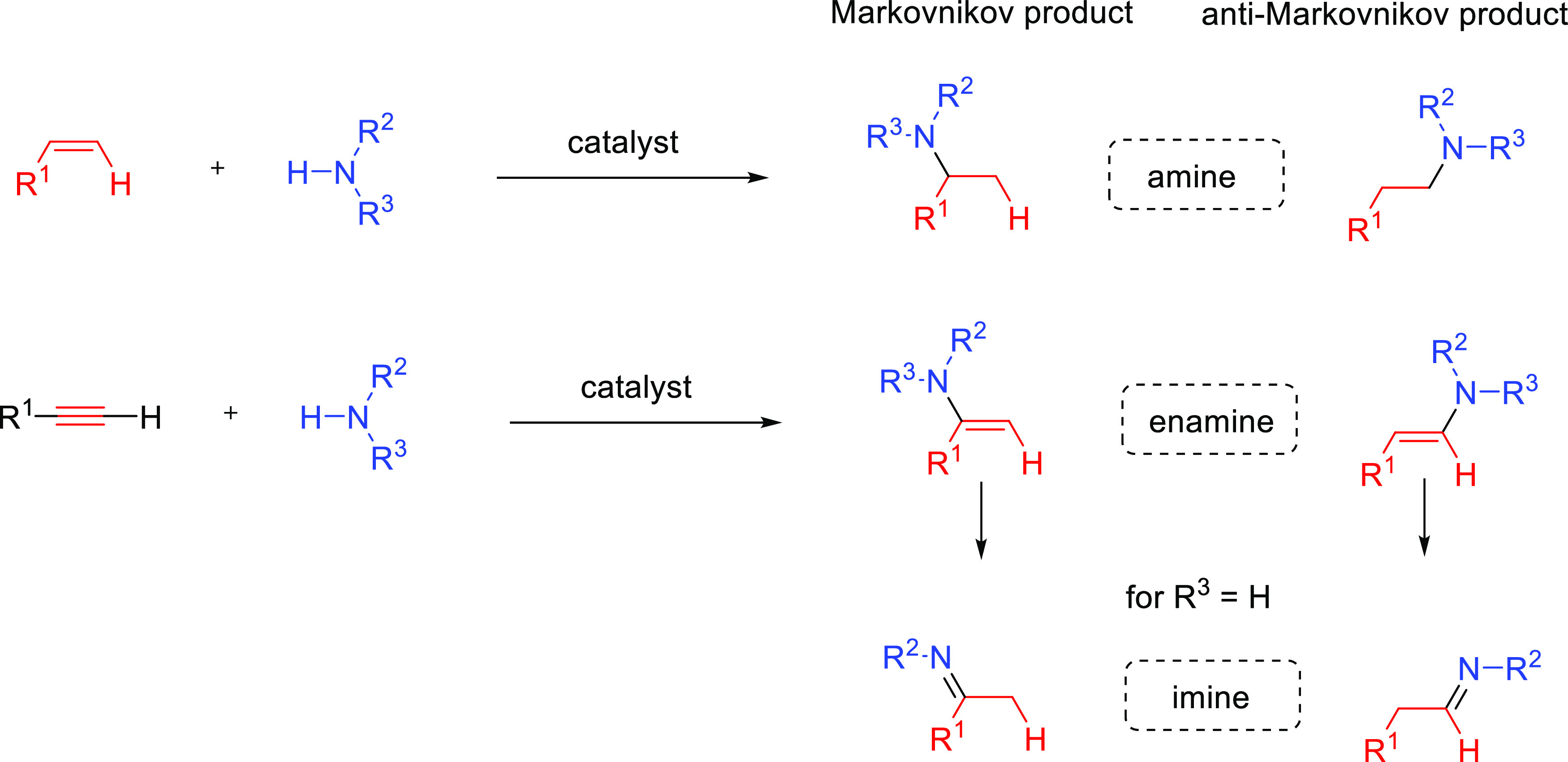
Possible Products Resulting from the Intermolecular Catalytic Hydroamination of Terminal Alkenes and Alkynes

Hydroamination can be described as the net addition of an N–H unit to a C–C unsaturated bond involving the formation of a C–H and a C–N bond and the cleavage of the N–H bond.^1−3^ The reaction formally consists of the addition of an amine (primary or secondary) to a multiple C–C bond, either in an inter- or intramolecular version.^4−6^ This reaction is very useful to generate amines from readily available reactants such as alkenes (including vinyl arenes, conjugated dienes, allenes, or ring-strained alkenes) and alkynes with high atom economy. When looking at hydroamination of alkenes, the reaction affords secondary and tertiary amines as products, whereas hydroamination of alkynes generates *N-*containing species (enamines and imines) that are valuable themselves as synthetic intermediates or can be further transformed in other valuable products (Scheme 1).^7,8^ Both reactions are extremely important for the preparation of nitrogen-containing organic compounds with applications in organic synthesis as well as in the pharmaceutical chemistry.^9−11^

Hydroamination is commonly feasible from a thermodynamic point of view, although it depends on the particular combination of reactants.^2,12^ Generally, hydroamination of alkenes is thermoneutral or favorable (as experimentally measured for hydroamination of vinylarenes with arylamines),^13^ whereas the corresponding hydroamination of alkynes is calculated to be more favorable.^14,15^ For instance, the reaction energies for the addition of ammonia to ethene and ethyne are calculated to be – 9.8 and – 17 kcal/mol, respectively.^16,14^

Nevertheless, the direct coupling between an amine and the unsaturated C–C substrate is not kinetically favorable because both are considered to be electron-rich reactants. Therefore, hydroamination of unsaturated C–C bonds requires the use of catalysts.^17^ Consequently, the quest for and development of efficient and selective hydroamination catalysts is a major goal for the synthetic community.^18^ Among hydroamination of multiple C–C bonds, hydroamination of alkenes is generally more difficult than that of alkynes given the lower reactivity and electron density of the former.^19^ In the same vein, intermolecular hydroaminations are more difficult than the intramolecular versions, mainly attributed to the entropic penalty. Considering all these facts, intermolecular hydroaminations of unactivated alkenes are the most challenging transformations.^20^ Moreover, for alkenes, note that achieving anti-Markovnikov selectivity is a completely different objective when dealing with vinylarenes, 1,3-dienes, or other activated olefins. These substrates are electronically biased toward anti-Markovnikov additions, and the outcome used to be mainly substrate-controlled.^21^

Chemical processes which allow the obtention of hydroaminated products in a single step offer significant environmental and economic advantages according to the principles of green chemistry (i.e., atom economy, reduce derivatives, catalysis).^22^ Great advances were accomplished in this area, and the direct addition of amines to C–C multiple bonds has been achieved for many catalytic processes. A key issue in the addition of *N-*nucleophiles over terminal alkenes and alkynes is the control of the regioselectivity.^23^ The addition may give rise to the Markovnikov (amine adding to the more substituted carbon) or anti-Markovnikov (amine adding to the less substituted carbon) products (Scheme 1). Generally, Markovnikov addition is favored; thus, obtaining a reversal in regioselectivity, i.e. anti-Markovnikov addition, is a challenge in modern organic chemistry.^21,24^ In fact, anti-Markovnikov hydroamination of alkenes was named in 1993 as one of the 10 greatest challenges for catalysis research,^20^ and despite the progress accomplished over the last three decades, it still remains as a highly challenging reaction for unactivated alkenes and alkynes. Dominating this process would pave the way for obtaining linear alkyl amines (and enamines) from cheap and abundant feedstocks.^7^

The present review focuses on intermolecular anti-Markovnikov hydroaminations of terminal alkenes and alkynes. The aim is to describe the systems in which intermolecular hydroamination of terminal alkynes and alkenes with anti-Markovnikov regioselectivity has been achieved. The focus will be placed on the mechanistic aspects of such reactions and to elucidate/expound the factors that favor the anti-Markovnikov regioselectivity. The papers collected herein include mechanistic proposals supporting the formation of the anti-Markovnikov product. In addition to outlining the experimentally characterized systems, the proposed factors governing the regioselectivity are analyzed for each kind of systems.

Although anti-Markovnikov regioselective processes entailing direct addition of the amine to the C–C multiple bond have been developed, the difficulties in directing the amine addition toward the terminal carbon have led researchers to devise alternative pathways, avoiding direct attack of the amine on the C–C multiple bond, to achieve anti-Markovnikov regioselectivity.^21^ We have named these reactions formal hydroamination processes, and these will also be discussed in this review. Despite their importance in synthetic organic chemistry, hydroaminations of terminal allenes are not considered here.^25^ Furthermore, addition of amides, imides, or most of the other type of *N*-containing compounds, as well as the oxidative amination processes, are not included or are only occasionally mentioned. The related hydroaminoalkylation reaction has been very recently reviewed.^26^ Comprehensive reviews on hydroaminations can be found in the recent literature. In this regard, Müller, Hultzsch, and co-workers have published an exhaustive review dealing with all variants of hydroamination.^2^ Hydroamination of alkynes has been reviewed by Doye^15,17,27^ and more recently by Mahdavi and coauthors.^28^ Hydroamination of alkenes has been reviewed by Reznichenko and Hultzsch^29^ and by Patton and Cremeens,^30^ with the latter focused on the intramolecular version. Recent hydroamination catalyst developments from group 3–5 elements have been summarized by Hannedouche and Schulz.^31^ Control of the regiochemistry of diverse additions of nucleophilic reagents to unsaturated compounds, including hydroamination, was reviewed by Beller et al.^23^ General mechanistic aspects have been discussed for early transition metal and main group metal^32^ and late transition metal-catalyzed hydroaminations.^33^

The diversity of catalysts developed for anti-Markovnikov hydroamination reactions involve numerous metals across the whole Periodic Table. Thus, there are catalysts based on alkali and alkaline earth metals, early transition metals, and late transition metals, as well as lanthanides and actinides. Regarding the regioselectivity, lanthanides,^34^ actinides,^35^ and early transition metals^21,32,36^ were developed earlier than late transition metals to afford anti-Markovnikov products.^1,3,36,37^ Nevertheless, there are many examples of anti-Markovnikov addition for late transition metals, as will be shown in the upcoming sections. The catalytic systems for anti-Markovnikov hydroaminations are gathered in this review according to the group of the Periodic Table to which they belong: alkali, alkaline earth, lanthanides, actinides, and early and late transition metals. When describing each group of these of catalysts, there is one subsection devoted to relating the catalysts and their reactivity and another focusing on the factors responsible for the regioselectivity according to the proposed reaction mechanism. To discern the step at which regioselectivity is decided, a key point is the reaction mechanism that takes place for every catalyst. Thus, before describing the details of the reported catalysts, the following section gives a general overview on the reaction mechanisms proposed so far for the hydroamination process.

## 2 General Overview of Hydroamination Mechanisms

**Figure 1:**
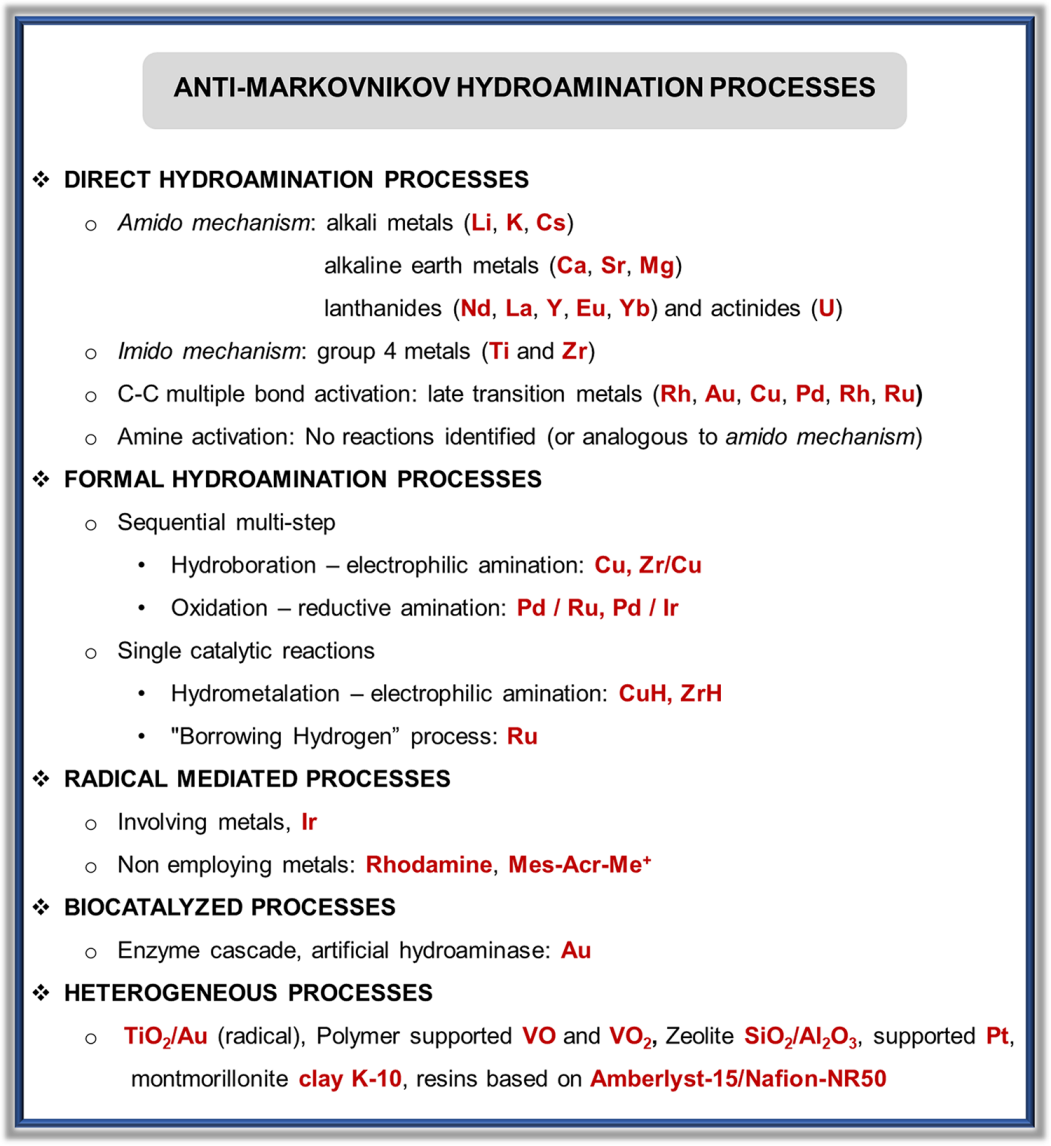
Summary of proposed processes for the intermolecular anti-Markovnikov hydroamination of alkenes and alkynes.

This section describes the general and accepted mechanisms for the anti-Markovnikov addition of amines to alkenes and alkynes. The mechanistic pathways are divided in two different subsections, titled as Direct Hydroamination Mechanisms and Mechanism of Formal Hydroamination Reactions. The first one describes those processes involving a direct addition of an amine over a C–C bond of the unsaturated substrate. The second subsection corresponds to other strategies that involve the combination of processes to obtain the formal anti-Markovnikov addition of amines to unsaturated substrates. Figure 1 collects all the reaction mechanisms proposed for anti-Markovnikov addition of amines to alkenes and alkynes, as well as the metal elements involved as catalysts for each of these mechanisms.

### 2.1 Direct Hydroamination Mechanisms

The mechanisms presented here involve the direct hydroamination of the unsaturated substrate. The regioselectivity of the hydroamination product is decided at the step at which the carbon–nitrogen bond is being formed. The way the carbon–nitrogen bond is formed depends on the nature of the catalyst, which can activate either one of the two reactants, the amine or the unsaturated C–C compound. Four general mechanisms have been described for intermolecular hydroaminations.^2,32,33^ On one hand, main group metals from groups 1 and 2, early transition metals from groups 3–5, and lanthanides start by generating a metal–N bond and operate via activation of the amine to form catalytically active metal–amido or metal–imido species.^32^ On the other hand, late transition metal catalysts can activate the C–C multiple bond by π coordination or the amine by forming an amido complex.^33^ Note that early transition metals are less suitable to activate alkenes because of their lack of d-electrons. In this section we will summarize the main features of each of these mechanisms, focusing on the C–N bond forming step.

#### 2.1.1 Amido Mechanism—The “Lanthanide-like” Mechanism

**Scheme 2:**
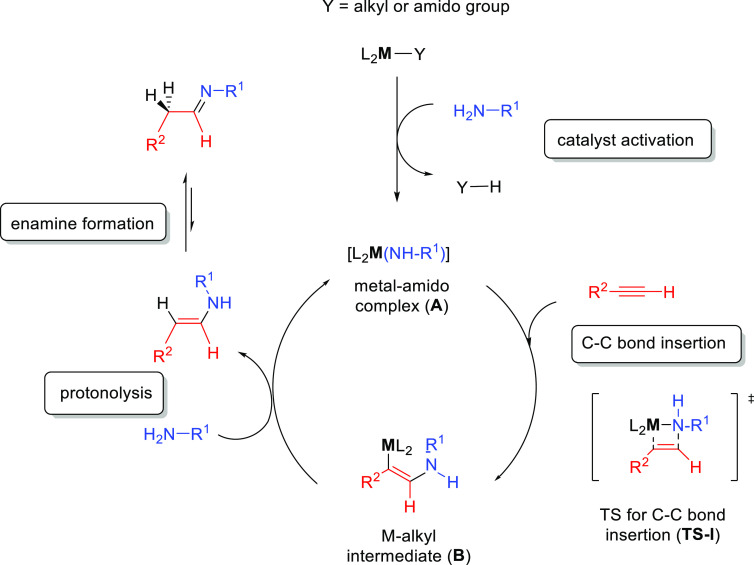
Schematic Representation of the “Amido Mechanism” for Anti-Markovnikov Hydroamination of Terminal Alkynes

Main group metal and rare earth metal catalysts proceed with similar reaction mechanisms involving the insertion of the unsaturated C–C bond into the metal–amide bond, which is generally referred to as the “lanthanide-like” mechanism.^32^ The hydroamination mechanism for a terminal alkyne is shown in Scheme 2.

The catalytically active species in this mechanism is a metal–amido complex (**A**) formed by rapid protonolysis of the initial ligand by the amine substrate. In the next step the unsaturated C–C bond inserts into the metal–amido bond through a four-membered transition state (**TS-I**). Finally, protonolysis of the M–alkyl species (**B**) occurs through the entrance of a new amine molecule which delivers the enamine product and regenerates the catalytically active metal–amido species (**A**).^34,38,39^ Depending on the formed compound, the enamine can tautomerize to an imine product. A similar mechanism can be envisaged for alkenes, yielding the amine as the addition product. In this stepwise insertion/protonolysis mechanism it is believed that the insertion step is the rate-determining step (RDS) of the process, followed by a rapid protonolysis step.^40,41^ The C–N bond formation, where the regioselectivity is decided, takes place in the C–C insertion step.

#### 2.1.2 Imido Mechanism

The imido mechanism has been accepted for the hydroamination of alkynes and allenes catalyzed by group 4 metals. As shown inScheme 3, the catalytically active species involves a metal imido intermediate and the catalytic cycle has three main steps. In the first one, the catalytically active species, which is assumed to be a metal imido complex (**C**), undergoes a reversible [2 + 2] cycloaddition with an alkyne to generate an azametallacyclobutene intermediate (**D**). Note that the direct [2 + 2] cycloaddition of an imine to the alkyne (alkene) is forbidden by orbital symmetry.

**Scheme 3:**
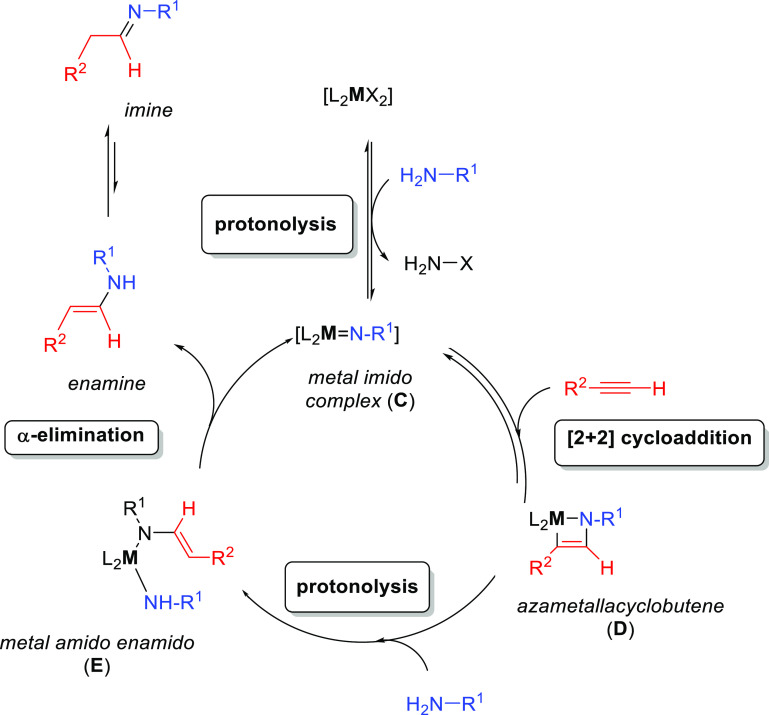
Schematic Representation of the “Imido Mechanism” for the Anti-Markovnikvov Hydroamination of Internal Alkynes.

In the next step, coordination and protonolysis of the amine to the azametallacyclobutene (**D**) intermediate yields a metal amido enamido species (**E**). This has been observed as the rate-determining step.^42^ Finally, in the last step, the enamine/imine product is released through an α-elimination step and the metal imido complex is regenerated. This mechanism has been strongly supported by the isolation and characterization of the 4-membered ring azametallacyclobutene species.^43,44^ In this mechanism, the [2 + 2] cycloaddition step determines the regioselectivity of the addition.

#### 2.1.3 C–C Multiple Bond Activation Mechanism

Several mechanisms involving the C–C multiple bond activation have been described: (i) nucleophilic attack of the amine on the coordinated unsaturated substrate, (ii) coordination of the unsaturated substrate and subsequent conversion to a metal–vinylidene intermediate (a mechanism only feasible for alkynes), and (iii) nucleophilic attack of the amine to the substrate that is coordinated through an arene substituent.

**Scheme 4:**
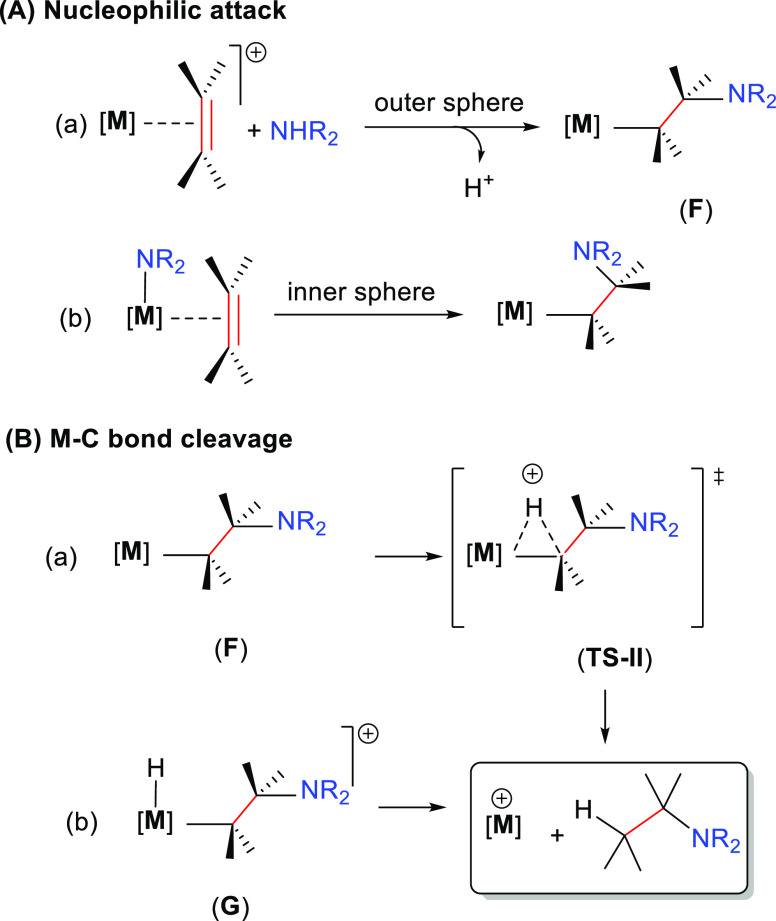
General Mechanism for the Nucleophilic Addition of the Amine on the Unsaturated Substrate Coordinated to the Metal: (A) Nucleophilic Attack; (B) M–C Bond Cleavage

In the first mechanism the nucleophilic attack of the amine takes place to the metal-coordinated C–C multiple bond (Scheme 4). The approach of the amine from outside of the metal complex is described as an outer-sphere pathway. If both reactants are coordinated to the metal center prior to the addition of the amine to the unsaturated substrate, it is described as an inner-sphere mechanism. In both pathways the regioselectivity is defined at the C–N bond forming step (Scheme 4A).^33^

In the subsequent step, the reaction mechanism is terminated by the protonolysis of the M–C bond (**F**) giving rise to the hydroaminated product. Two alternative pathways have been described for this process: (a) a concerted protonation of the ligand through **TS-II** (thus avoiding the formal oxidation of the metal center), often called as direct protonolysis, and (b) a stepwise protonation of the metal center to generate a metal–hydride species (**G**), formally increasing the oxidation state by +2, finally followed by a reductive elimination (Scheme 4B).^33^ Nevertheless, this process is relatively difficult to anticipate for late transition metal intermediates because they have a large tendency to undertake β-hydride elimination (producing the oxidative amination product instead of the hydroamination product).^45^ Thus, to promote hydroamination, the undesired β-hydride elimination should be suppressed (although sometimes both products are obtained).^16^

**Scheme 5:**
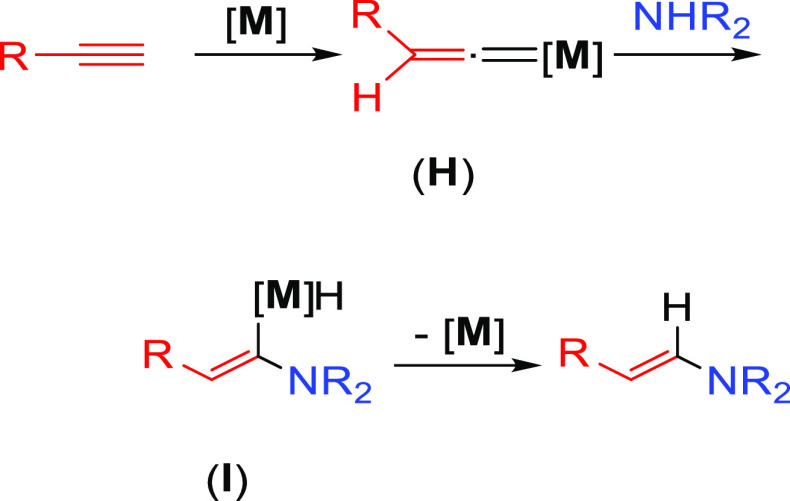
General Mechanism for C–C Multiple Bond Activation Showing the Formation of a Metal–Vinylidene Intermediate Previous to the Nucleophilic Addition of the Amine

An alternative mechanism for the activation of alkynes based on a metal vinylidene intermediate has also been proposed (Scheme 5).^46^ In this case, the terminal alkyne, initially coordinated to the metal center, is subsequently converted into a metal–vinylidene intermediate (**H**). Afterward, the amine undertakes a nucleophilic addition on the α carbon; the regioselectivity of the process is defined at this step. The generated intermediate (**I**) contains a hydride and an α-aminovinyl group coordinated to the metal center. A subsequent reductive elimination gives rise to the anti-Markovnikov hydroaminated product.

**Scheme 6:**
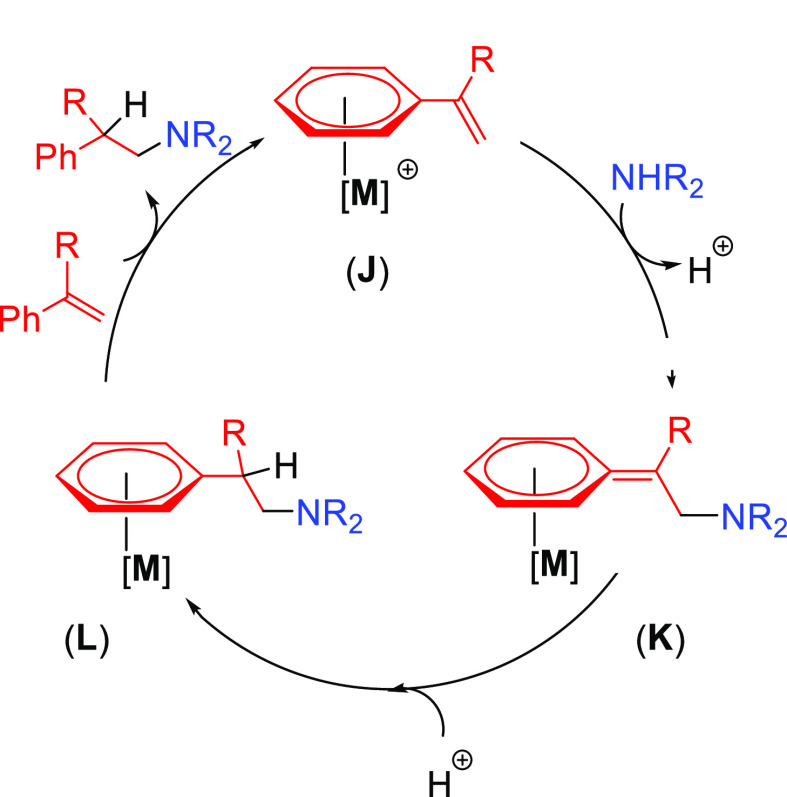
Main Reaction Steps for Hydroamination of Vinylarenes/Vinylpyridines Involving Coordination to the Metal Center through the Arene Substituent

A third alternative mechanism, based on activation via coordination to the metal center through an arene substituent, has been proposed for systems involving vinylarenes or vinylpyridines.^47^ The catalytic cycle starts with the coordination of the unsaturated substrate to the metal (Ru, Fe) through the arene moiety yielding the catalytic species (**J**). In this step the vinyl group is activated for the nucleophilic addition of the amine. After the nucleophilic addition of the amine on complex **J**, intermediate **K** is formed. Protonation of the aminoarene ligand gives rise to intermediate **L**. The catalytic species (**J**) is regenerated by the incorporation of a new reactant via arene exchange, which delivers the hydroaminated product (Scheme 6). The initial coordination of the unsaturated reactant is different for arene or pyridine substituents; the first is η^6^-coordinated to the aromatic ring, while the second coordinates through the N atom.

#### 2.1.4 Amine Activation Mechanism

For late transition metal catalysts, in addition to the previously described mechanisms, an alternative mechanism in which the amine coordinates to the metal center to generate a metal–amido complex has also been reported.^33^ Importantly, the generation of the metal–amido complex may involve the formal oxidation of the metal center. This is the basis of the seminal work by Hartwig to activate ammonia.^48^ Then, the unsaturated substrate (alkene or alkyne) inserts into the metal–amido bond. The regioselectivity of the process is decided in this step.

**Scheme 7:**
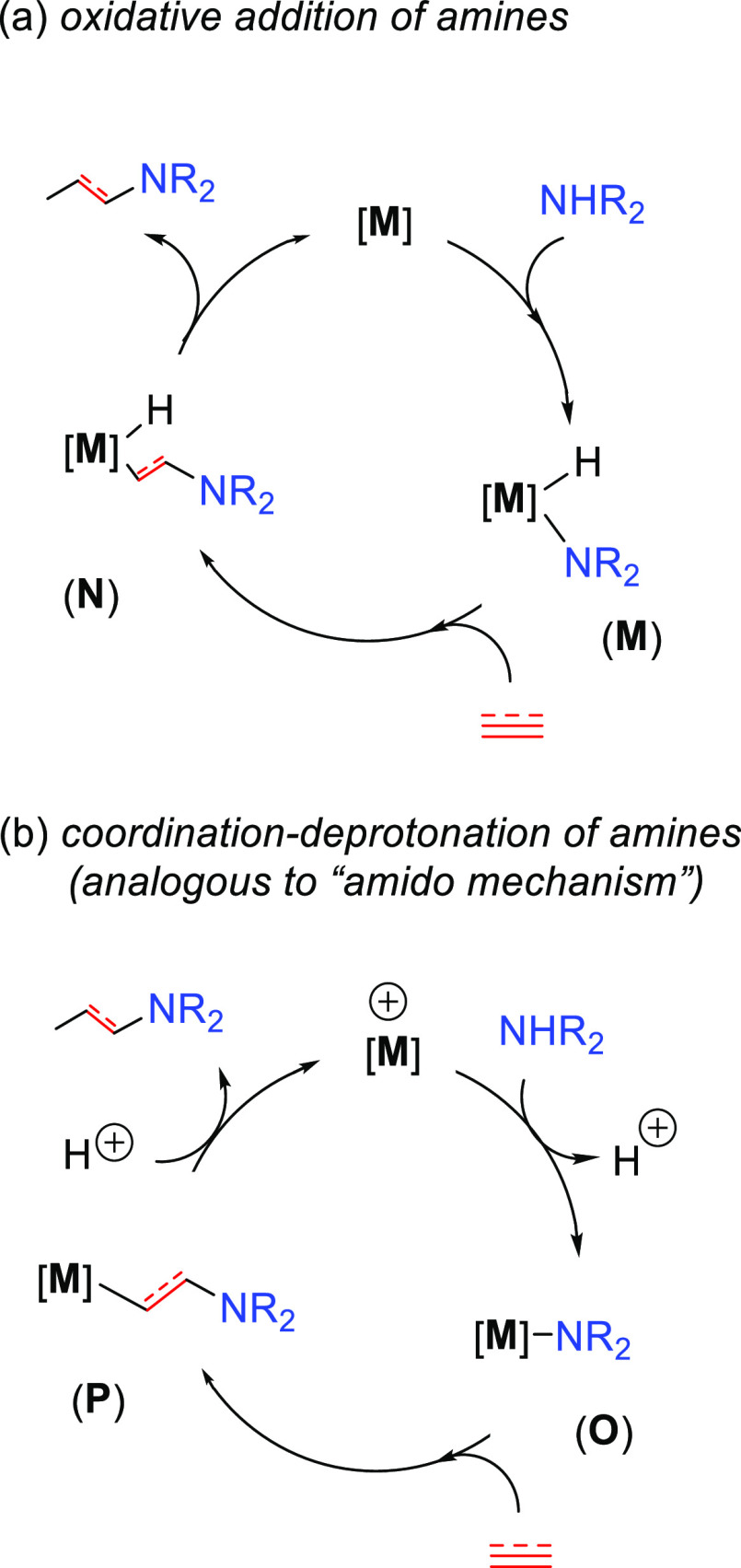
Two Proposed Hydroamination Pathways Starting with Amine Activation: (a) Oxidative Addition of Amines; (b) Coordination–Deprotonation of Amines.

The amine activation to generate the metal–amido intermediate can be described by two different pathways (Scheme 7): (a) oxidative addition of amine to produce an intermediate containing a hydride and an amido group (**M**) and (b) coordination and deprotonation of the amine to the metal center to generate the metal amide intermediate (**O**). Then there is the insertion of the unsaturated substrate into the M–N bond; it generates intermediates **N** and **P** for pathways (a) and (b), respectively. Finally, the last step is different for each mechanism. For pathway (a), there is a reductive elimination involving the hydride and the ligand obtained by the insertion of the unsaturated substrate to the amide, intermediate **N**, thus delivering the desired aminated product from intermediate **N**. For pathway (b), instead, the product is formed by protonating the aminated intermediate **P**. These two pathways are represented in Scheme 7. Pathway (b) is analogous to that described in the first subsection as the “amido mechanism”; therefore, it will be named as such in this review. Pathway (a), involving the oxidative addition of amine to the metal center, was included here for the sake of completeness since it has been proposed in Markovnikov hydroamination reactions.^33,49^ Nevertheless, none of the anti-Markovnikov hydroamination catalytic processes described in this review were found to follow the oxidative addition of the amine (Scheme 7a). Instead, reactions involving amine activation are better suited within the amido mechanism described in a previous subsection.

### 2.2 Mechanism of Formal Hydroamination Reactions

Hydroamination is highly attractive from a synthetic point of view, as no intrinsic byproducts are formed in the reaction media involving high atom economy. Whereas this is true for direct hydroamination processes whose mechanisms are described in the previous subsection, other developed hydroamination processes do not fulfill such atom economy. These strategies may combine several reagents and/or reactions in order to obtain the formal anti-Markovnikov adduct. The objective of these strategies is generating an intermediate from the unsaturated (alkene or alkyne) reactant that is prone to subsequently incorporate a nucleophile in an anti-Markovnikov fashion. Among these strategies, here we selected those that were specifically designed to convert alkenes and/or alkynes to the corresponding anti-Markovnikov hydroaminated products. In all these processes, the species providing the “H” and the “amino” moieties for the formal hydroamination originate from different reactants. Such a combination of reactions generates the anti-Markovnikov product, whereas direct hydroamination either would not work or would give the Markovnikov product. These procedures could also be classified according to their mechanism: the hydroamination is accomplished involving sequential, multistep processes, or a single catalytic reaction (but using reagents other than just an amine nucleophile). In the former, the initial reaction entails some other functional group that drives the conversion into a formal anti-Markovnikov hydroaminated product. These strategies are described in the following subsections, depending on the overall process involving a single catalytic reaction or a sequential multistep process.

#### 2.2.1 Sequential Multistep Reactions

In these processes the initial catalytic reaction incorporates some other functional group that is subsequently converted into an amine.

##### 2.2.1.1 Hydroboration–Electrophilic Amination

**Scheme 8:**
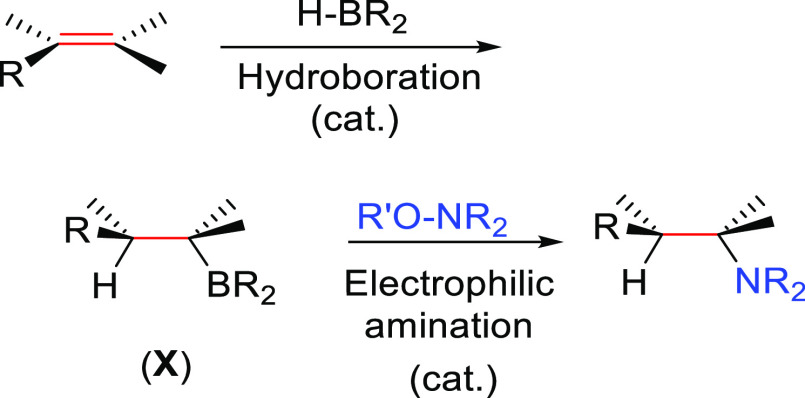
General Scheme for the Hydroboration–Electrophilic Amination Process to Produce Formal Anti-Markovnikov Hydroamination.

The early methods to obtain anti-Markonikov hydroaminated products were based on employing a hydroboration-amination sequence (Scheme 8). Among the most useful methods initially developed to obtain primary amines are those described by Brown^50^ in the 1960s and Kabalka^51^ in the 1980s, respectively, but they are not catalytic processes. Secondary amines can be obtained by the reaction of a Lewis acid (BF_3_ or SnCl_4_) and alkyl azide with boronic esters (previously converted to trifluoroborates).^52^ The regioselectivity of the process is defined during the hydroboration process: the boron moiety is added to the less substituted carbon atom of the unsaturated substrate, forming intermediate **X**, and then, the boron is substituted by the amine.

More recently, catalytic methods have been developed for each of the two involved reactions. The processes described here involve catalysis for one or both reactions involved (see below).

##### 2.2.1.2 Oxidation–Reductive Amination

**Scheme 9:**
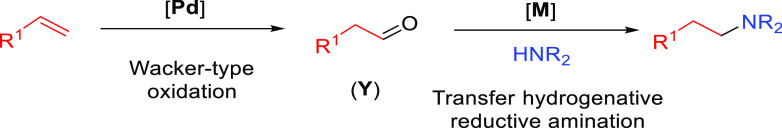
General Scheme for the Oxidation–Reductive Amination Process to Produce Formal Anti-Markovnikov Hydroamination

This process consists of the combination of oxidation of the alkene followed by a reductive amination of the aldehyde intermediate **Y** (Scheme 9). The first reaction corresponds to the oxidation of the terminal alkene to generate the corresponding aldehyde. Next, the carbonyl group reacts with the amine, producing an imine/iminium species. Subsequently, the imine is catalytically reduced by means of a transfer hydrogenation reaction generating the formal hydroaminated product. In this mechanism, it is of utmost importance that the catalyst effectively catalyzes the hydrogen transfer process.

In the development of one pot oxidation–reductive amination processes, the catalysts must fulfill some requirements. The initial catalytic oxidation of terminal alkenes should give the aldehyde trying to avoid the formation of ketone (Scheme 9). Moreover, the chemoselectivity of the reduction step must support the formation of the hydroamination product rather than the alcohol. On the other hand, the formation of the reductive amination product (R-CH_2_NR_2_), by the reaction of the amine (HNR_2_) with R-CH_2_OH (the source necessary for the hydrogen transfer reaction), should also be avoided.

#### 2.2.2 Single Catalytic Reactions

 

##### 2.2.2.1 Hydrometalation–Electrophilic Amination

**Scheme 10:**
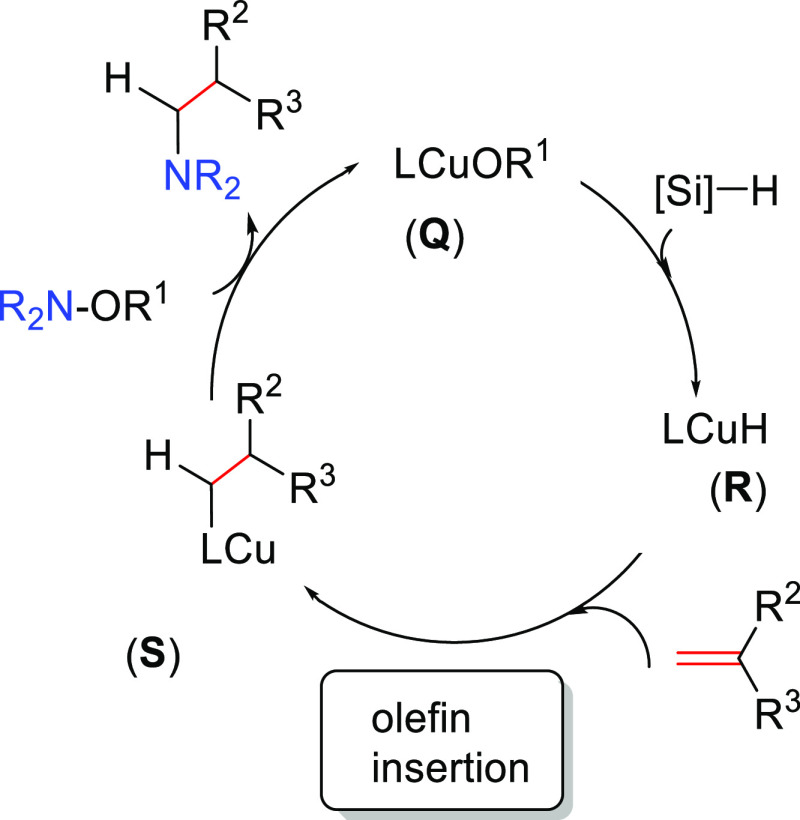
General Scheme for the Hydrometalation–Electrophilic Amination Process to Produce Formal Anti-Markovnikov Hydroamination

In this process, the sources of N and H in the formal hydroamination are from different reactants. For instance, hydrogen comes from hydrosilanes (as a hydride) whereas nitrogen comes from hydroxylamines (as R_2_N^+^). This umpolung approach that separates the H^–^ and R_2_N^+^ portions into different reagents entails thermodynamic advantages. This allows for each step of hydroamination to be strongly downhill, which in turn forces hydrometalation to be selectivity-determining and allows for good stereocontrol.^53^ This mechanism has been described to date only for Cu(I) catalysts.^54^ The mechanism for the hydrometalation–electrophilic amination is depicted in Scheme 10. Under the reaction conditions, the copper(I) alkoxide species LCuOR (**Q**) is generated; subsequently, it undergoes a transmetalation with the hydrosilane forming a LCuH complex (**R**). This is the key complex of the reaction; it undertakes the hydrometalation of the unsaturated substrate, giving rise to the alkylcopper intermediate (**S**). The regioselectivity of the process is defined at this step. This intermediate reacts with electrophilic amines (R′O-NR_2_) to afford the hydroaminated product as well as regenerating the active copper(I) alkoxide (**Q**) as the starting species of the catalytic cycle.

This process was initially developed for the Markovnikov hydroamination of alkenes. Nevertheless, Buchwald and co-workers realized that the regioselective outcome highly depends on the substrate employed.^54^ Hence, they discovered that terminal alkenes and alkynes with aliphatic substituents give rise to the anti-Markovnikov hydroamination product, and even a chiral version was developed employing the proper chiral ligand.

##### 2.2.2.2 “Hydrogen Borrowing” Process

**Scheme 11:**
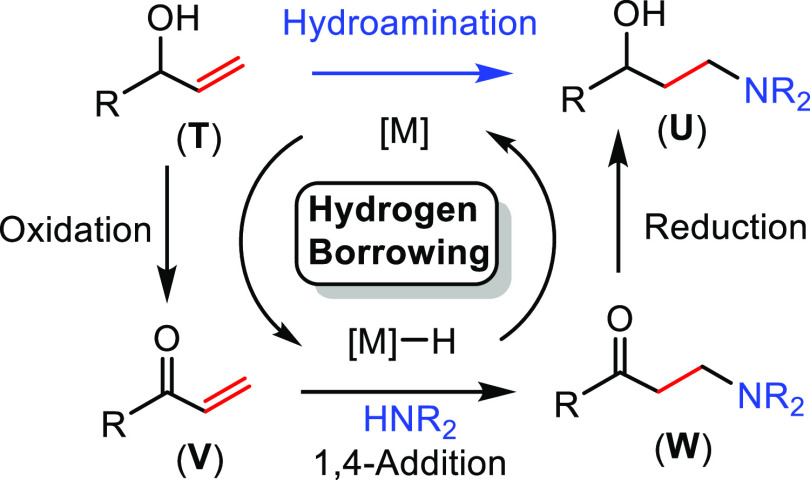
General Scheme for Hydroamination via the “Hydrogen Borrowing” Process

This synthetic methodology is called the “hydrogen borrowing process” because the reactant transfers a hydrogen molecule to the catalyst in the first step, while in the last step the hydrogen molecule is returned to the substrate (Scheme 11). The method consists of the combination of three different reactions: first, an initial oxidation of the allylic alcohol (**T**) to generate an α,β-unsaturated carbonyl compound (**V**); second, a 1,4-conjugate addition to yield a β-amino carbonyl compound (**W**); and third, a reduction of the ketone to regenerate the γ-amino alcohol (**U**). The metal catalyst is involved in the hydrogen borrowing process by taking and returning a formal H_2_ molecule from and to the alcohol group. In fact, this tandem reaction has only been described for allylic alcohols (Scheme 11).

## 3 Metal-Based Catalysts for Anti-Markovnikov Hydroaminations

### 3.1 Alkali Metals

Alkali metals (group 1) are among the most abundant elements in Nature. In this regard, sodium and potassium are the sixth and seventh most abundant elements on Earth, respectively, whereas lithium and rubidium can be found in the Earth’s crust in concentrations of 17 and 60 ppm, respectively. Cesium is very rare and can be found at 3 ppm, and francium is not found naturally, except in some uranium ores.^55^ However, despite their abundance, the application of group 1 derivatives, based on lithium, sodium, or potassium, as catalysts in modern synthetic organic chemistry is limited to a few reactions such as ester exchange reactions,^56^ hydroboration of carbonyl compounds,^57^ C–H bond silylation,^58^ and dehydrogenation,^59^ among others. In this regard, the base-mediated hydroamination is of high interest due to the low cost of the alkali metal catalysts in comparison with the more expensive and less abundant transition metals and lanthanides. Alkali base catalysts can easily deprotonate the amines, providing more nucleophilic species which can efficiently attack the unsaturated compound.

#### 3.1.1 Alkali-Based Catalysts

**Figure 2:**
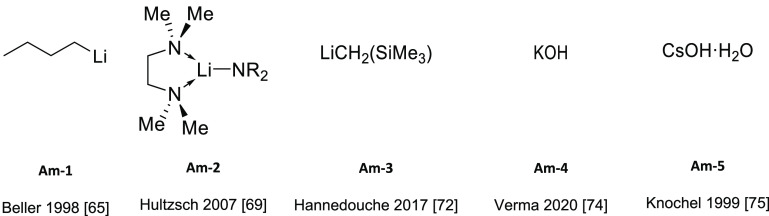
Alkali metal catalysts and ligands featuring anti-Markovnikov regioselectivity.

The most representative catalysts based on alkali metals used in hydroamination reactions of olefins and alkynes selectively affording the anti-Markovnikov product are displayed in Figure 2; the experimentally unsaturated reactant amines and additives, if any, are summarized in Table 1.

**Table 1:**
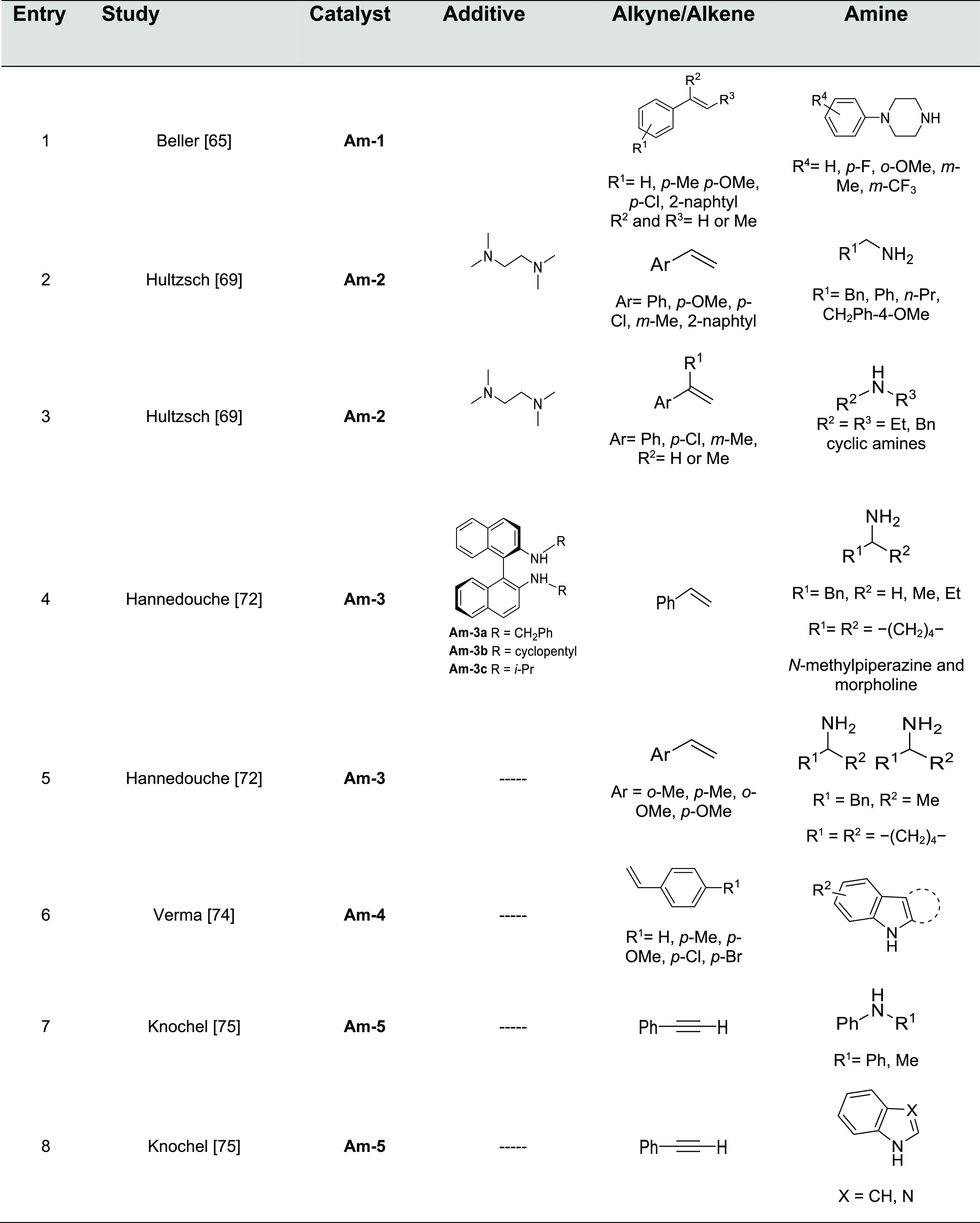
Experimentally Characterized Alkali Metal-Catalyzed Intermolecular Hydroaminations with Anti-Markovnikov Regioselectivity

**Scheme 12:**
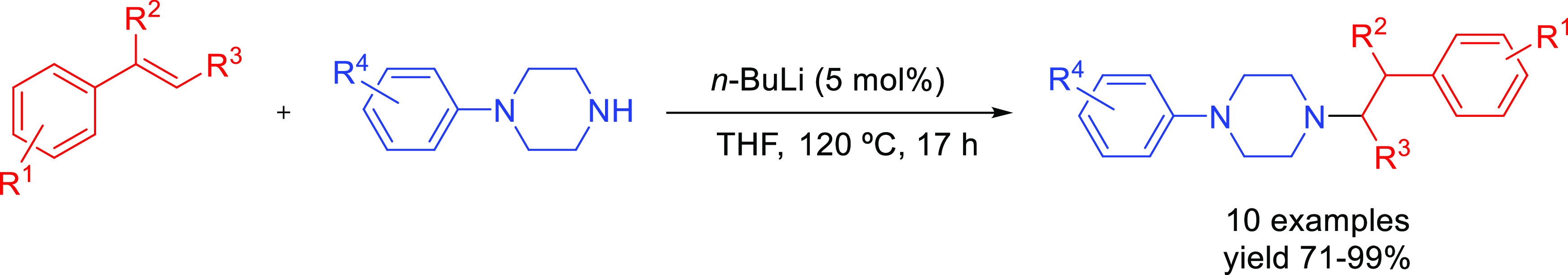
Intermolecular Hydroamination of Vinylarenes with *N-*Arylpiperazines Catalyzed by Alkali Metals^66^

The first example of the use of alkali metals in hydroamination reactions was reported in the early 1950s. It described the reaction of primary and secondary amines with alkenes in the presence of alkali metals or their hydrides under harsh reactions conditions, such as temperatures above 175 °C and pressures around 400 atm.^60−62^ Despite the fact that several works continued the exploration of alkali metals in hydroamination reactions,^63,64^ it was not until the late 1990s when the group of Beller explored the scope of the alkali metal-catalyzed anti-Markovnikov hydroamination of vinylarenes with primary and secondary amines; they accomplished the reaction under relatively mild conditions (Table 1, Entry 1).^65^ Hydroamination products were obtained in excellent yields and exclusive selectivity for the anti-Markovnikov adduct when performing the reaction in the presence of 5 mol % *n*-butyllithium (**Am-1**) in THF as solvent (120 °C, 17 h). Strong solvent effects were observed, and polar and coordinating solvents, such as THF, showed superior catalytic activity compared to *n*-hexane or toluene, which was attributed to the formation of more reactive lithium piperazide species. The use of less basic alkali metal catalysts such as lithium *tert*-butoxide or potassium *tert*-butoxide failed to give the hydroamination product. By using this methodology, Beller and co-workers prepared biologically active amphetamine derivatives, in which the initial synthetic step was an anti-Markovnikov addition of aryl-substituted piperazines to styrenes (Scheme 12).^66^

In a later study, Beller and co-workers studied the use of *N*,*N*,*N′*,*N′*-tetramethylethylenediamine (TMEDA) as an additive to alkyl lithium derivatives in the hydroamination of ethylene with diethylamine.^67^ The use of this nitrogen-containing ligand increased the reaction rate for the hydroamination, and the reaction could be performed at lower temperature and pressure (2.5 mol % LiNEt_2_, toluene, 80 °C, 12 h). Mechanistic studies suggested deactivation of the active lithium amide catalysts, that was not correlated to the TMEDA degradation, but to formation of LiH; the latter species is assumed to be formed by β-hydride elimination from lithium diethylamide species.

In a later work published in 2006, Hultzsch and co-workers reported the first example of the asymmetric intramolecular hydroamination promoted by chiral-lithium-based catalysts.^68^ The authors synthesized a chiral diamidobinaphthyl dilithium salt from coupling of BOC-l-proline and racemic diaminobinaphthyl and subsequent reduction and diastereomeric separation, followed by a final lithiation step of the (*S*,*S*,*S*)-derivative. Using these lithium amide catalysts (5 mol %, C_6_D_6_, argon atmosphere), hydroamination/cyclization reactions of aminoalkene proceed at room temperature with high yields and enantioselectivities up to 75% ee. A catalyst based on LiN(SiMe_3_)_2_ and (−)-sparteine was found to improve the yield. In an extension of this work, Hultzsch and co-workers studied the intermolecular hydroamination of vinylarenes and the influence of additives on the catalyst activity.^69^ The authors found that catalyst **Am-2**, formed by the combination of LiN(SiMe_3_)_2_ and TMEDA (Figure 2), enhanced the reaction rate and in few cases; hydroamination took place at room temperature (10 mol %, THF) or even in solvent-free conditions (Table 1, Entry 2). In all cases, the anti-Markovnikov secondary amine monoadduct was exclusively formed as the major product (Table 1, Entry 3). However, in some cases the tertiary amine bis-adduct was formed as a byproduct, although the use of high amine/vinylarene ratios diminished the formation of this byproduct.

**Scheme 13:**
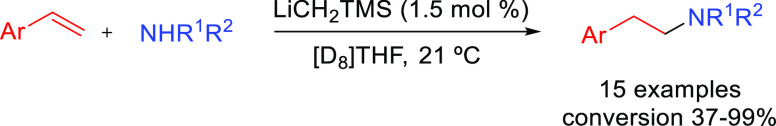
Intermolecular Hydroamination of Vinylarenes with Secondary Amines Catalyzed by Li Compounds^72^

In their investigation of intramolecular hydroamination reactions, Hannedouche and co-workers found that easily available chiral diaminobinaphthyl ligands in combination with alkali metal derivatives (MeLi, *n-*BuLi, or LiCH_2_SiMe_3_ = **Am-3**) formed efficient precatalysts (diamidobinaphthyl dilithium salts) for the asymmetric cyclohydroamination of amino-1,3-diene compounds at room temperature with high stereoselectivities and moderate enantioselectivities (Table 1, Entry 4).^70,71^ Further studies on lithium-catalyzed intermolecular hydroamination of vinylarenes with secondary amines using chiral diamidobinaphthyl dilithium salts as catalysts (LiCH_2_SiMe_3_ 8 mol %, ligand 3.2 mol %, C_6_D_6_, 100 °C, 5–24 h) led to the formation of the anti-Markovnikov hydroamination product (Scheme 13).^72^ Interestingly, higher conversions were obtained in deuterated benzene when using a racemic mixture of the diaminobinaphthyl ligand or even in its absence, which was rationalized in terms of the formation of inactive lithium amide aggregates. Furthermore, a strong solvent effect was also observed. In this regard, the use of polar and coordinating solvents, such as THF, afforded higher conversions, as this type of solvents may reduce the formation of lithium aggregates in solution. With this ligand-free methodology and using LiCH_2_SiMe_3_ as a base (**Am-3**) (1.5 mol %) in deuterated THF (Table 1, Entry 5), β-arylethylamines could be obtained in high yields (up to 99%) with short reaction times (from 15 min to 2 h). Longer reaction times were required for α-methylstyrene and allylbenzene, which also required higher base loading (8 mol %); however, α,β-disubstituted olefins were unreactive toward secondary amines under the described reaction conditions.

**Scheme 14:**
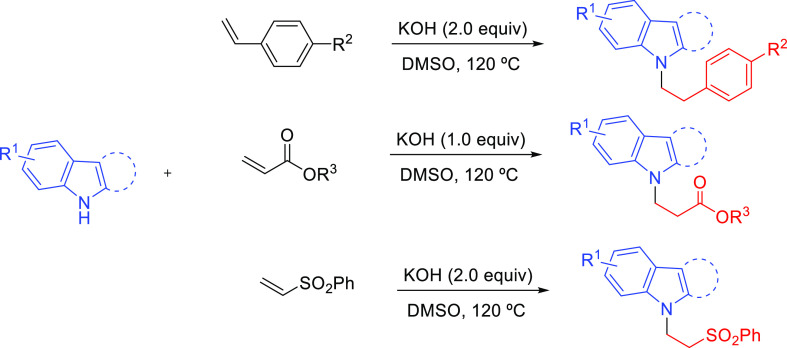
KOH Promoted Intermolecular Hydroamination of Styrenes, Acrylates, and Sulfones with Indole/Carbazoles^74^

The Verma group has been very active in the investigation of inter- and intramolecular hydroamination reactions of alkynes in the past decade.^73^ In a recent publication, they reported base-induced selective synthesis of *N-*alkylated heterocycles by KOH-catalyzed hydroamination of indole with styrenes, acrylates, and sulfones (**Am-4**, Table 1, Entry 6).^74^ This strategy (3.0 equiv of KOH, DMSO, 120 °C, 36–42 h) afforded the *N-*alkylated alkenes with exclusive anti-Markovnikov selectivity with good to excellent yield without forming the C-3 addition product (Scheme 14). The use of other alkali bases such as NaOH or CsOH provided the anti-Markovnikov product with lower yields. Based on deuterium labeling studies, they showed that hydroamination of styrene incorporates deuterium on the product at the α-carbon adjacent to the phenyl/ester group. They suggested that the reaction proceeds through hydroamination with the incoming protons provided by the solvent. Deuterated solvent is proposed to participate by a deuterium exchange between [D_6_]DMSO and H_2_O.

The use of heavier alkali metals as catalysts in the intermolecular hydroamination of alkynes with anti-Markovnikov regioselectivity has also been described. In an early work reported by Knochel and co-workers in 1999, cesium hydroxide was shown to catalyze (20 mol %) the addition of alcohols and secondary aromatic (**Am-5**, Table 1, Entry 7) or heterocyclic amines (**Am-5**, Table 1, Entry 8) to phenylacetylene in *N*-methyl-2-pyrrolidone (NMP) as solvent at 120 °C, yielding enol ethers and enamines in good to high yields (60–91% in 12–24 h).^75^

#### 3.1.2 Mechanistic Considerations and Factors Governing the Regioselectivity

**Scheme 15:**
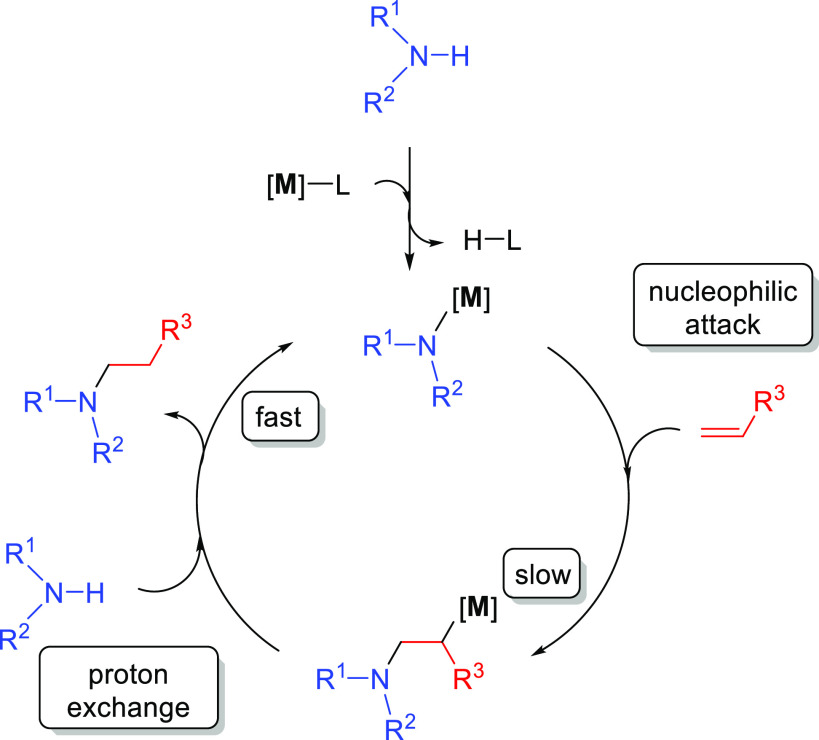
Catalytic Cycle for the Base-Catalyzed Hydroamination of Alkenes; **M** = Alkali Metal

The general catalytic cycle for the base-catalyzed hydroamination of alkenes is similar among all proposed cases. It starts with the deprotonation of the amine by the alkali metal derivative (**M**–L) to give a metal amide, which can undergo a nucleophilic attack on the alkene (Scheme 15). The highly reactive 2-aminoalkyl metal complex formed in this step can suffer a proton exchange with a new amine, regenerating the metal amide and affording the hydroamination product.

**Figure 3:**
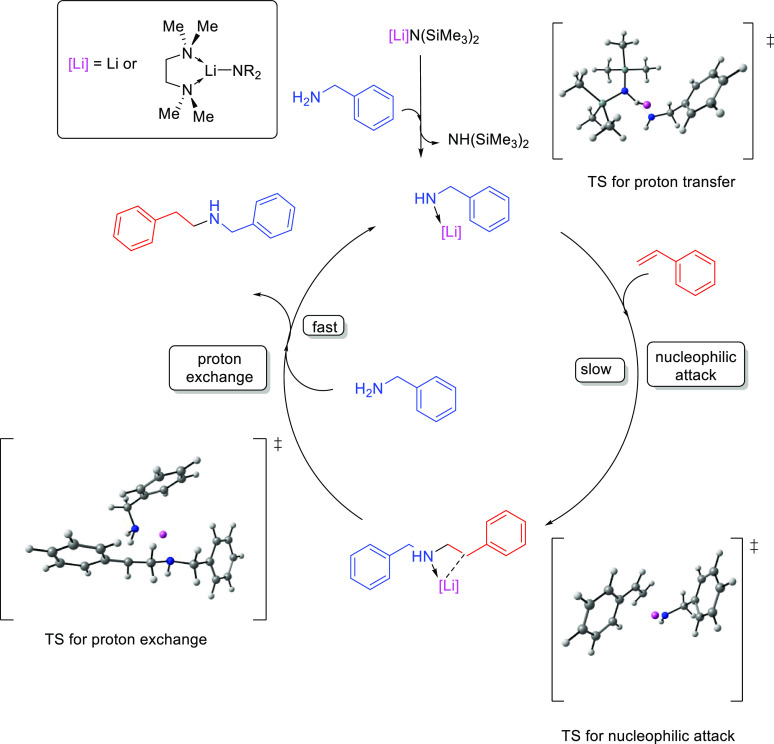
Proposed mechanism and optimized transition state structures for the hydroamination of styrene with benzylamine, catalyzed by LiN(SiMe_3_)_2_.^69^

In order to rationalize the formation of the anti-Markovnikov product, Hultzsch and co-workers investigated the different pathways with computational methods (DFT calculations with the B3LYP functional).^69^ The computed catalytic cycle for the hydroamination of vinylarenes with benzylamine, catalyzed by LiN(SiMe_3_)_2_ or (TMEDA)LiN(SiMe_3_)_2_, is based on an ionic mechanism involving three steps (Figure 3). The first step consists of a proton transfer from benzylamine to lithium bis(trimethylsilyl)amide to form the lithium benzylamide catalyst. This initial step is endergonic (Δ*G* = 6.9 kcal mol^–1^) and involves a late transition state in which the lithium atom is interacting with both nitrogen atoms, while the proton of benzylamine is almost transferred to the nitrogen atom of [Li]N(SiMe_3_)_2_. Lithium bis(trimethylsilyl)amide deprotonates benzylamine to afford a strongly nucleophilic metal amide, which can easily add to the olefin in the second step of this mechanism. This nucleophilic attack via the Markovnikov or anti-Markovnikov pathway determines the regioselectivity of the process. According to DFT calculations, the interaction between the lithium ion and the aromatic ring in the anti-Markovnikov transition state has been proposed to be the origin of the regioselectivity. In this regard, the aromatic ring of the styrene plays an important role because its interaction with the lithium ion governs the regioselectivity of the process by favoring the anti-Markovnikov mechanism. Overall, the anti-Markovnikov addition is kinetically and thermodynamically favored over the Markovnikov addition. The nucleophilic attack affords the 2-aminoalkyl metal complex stabilized by an intramolecular amine coordination to the lithium ion. The third and last step involves an exchange of lithium by a proton from benzylamine at the intermediate compound, delivering the anti-Markovnikov hydroamination product and regenerating the initial lithium benzylamide catalyst.^69^

### 3.2 Alkaline Earth Metals

Alkaline earth elements have been used as catalysts and reagents in many organic reactions for more than a century.^76^ The group 2 elements are highly attractive, as two of them, calcium and magnesium, are among the 10 most abundant elements in the Earth’s crust, which makes them low cost and environmentally benign materials. The chemistry of these metals is mainly dominated by the highly stable +2 oxidation state,^77^ although a few stable magnesium(I) compounds have been described in the past decade.^78−80^ Group 2 elements have some analogies with other d- and f-block metal complexes, for instance with the trivalent d^0^ organolanthanide compounds (L_2_LnX complexes), as the +2 oxidation state of alkaline earth elements provides them an effective d^0^ electronic configuration. In the case of hydroamination reactions, the first step of the catalytic cycle involves the insertion of the olefin into the alkaline earth metal–amide bond followed by a rapid protonolysis. Findings by Harder and co-workers demonstrated the formation of dibenzylcalcium complexes capable of mediating insertion chemistry and acting as initiators for the living polymerization of styrene.^81^ Inspired by this work, heteroleptic, four-coordinate group 2 complexes attracted researchers’ attention on catalyzed heterofunctionalization of unsaturated moieties, similarly to lanthanide catalysts.

#### 3.2.1 Alkaline Earth-Based Catalysts

**Scheme 16:**
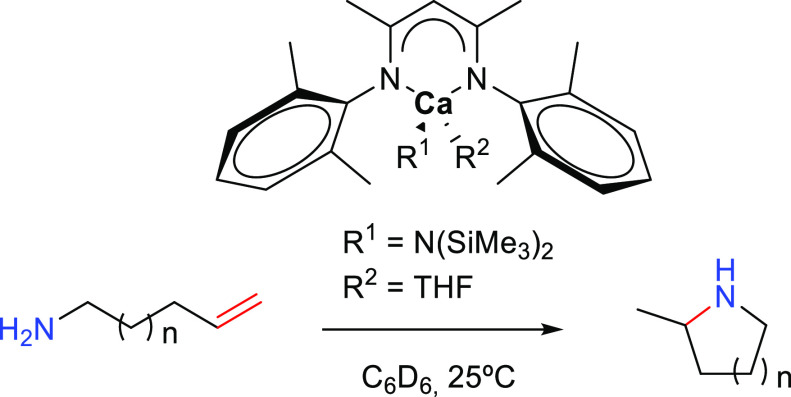
Calcium-Catalyzed Intramolecular Hydroamination of Aminoalkenes^82^

The first example of the use of alkaline earth elements as hydroamination catalysts was reported by Hill and co-workers in 2005 (Scheme 16) and consisted in an intramolecular hydroamination/cyclization of aminoalkenes catalyzed by a calcium bidentate β-diketiminate complex (10 mol %, THF, 25 °C).^82^ In a subsequent work, Hill and co-workers performed mechanistic studies by extending the use of group 2 elements to magnesium, calcium, and strontium (2–10 mol %, C_6_D_6_ or toluene-*d*_8_, 25–80 °C), in combination with a modification of the previously described β-diketiminato ligand.^83^ These reactions enable the synthesis of five-, six-, and seven-membered heterocyclic compounds with Markovnikov configuration under smooth reaction conditions, which are faster for five-membered rings. The mechanistic studies also revealed a higher catalytic activity for the calcium complex in comparison to the magnesium analogue.

**Figure 4:**
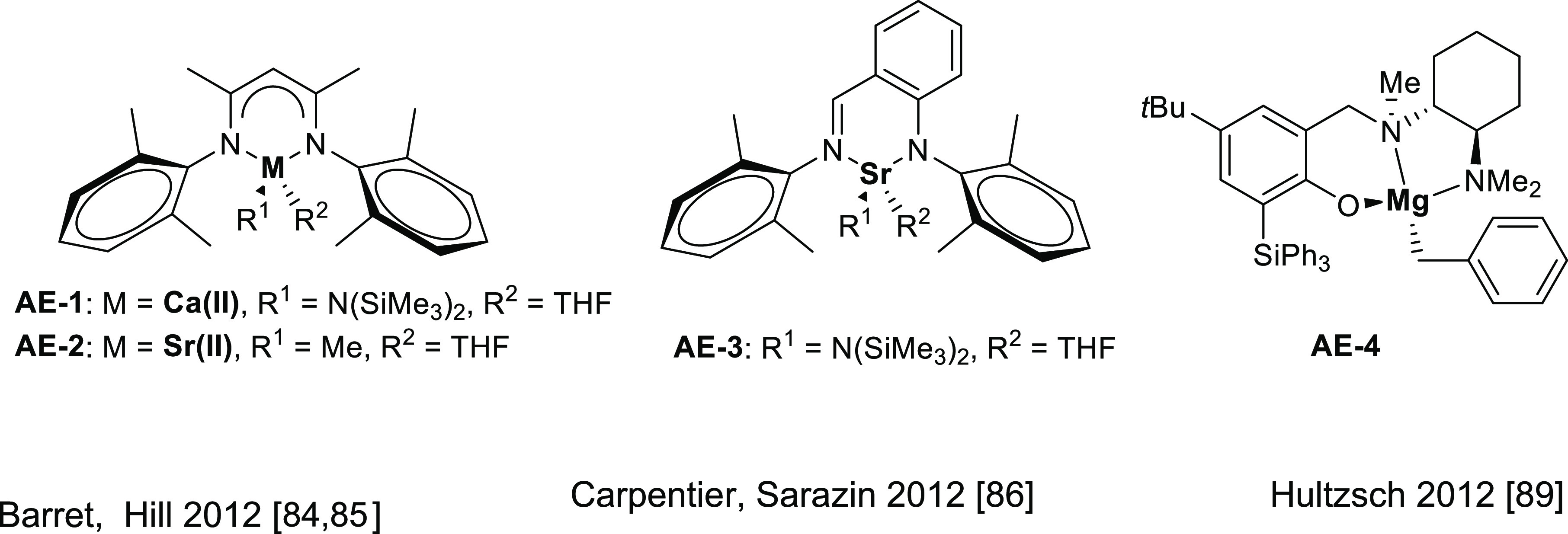
Alkaline earth catalysts featuring anti-Markovnikov regioselectivity.

In 2009, the first example of an intermolecular anti-Markovnikov catalysis mediated by a d^0^ alkaline earth center was provided by Barret, Hill, and co-workers describing the hydroamination of vinyl arenes with primary, secondary, and *N*-heterocyclic amines promoted by homoleptic calcium and strontium amides (Table 2, Entry 1).^84^ The experimentally characterized alkaline earth catalysts for intermolecular anti-Markovnikov hydroaminations are presented in Figure 4.

**Table 2:**
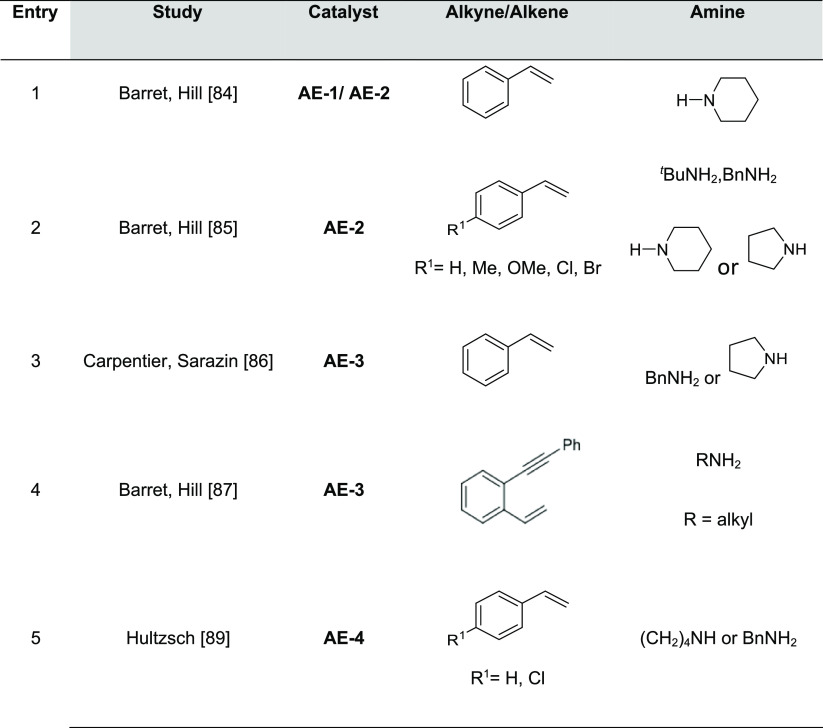
Experimentally Characterized Alkaline Earth Metals-Catalyzed Intermolecular Hydroaminations with Anti-Markovnikov Regioselectivity

Strontium catalysts for the intermolecular variant of the hydroamination of vinylarenes and dienes were found to be active under mild conditions (5 mol % catalyst, neat, 60 °C).^85^ In particular, the intermolecular Sr-catalyzed hydroamination with **AE-2** was found to be faster than that promoted by the calcium homologue **AE-1** catalyst, and a wider scope was evaluated including primary and secondary amines (Table 2, Entry 2). A few years later, Carpentier, Sarazin, and co-workers used heteroleptic catalyst **AE-3** (Table 2, Entry 3) in the hydroamination of styrene with aliphatic amines (0.1–2 mol % catalyst, neat, 60 °C).^86^ Later, Barret, Hill, and co-workers using the same catalyst **AE-3** could access more complex chemical structures, such as tetrahydroisoquinoline frameworks through the strontium-catalyzed addition of primary amines to 1,5-enynes (10 mol % catalyst, C_6_D_6_, 130 °C). This was accomplished by a series of intermolecular anti-Markovnikov alkene hydroaminations with primary alkyl amines and subsequent intramolecular alkyne hydroaminations (Table 2, Entry 4).^87^

Hultzsch and co-workers initially developed a phenoxyamine magnesium catalyst for intramolecular hydroamination of aminoalkenes. Importantly, this catalyst was able to avoid ligand redistribution, that is crucial for developing chiral reactions.^88^ Based on this knowledge, they were able to develop a chiral magnesium catalyst, **AE-4**, that achieves enantioselectivities up to 93%.^89^ This catalyst is also able to perform intermolecular anti-Markovnikov hydroamination of vinyl arenes with secondary amines (5 mol % catalyst, neat, 60–80 °C) (Table 2, Entry 5). The reaction mechanism of the intramolecular version was studied by the same group.^90^

#### 3.2.2 Mechanistic Considerations and Factors Governing the Regioselectivity

**Scheme 17:**
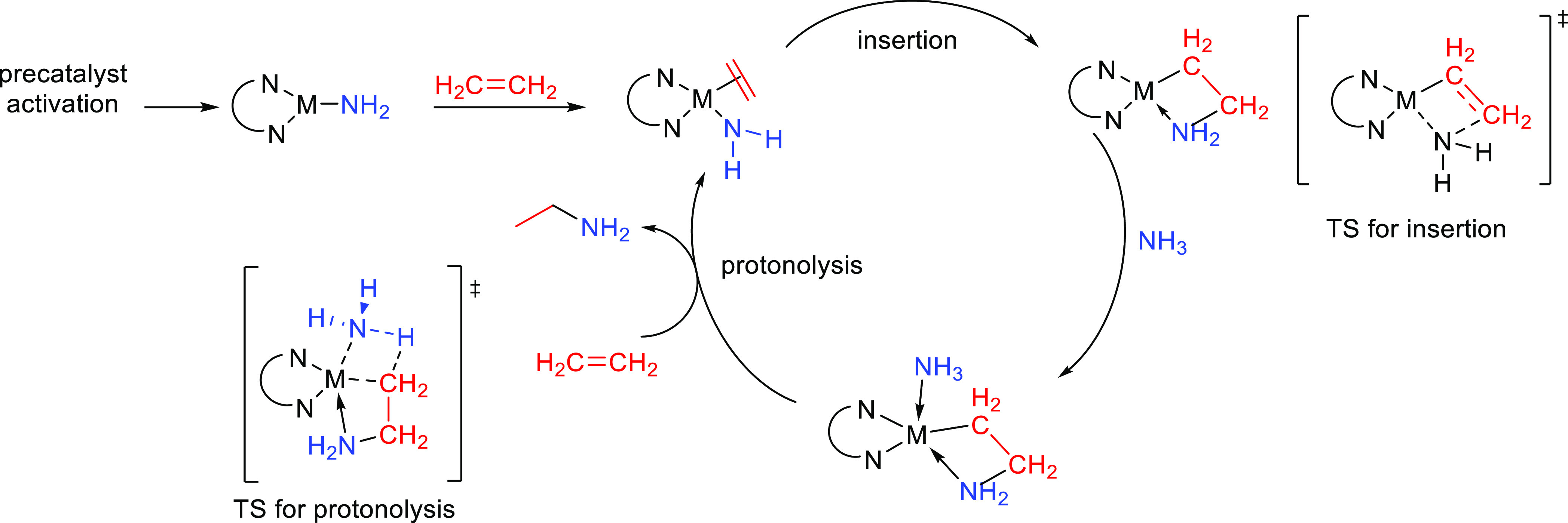
Proposed Catalytic Cycle for Hydroamination Catalyzed by Group 2 Metals via the σ-Insertive Mechanism^84^

Barret, Hill, and co-workers studied the hydroamination catalyzed by group 2 metals (Mg, Ca, Sr, and Ba) by means of density functional theory (DFT) calculations using the B3LYP functional and a 6–311G(d,p) basis set.^84^ Despite the fact that their previous synthetic works were focused on the intramolecular process, in this computational study a simple model system was used to evaluate the intermolecular reaction by replacing the aminoalkene by ammonia and ethene (Scheme 17). The ancillary β-diketiminato ligands were also simplified by changing the two N-aryl substituents by H atoms. After the precatalyst activation, the catalytic cycle starts with the rate-determining step, which was found to be the alkene insertion; it is dominated by Coulomb interactions between the carbon atom adjacent to the group 2 metal and the nitrogen. Alkene insertion into the M–N bond occurs via a four-center transition state that is highly polarized, and a protonolysis step forms the new C–N bond via a six-membered transition state involving the two substrates.

**Scheme 18:**
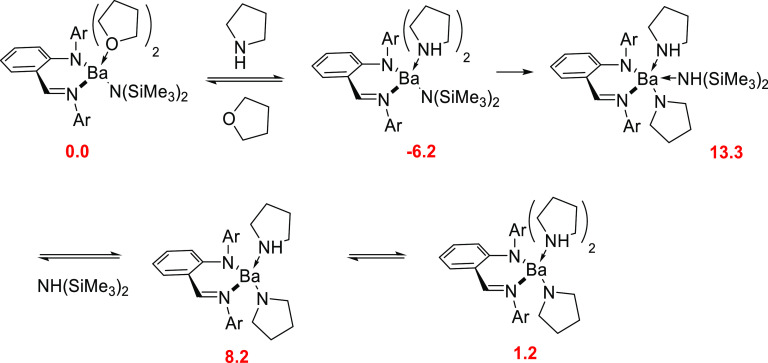
Catalyst Activation for Iminoanilide Barium Species ^91^ Relative Gibbs energies of intermediates (in red) are given in kcal mol^–1^.

A detailed computational study (DFT calculations using dispersion-corrected B97-D3 with basis sets of triple-ζ quality def2-TZVPP) of the mechanism of the intermolecular hydroamination of vinylarenes by a group 2 catalyst with an anilido-imine ligand was reported by Tobisch in 2014.^91^ In this mechanistic study, three different pathways were evaluated: (i) the classic stepwise σ-insertive mechanism, (ii) a concerted proton-assisted insertive concerted mechanism, and (iii) a stepwise proton-assisted insertive concerted mechanism. The first step, common to the three studied mechanisms, involves the catalyst activation with the conversion of the THF-coordinated iminoanilide barium catalyst into the corresponding pyrrolide compound, which is the catalytically competent species. Calculations showed that both THF and pyrrolidine bind more strongly than styrene and despite the steric hindrance of the iminoanilide ligand, it can accommodate two THF or pyrrolidine molecules. The initial metal anilido-imine precatalyst is coordinated to two molecules of THF and bis[bis(trimethylsilyl)amido] ligand [N(SiMe_3_)_2_], but in the presence of pyrrolidine the coordinated THF molecules are replaced by two amines in an exergonic reaction. Next, a metal–N aminolysis takes place through a metathesis type TS yielding the metal pyrrolide catalyst with the liberation of bis(trimethylsilyl)amine [HN(SiMe_3_)_2_]. As shown in Scheme 18, this catalyst activation is essentially thermoneutral.

**Scheme 19:**
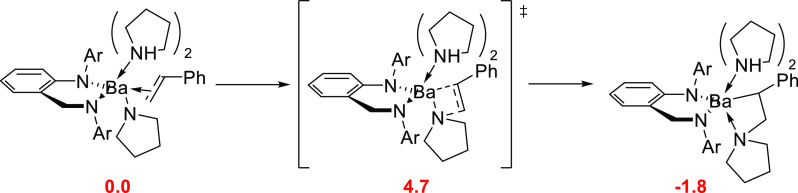
Key Stationary Points along the Most Accessible Pathway for Migratory Olefin Anti-Markovnikov Insertion into the Ba–N Pyrrolido σ-Bond ^91^ Relative Gibbs energies of intermediates (in red) are given in kcal mol^–1^.

DFT calculations showed that the multicentered transition state required by the proton-assisted concerted pathways was energetically inaccessible in comparison to the kinetically less demanding stepwise σ-insertive mechanism pathway. Therefore, the stepwise σ-insertive mechanism will be discussed below. The insertive mechanism starts with the olefin insertion into the Ba–N pyrrolido σ-bond through a four-membered planar TS structure in which Ba–N pyrrolide bonds are around 2.83 Å, and two adducted spectator pyrrolidine molecules are at 2.93 Å, whereas the one participating in the N···C bond formation is at 2.71 Å from the Ba center. This TS, with a low activation barrier of 4.7 kcal mol^–1^ and an N···C forming bond distance of 2.08 Å (Scheme 19), determines the regioselectivity of the reaction, favoring the anti-Markovnikov product over the Markovnikov with a Gibbs energy difference of 14.9 kcal mol^–1^. The formation of the bisamine adduct product is slightly exergonic, as the Ba–C and dative Ba–N bonds compensate the cleavage of the Ba–N bond in the pyrrolido reactant. Overall, the migratory olefin insertion into the Ba–N pyrrolido σ-bond proceeds strictly with an anti-Markovnikov regioselectivity through an equatorial C–C bond approach via a highly polarized planar four-center TS structure.

**Scheme 20:**
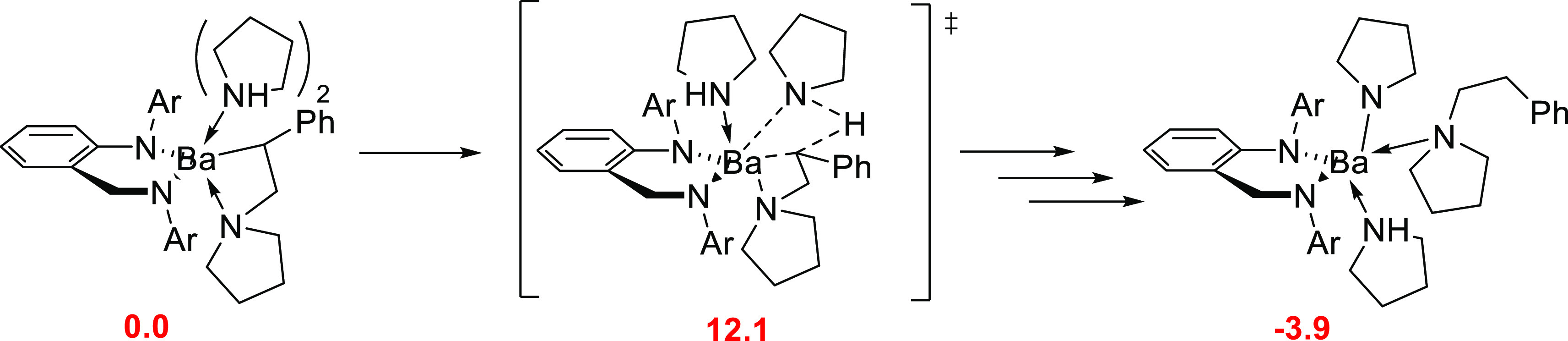
Key Stationary Points along the Most Accessible Pathway for Ba–C σ-Bond Aminolysis ^91^ Relative Gibbs energies of intermediates (in red) are given in kcal mol^–1^.

The next step, namely the Ba–C σ-bond aminolysis, involves an intramolecular hydrogen migration through a metathesis type transition state that describes the cleavage of an N–H bond and the formation of a C–H bond. In the TS structure, with a Gibbs activation barrier of 12.1 kcal mol^–1^, Ba–N pyrrolide bonds are 2.77 and 2.83 Å, and two adducted spectator pyrrolidine molecules are at 2.94 and 2.96 Å, whereas the one at the anti-Markovnikov product is located at 2.90 Å from the Ba center (Scheme 20).

Overall, computational examination of various alternative mechanistic pathways in the intermolecular hydroamination of styrene with pyrrolidine catalyzed by an alkaline earth catalyst concluded that the σ C–C insertive pathway displayed in Scheme 17 was energetically favored over the proton-assisted concerted pathway. Kinetic studies revealed that the reaction rates for the hydroamination of styrene with pyrrolidine increased across the series Ca < Sr < Ba. Large primary kinetic isotope effects were observed by Carpentier and Sarazin,^86^ and Hill,^85^ which were against the σ-insertive mechanism and supported a concerted insertion and proton transfer through a multicenter TS. Sarazin and co-workers proposed a proton-assisted 6-membered transition state involving the concerted C–N bond formation and protonolysis.^86^

### 3.3 Lanthanides

Lanthanides (Ln) are nontoxic and relatively abundant elements able to form organolanthanide complexes, which display a rich catalytic chemistry.^92^ They usually present only one stable oxidation state (+3), thus excluding reaction pathways via oxidative addition/reductive elimination. Ln centers feature high electrophilicity and kinetic lability, conferring high reactivity to Ln–E bonds (E = C, H, N, P). According to their characteristics, the most distinctive reactivity patterns of organolanthanides are the insertion of multiple C–C bonds into Ln–E bonds and σ-bond metathesis.^12,34,38,39,93,94^ These features have been exploited to effectively catalyze hydroamination processes. While the organolanthanides are very active catalysts for hydroamination, their sensitivity to oxygen and moisture as well as the lack of tolerance for functional groups have hampered their use in synthetic organic chemistry.

Rare earth metal complexes have been demonstrated to be highly efficient catalysts for intramolecular hydroamination (hydroamination/cyclization) of several multiple C–C bonds such as alkenes, alkynes, allenes, and dienes. In particular, intramolecular hydroamination of aminoalkenes is among the most thoroughly explored reactions in the category of hydroaminations.^2,32,34^ The study of intermolecular hydroamination reactions is more challenging, and consequently, it has been less investigated. The first examples were described by Li and Marks in 1996,^93^ who demonstrated that organolanthanide complexes of the type Cp_2_^′^LnCH(SiMe_3_)_2_ (Cp′ = η^5^-Me_5_C_5_; Ln = La, Lu, Sm, Nd) and Me_2_SiCp_2_^′′^LnCH(SiMe_3_)_2_ (Cp′′ = η^5^-Me_4_C_5_; Ln = Lu, Sm, Nd) served as efficient precatalysts for the regioselective intermolecular hydroamination of alkynes R′C≡CMe (R′ = SiMe_3_, Ph, Me), alkenes RCH=CH_2_ (R = SiMe_3_, CH_3_CH_2_CH_2_), butadiene, vinylarenes (ArCH=CH_2_), and methylenecyclopropanes with primary amines R′′NH_2_ to yield the corresponding amines and imines after 3 days (7.5 mol % cat, C_6_H_6_, 60 °C).^40,93^ Intermolecular reactions suffer from entropy penalty and are considerably slower than intramolecular ones. Compared with the intramolecular processes, these intermolecular hydroaminations are less kinetically favored, being around 350 and 1400 times slower for alkene and alkyne hydroamination, respectively. The intermolecular alkyne hydroamination was found to be between 3 and 7 times faster than the corresponding alkene reaction, under the same reaction conditions.^40^ A thorough review covers the most significant achievements in the use of organolanthanides for intermolecular hydroamination of multiple C–C bonds.^95^

A wide number of organolanthanide-catalyzed anti-Markovnikov hydroaminations have been reported in the past decade, and the most representative examples will be summarized and discussed in the following section.

#### 3.3.1 Lanthanide-Based Catalysts

**Figure 5:**
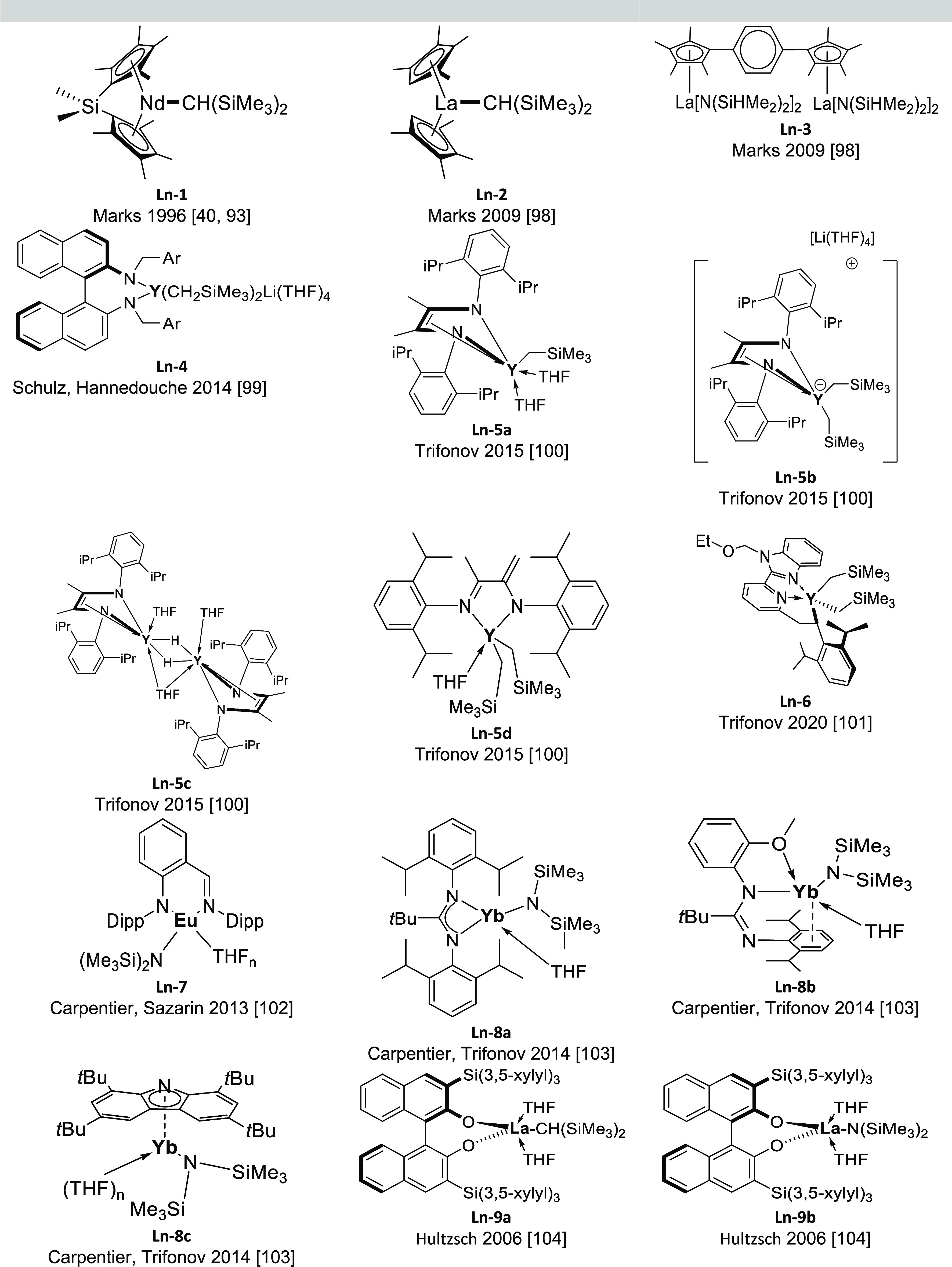
Organolanthanide catalysts featuring anti-Markovnikov regioselectivity.

Figure 5 shows the lanthanide catalysts used in hydroamination reactions that displayed anti-Markovnikov regioselectivity, labeled as **Ln-*x***. The corresponding reactions are collected in Table 3.

**Table 3:**
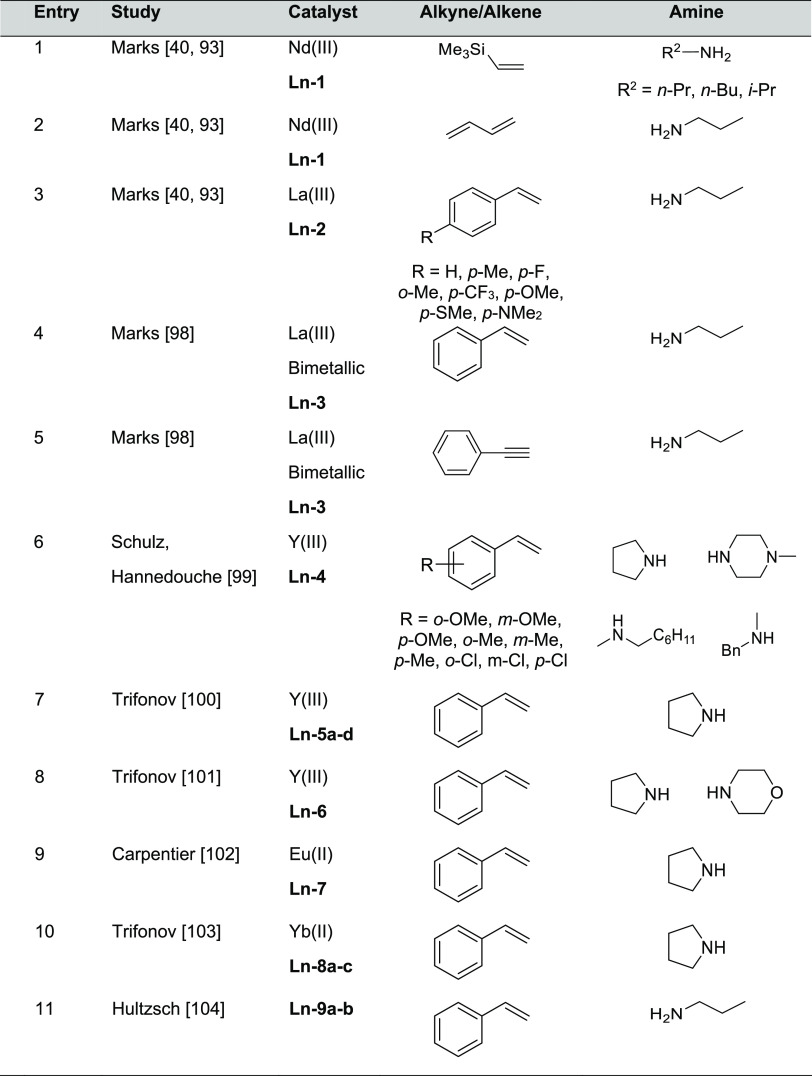
Experimentally Characterized Organolanthanide-Catalyzed Intermolecular Hydroaminations with Anti-Markovnikov Regioselectivity

**Scheme 21:**
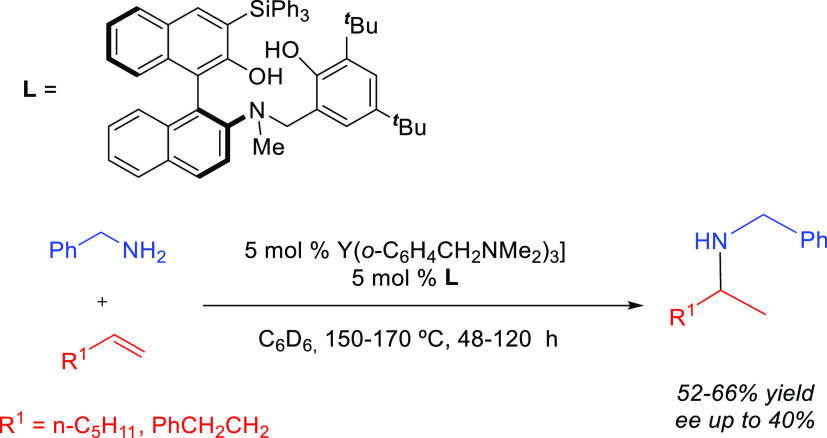
Intermolecular Hydroamination of Terminal Alkenes Catalyzed by Y-Based Aminodiolate Complexes^97^

Anti-Markovnikov regioselectivity was already found in the seminal work by Marks^93^ in 1996. Organolanthanide complexes with diverse structures, such as Me_2_SiCp_2_^′′^NdCH(SiMe_3_)_2_ (Cp′′ = η^5^-Me_4_C_5_) (**Ln-1)** and Cp_2_^′^LaCH(SiMe_3_)_2_ (Cp′ = η^5^-Me_5_C_5_) (**Ln-2)**, have been used as efficient precatalysts (7.5 mol % cat, C_6_H_6_, 60 °C, 3 days) for the regioselective intermolecular hydroamination of alkenes RCH=CH_2_ (R = SiMe_3_, CH_3_CH_2_CH_2_), butadiene, and vinylarenes (ArCH=CH_2_) using primary amines R′′NH_2_ (Table 3, entries 1–3). Interestingly, the intermolecular vinylarene hydroamination proceeds with excellent anti-Markovnikov regioselectivity. The reaction with butadiene affords only 1,4-addition product, implying that *N*-addition to the less substituted carbon atom (anti-Markovnikov addition) is clearly favored. The reaction with the terminal alkene Me_3_Si-CH=CH_2_ gives only the anti-Markovnikov addition. However, for 1-pentene (R = CH_3_(CH_2_)_2_) the Markovnikov addition product is observed, demonstrating the influence of the C–C multiple bond substituent in the regioselectivity.^40^ The corresponding Markovnikov addition products were also formed as the major products in the intermolecular hydroamination of aniline with terminal alkenes RCH=CH_2_ (R = CH_3_(CH_2_)_*n*_, *n* = 4, 5) promoted by Ln(OTf)_3_ complexes. In the case of allylbenzene, the Markovnikov product was isolated as the unique isomer.^96^ Similarly, the addition of benzylamine to 1-heptene and 4-phenyl-1-butene catalyzed by aminodiolate yttrium and lutetium complexes afforded exclusively the Markovnikov addition products in moderate yields at high temperatures (over 150 °C) and long reaction times (48–120 h) (Scheme 21).^97^

In a subsequent work, Marks et al. investigated hydroamination processes involving the cooperative effects of two Ln(III) metallic centers.^98^ Using the phenylene-bridged bimetallic organolanthanide complex *p*-bis{Cp′′La[N(SiHMe_2_)_2_]_2_}C_6_H_4_ (**Ln-3**), analogous anti-Markovnikov products for styrene and phenylpropyne were obtained (3–5 mol % precatalyst, C_6_H_6_, 25–90 °C) (Table 3, entries 4–5).

**Scheme 22:**
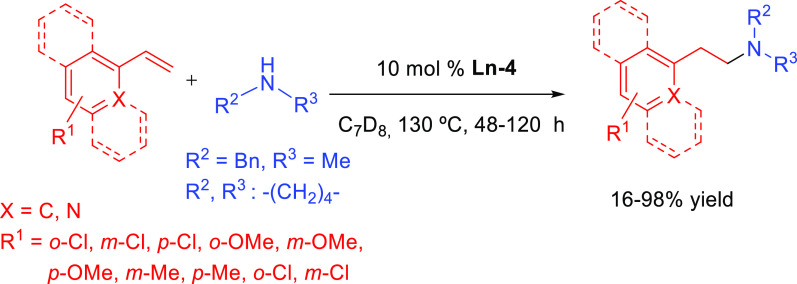
Yttrium-Catalyzed Intermolecular Anti-Markovnikov Hydroamination Reaction of Aromatic Alkenes with *N-*Methylbenzylamine or Pyrrolidine^99^

Schulz and Hannedouche extended the anti-Markovnikov selectivity to secondary amines (Table 3, entry 6). They demonstrated that binaphthylamido alkyl yttrium complexes **Ln-4** (1–10 mol %, C_7_D_8_, 25–130 °C, 5–48 h) facilitated the anti-Markovnikov addition between a wide variety of styrene derivatives and 2-vinylpiridine with secondary amines (yields in the range of 16–98% from 27 examples, Scheme 22).^99^ The authors also reported a tandem intermolecular anti-Markovnikov-enantioselective intramolecular hydroamination reaction with moderate enantioselectivity (48% ee). Alternatively, Trifonov’s group has described several yttrium(III) complexes capable to perform intermolecular hydroamination of styrene with secondary amines (2 mol %, neat, 70 °C, 3 days). Neutral and anionic yttrium alkyl complexes coordinated by a dianionic ene-diamido ligand (**Ln-5a** and **Ln-5b**, respectively), the dimeric hydride (**Ln-5c**), and a bisalkyl complex with a bulky amido-imino ligand (**Ln-5d**) exhibited anti-Markovnikov regioselectivity in the intermolecular hydroamination of styrene and pyrrolidine (Table 3, entry 7).^100^ Yttrium complex stabilized by a tridentate monoanionic amidopyridinate ligand (**Ln-6**) proved to be an efficient catalyst (2 mol %, neat, 70 °C, 2 days) for the intermolecular hydroamination of styrene with pyrrolidine and morpholine (Table 3, entry 8).^101^

Carpentier and Sarazin^102^ and then together with Trifonov^103^ succeeded in preparing lanthanide(II) complexes that did not undergo oxidation under catalytic conditions and acted as precatalysts for intermolecular hydroamination reactions (Table 3, entries 9, 10). The Eu(II) amide coordinated by iminoanilide ligand (**Ln-7**) catalyzes the addition of pyrrolidine to styrene in good to high yields in short reaction times (1–5 mol %, C_6_D_6_, 25–60 °C, 4–60 min).^102^ The same reaction is catalyzed by Yb(II)-amido complexes with bi- and tridentate amidinate and carbozyl ligands (1–2 mol % **Ln-8a–c**, neat, 60 °C, 0.25–60 h), but longer reaction times were required.^103^ Both the Eu(II) and Yb(II) complexes display exclusive anti-Markovnikov regiospecificity.

Hultzsch and co-workers developed La-based catalysts for hydroamination but employing binaphtholate-based ligands instead of cyclopentadienyl-based ligands known in the literature: catalysts **Ln-9a** and **Ln-9b** in Figure 5. Despite these catalysts being active for anti-Markovnikov aminocyclization of aminoalkenes (ratio [cat.]/[subs.] between 1 and 5, C_6_D_6_, 22–80 °C, 0.06–190 h), the later catalyst showed activity in the intermolecular addition of *n*-propylamine to styrene.^104^

#### 3.3.2 Mechanistic Considerations and Factors Governing the Regioselectivity

As shown in Figure 5, organolanthanide catalysts displaying anti-Markovnikov regioselectivity in intermolecular hydroaminations embrace a wide range of ligands, suggesting a very limited influence of the lanthanide ligands in the regioselectivity. A similar comment can be made when referring to the metal. In this regard, metal effects have been analyzed for the reaction between the internal alkyne 1-trimethylsilylpropyne and *n*-propylamine. The rate of the reaction decreases with shorter Ln^3+^ ionic radii (La^3+^ = 1.160 Å, Lu^3+^ = 0.977 Å).^105^ The reported turnover frequencies (TOFs) were between 14 and < 0.01 h^–1^ for Me_2_SiCp_2_^′′^NdCH(SiMe_3_)_2_ and Cp*_2_SmCH(SiMe_3_)_2_ at 60 °C, respectively.^40^ This behavior shows the evidence of steric constraints in the transition state, which is found to be common in organolanthanide-catalyzed reactions in which alkene insertion into a Ln–C or Ln–N bond is the rate-limiting step.^15^ In contrast, the nature of the C–C bond substituents has a major effect. As can be seen from Table 3, in most of the organolanthanide-catalyzed intermolecular hydroaminations with anti-Markovnikov regioselectivity the unsaturated hydrocarbon substrates are vinyl arenes. With aliphatic-substituted alkenes the reaction proceeds usually with Markovnikov selectivity.

**Figure 6:**
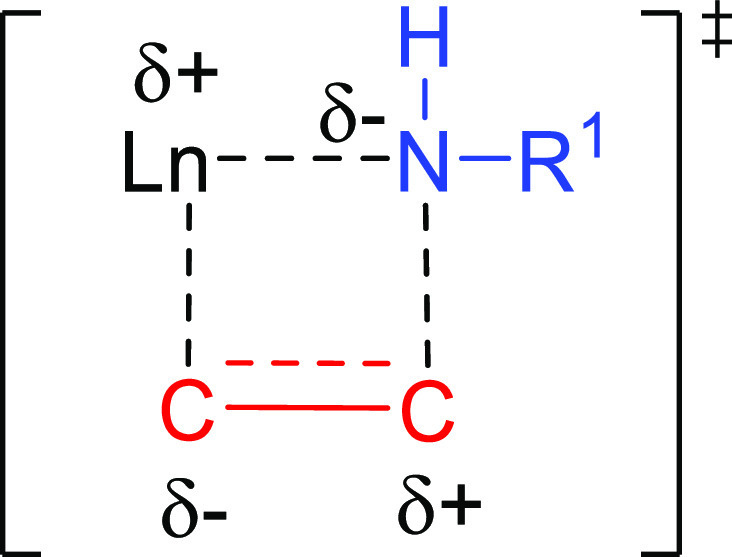
Charge distribution in the insertion transition state of an organolanthanide-catalyzed intermolecular hydroamination.

Regioselectivity in the lanthanide-like mechanism is decided at the C–C insertion step (see Scheme 2 in section 2.1.1). This step occurs through a charge polarized four-membered ring transition state (Figure 6) for which two different orientations are possible with a nonsymmetrically substituted alkene or alkyne.

**Figure 7:**
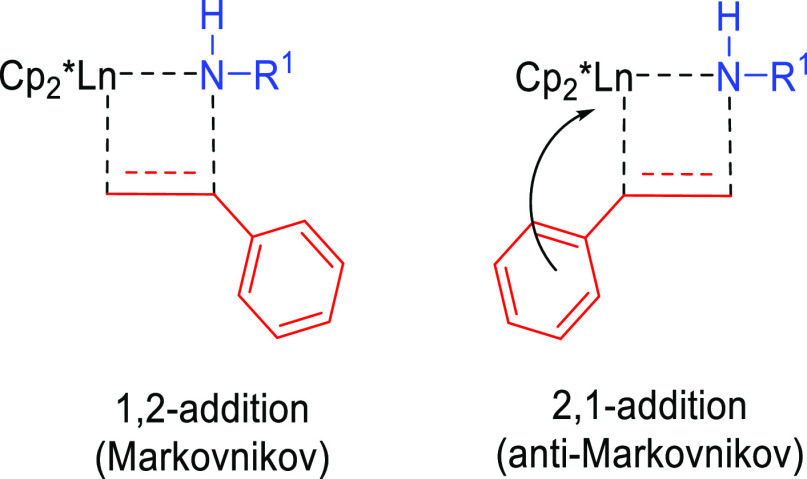
Aryl-directing interaction in organolanthanide-catalyzed hydroamination of vinyl arenes.

Secondary attractive or repulsive (steric) interactions can favor one orientation over the other. In this way, the observed anti-Markovnikov regioselectivity exhibited by vinyl arenes has been attributed to aryl-directing interactions between the arene π moiety and the electrophilic lanthanide center.^34^ Furthermore, attractive metal–arene interactions along with resonance stabilization of the benzyl carbanion can favor the 2,1-addition (anti-Markovnikov) over the 1,2-addition (Markovnikov) (Figure 7).

**Scheme 23:**
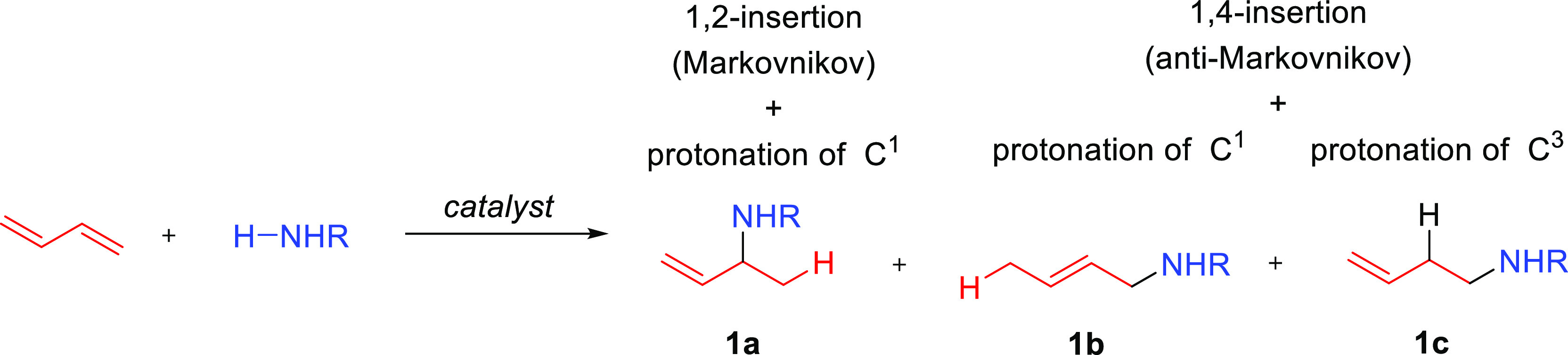
Possible Products in the Hydroamination of 1,3-Butadiene with Primary Amines

Tobisch performed a thorough DFT study (with the BP86 functional) of the intermolecular hydroamination of 1,3-butadiene with *n*-propylamine mediated by the Me_2_SiCp_2_^′′^NdCH(SiMe_3_)_2_ precatalyst (**Ln-1**).^106^ This study not only accounts for the experimental observations of this reaction^40,93^ but also supplies a deep insight into the general mechanistic features of organolanthanide-mediated intermolecular hydroaminations. Assuming the general insertion/protonolysis mechanism for organolanthanide-catalyzed hydroaminations depicted in Scheme 2, three different aminoalkene products can be formed (**1a–c**, Scheme 23). Insertion can proceed along the alternative 1,2- (Markovnikov) and 1,4-pathways (anti-Markovnikov). In the later, the ensuing protonolysis can take place at the C^1^ or C^3^ positions. The intermolecular hydroamination of 1,3-butadiene and *n*-propylamine by [Me_2_SiCp_2_^′′^NdCH(SiMe_3_)_2_] proceeds with 1,4-regioselectivity, and **1b** is the amino alkene that is exclusively formed in high yields (90%).^40^

Initially, precatalyst **Ln-1** is activated through protonolysis by amine substrate, which affords the amido–lanthanide complex, with release of the CH_2_(SiMe_3_)_2_ hydrocarbyl ligand. This activation step has a low Gibbs energy barrier (12.5 kcal mol^–1^) and is highly exergonic (−20.9 kcal mol^–1^). The amido-Nd species is coordinatively unsaturated, and it can coordinate amine substrate that is in excess. The amido–amine complex is the predominant species under actual reaction conditions and represents the catalyst’s resting state.

**Figure 8:**
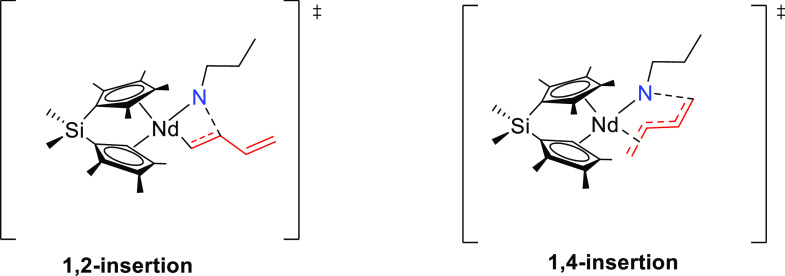
Transition states for the 1,2- and 1,4-insertions of butadiene into the Ln–N bond.^106^

Starting from the resting state amido–amine complex, the amine must first dissociate the catalyst to enable the coordination of 1,3-diene. Butadiene insertion takes place through a 4-membered transition state with a square-planar disposition of the Nd–N and the inserting C=C moiety. The regioselectivity of the insertion is decided at this C–N bond forming step, which determines the formation of linear or branched aminoalkene products. Both regioisomeric 1,4- and 1,2-insertion paths share the four-membered transition state structure, but while in the 1,4-transition state the butenyl-Nd coordination adopts a η^3^-π coordination mode, in the 1,2-transition state it is of η^1^-σ type (Figure 8). The stronger η^3^–π interaction, commonly labeled as the π–allyl interaction and known for stabilizing the charge, confers stability at the 1,4-transition state over the 1,2-transition state. The 1,4-pathway, leading to the anti-Markovnikov amine addition, is favored, both thermodynamically (ΔΔ*G* > 13 kcal mol^–1^) and kinetically (ΔΔ*G*^‡^ > 7 kcal mol^–1^) relative to the 1,2-Markovnikov addition. C–N bond formation takes place with complete regioselectivity along the 1,4-pathway. Overall, butadiene insertion is predicted to be almost thermoneutral in terms of Gibbs energy and must overcome an activation barrier of about 14 kcal mol^–1^. As expected for a bimolecular step, a large negative activation entropy (Δ*S*^‡^ = –30 e.u.) is associated with the insertion step.

**Figure 9:**
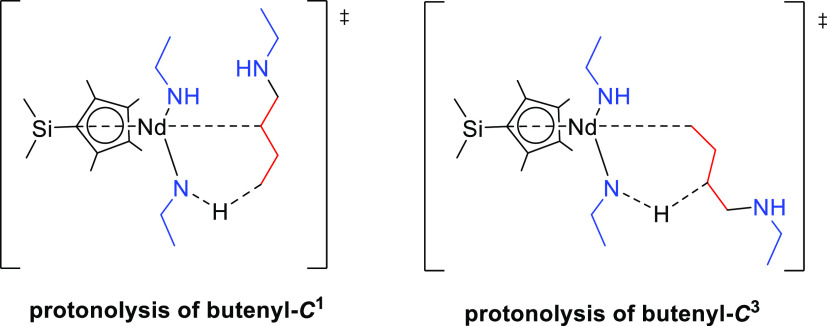
Transition states for protonolysis of the η^3^-butenyl–Nd complex by amine substrate, at butenyl-C^1^ and butenyl-C^3^ positions.^106^

After the insertion step, protonolysis by one molecule of amine substrate of the η^3^-butenyl intermediate affords an aminoalkene–amido–Nd complex, from which the aminoalkene product is released in a facile substitution by an incoming amine, regenerating the amido complex and initiating a new catalytic cycle. Protonolysis takes place via a σ-bond metathesis type transition state, with synchronous N–H bond cleavage and C–H bond formation in the vicinity of the Nd center but can occur at either butenyl–C^1^ or butenyl–C^3^ positions (Figure 9).

Among the two regioisomeric pathways, proton transfer to the butenyl C^1^ carbon atom is more feasible kinetically (ΔΔ*G*^‡^ = 5.4 kcal mol^–1^) and is also more favorable thermodynamically (ΔΔ*G* = 9.0 kcal mol^–1^), which has its primary origin in the coordinative assistance of the chelating amine tether functionality.^106^ These results rationalize the almost unique regioselectivity of the proton-transfer process.

### 3.4 Actinides

Organoactinides and actinide coordination complexes have found application as homogeneous catalysts for a wide diversity of organic transformations.^107^ Since the seminal work of Zalkin and Raymond describing the synthesis of uranocene, [(η^8^-C_8_H_8_)_2_U] in 1969,^108^ the use of coordination complexes of actinides has experienced a blossoming in organic synthesis. Actinide (**An**) complexes have been proven to be catalytically active by a mechanism in which a C–C unsaturated bond inserts into an An–N bond by means of a four-membered transition state.^35,109^ For this reason, they have potential as hydroamination catalysts and a number of organoactinide complexes have been investigated as catalyst in the intermolecular hydroamination of terminal alkynes with primary aliphatic and aromatic amines.^32^ In some cases, they display anti-Markovnikov regioselectivity.

#### 3.4.1 Actinide-Based Catalysts

**Figure 10:**
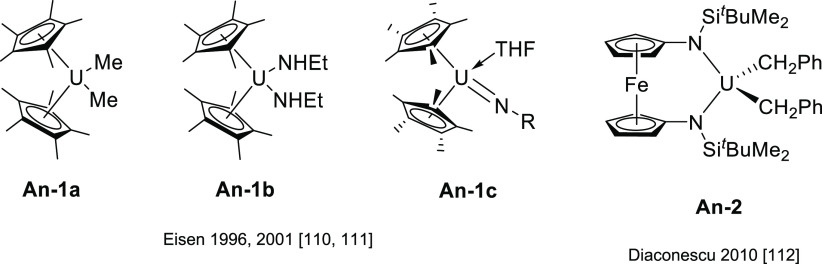
Organoactinide catalysts featuring anti-Markovnikov regioselectivity.

Structures of several actinide hydroamination catalysts featuring anti-Markovnikov regioselectivity (**An-*x***) are shown in Figure 10. Details of the reactions are given in Table 4. Secondary amines are generally unreactive in hydroamination processes, and the actinide-catalyzed reaction is believed to proceed via a metal-imido species similar to that of group 4 metal complexes (see Imido Mechanism, section 2.1.2 and Scheme 3). Contrary to what happen with lanthanides (Table 3), anti-Markovnikov selectivity is also found with alkyl-substituted alkynes (Table 4). No hydroamination products were formed when internal alkynes were used.

**Scheme 24:**
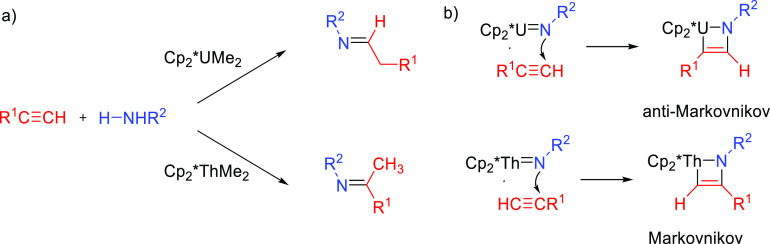
(a) Reactivity of Uranium and Thorium Complexes with Alkynes; (b) Different Activation Modes for Uranium and Thorium Complexes

Organoactinide complexes Cp*_2_UR_2_ (R = Me (**An-1a**), NHR (**An-1b**)) and Cp*_2_U(NR)(THF) (**An-1c**) catalyze the intermolecular hydroamination of terminal aliphatic and aromatic alkynes with primary aliphatic amines to yield the corresponding imino compounds in high yields.^110,111^ The reaction leads exclusively to the anti-Markovnikov adduct (Table 4, entry 1) under the reaction conditions (THF or C_6_H_6_, 60 °C). Interestingly, changing uranium by thorium causes a change in the regioselectivity, from anti-Markovnikov to Markovnikov (Scheme 24a), except for (SiMe_3_)—C≡C—H.

**Table 4:**
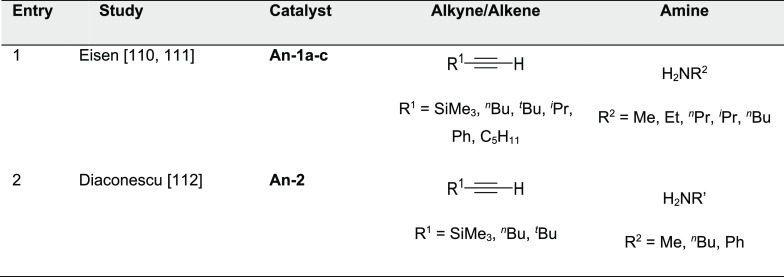
Experimentally Characterized Organoactinide-Catalyzed Intermolecular Hydroaminations with Anti-Markovnikov Regioselectivity

Diaconescu and co-workers have reported the intermolecular hydroamination of alkynes mediated by a uranium dibenzyl precatalyst bridged by a diamine ferrocene ligand (**An-2**, Figure 10). This complex catalyzes (10 mol %, C_6_D_6_, 70 °C) the addition of both aromatic and aliphatic amines with anti-Markovnikov regioselectivity and high conversions (up to 97%) for aromatic amines but low to moderate in the case of aliphatic amines (18–70%) with longer reaction times (Table 4, entry 2).^112^

#### 3.4.2 Mechanistic Considerations and Factors Governing the Regioselectivity

**Scheme 25:**
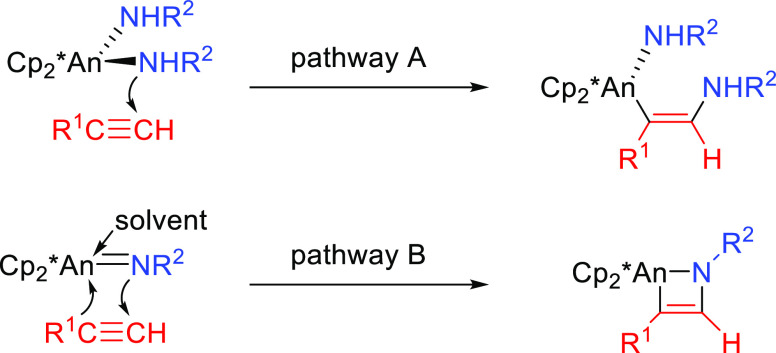
Metal-Amido and Metal-Imido Pathways for the Intermolecular Hydroamination of Terminal Alkynes Mediated by Organoactinide Complexes. Anti-Markovnikov addition is assumed.

The most controversial mechanistic issue in the hydroamination reactions catalyzed by actinides has been whether they take place via the insertion of an alkyne into a metal–amido bond (“Lanthanide-like” Mechanism, section 2.1.1; Scheme 25, pathway A)^113^ or by the insertion of an alkyne into a metal–imido bond, as also observed and discussed for group 4 transition metal complexes (Imido Mechanism, section 2.1.2) (Scheme 25, pathway B).^109^

**Figure 11:**
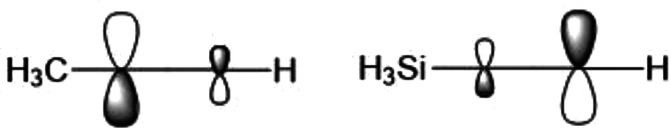
Polarization of the π* orbital in R—C≡C—H terminal alkynes: (a, left) R = Me; (b, right) R = SiMe_3_.

Regarding intermolecular reactions, the lack of hydroamination reaction with secondary amines, as well as the lack of alkyne participation on the kinetic hydroamination rate, suggested that the metal-imido pathway is involved. Extensive kinetic investigations by Eisen and Diaconescu agree with a terminal-imido actinide complex as the likely catalytically active species.^111,112^ The zero-order kinetics with respect the alkyne found is consistent with a fast insertion of different alkynes, with indistinguishable rates, in a highly unsaturated imido complex. Thus, the accepted mechanism for intermolecular hydroaminations promoted by organoactinides involves the formation of an actinide imido intermediate, which then undergoes a [2 + 2] cycloaddition with the alkyne to generate an amido–vinyl actinide complex. This complex is protonated by an incoming amine and releases the product (enamine), that generally isomerizes to the imine. In this mechanistic scenario, the change of the addition product from anti-Markovnikov (uranium) to Markovnikov (thorium) (Scheme 24) has been attributed to a metal effect, related with the electronic differences in their imido complexes.^111^ With (SiMe_3_)—C=C—H the same anti-Markovnikov regioselectivity is obtained by both thorium and uranium catalysts. The similar stereochemistry in the addition of (SiMe_3_)—C=C—H to both organoactinides has been explained by an electronic effect due to the SiMe_3_ alkyne substituent, which polarizes the alkyne π* orbitals in an opposite direction to that of a Me substituent (Figure 11).^114^ In the SiMe_3_-substituted alkyne the contribution of the terminal carbon in the π* orbital is larger, making it more suitable for the nucleophile addition.

A different catalytic cycle has been proposed for intramolecular hydroamination/cyclization of aminoalkenes and aminoalkynes, for which experimental mechanistic investigations support An-N(H)R intermediates instead of An = NR intermediates.^112,113^ A computational study of the intramolecular hydroamination/cyclization of (4*E*,6)-heptadienylamine with thorium CGC complexes (CGC = [Me_2_Si(η^5^-Me_4_C_5_)(*^t^*BuN)]^2-^) indicated that the amido mechanism is favored over the imido pathway.^115^

### 3.5 Early Transition Metals

Early transition metallic complexes have also been efficiently used in hydroamination reactions. Among early transition metals, titanium and zirconium are the most common for this type of reactions and offer the advantage of being more economically accessible than late transition metals or rare earth metal complexes.^116^ In this regard, group 4 metals, in particular, titanium and zirconium have natural abundances of 6320 and 162 ppm, respectively; while hafnium has only an abundance of 3 ppm. Regarding the tolerance of the functional groups by early transition metals, there was a preconception of low tolerance, although it was shown that it was not substantiated.^2^

#### 3.5.1 Titanium

Titanium catalysts have attracted wide interest due to their low toxicity, earth abundance, and low cost, as titanium is among the ten most abundant elements in the Earth’s crust. In the last decades, titanium catalysts have emerged as promising candidates in hydroamination reactions facilitating high yields and elevated selectivities.

##### 3.5.1.1 Titanium-Based Catalysts

**Figure 12:**
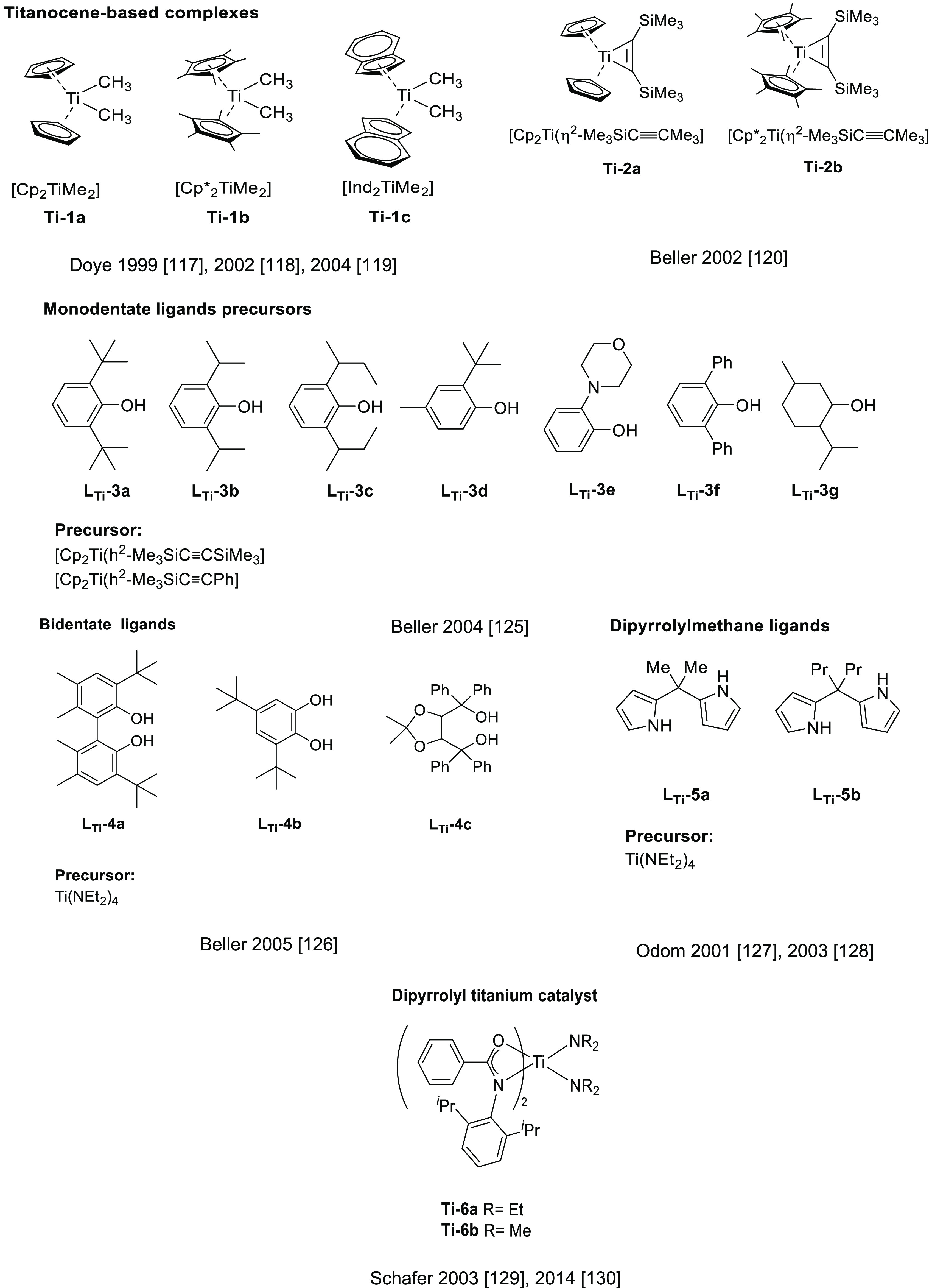
Titanium catalysts and ligands featuring anti-Markovnikov regioselectivity.

Titanium catalysts active in anti-Markovnikov hydroaminations are gathered in Figure 12.

The first catalytic intermolecular hydroamination of internal alkynes in the presence of a titanium complex was reported by Doye and co-workers in 1999.^117^ In this seminal work, aryl- and alkylamines were coupled with diphenylacetylene at 100 °C in toluene with 3 mol % of readily available dimethyltitanocene, [Cp_2_TiMe_2_] (**Ti-1a,**Table 5, Entry 1), as catalyst. For unsymmetrically substituted alkynes, the hydroamination afforded exclusively the anti-Markovnikov products.

**Table 5:**
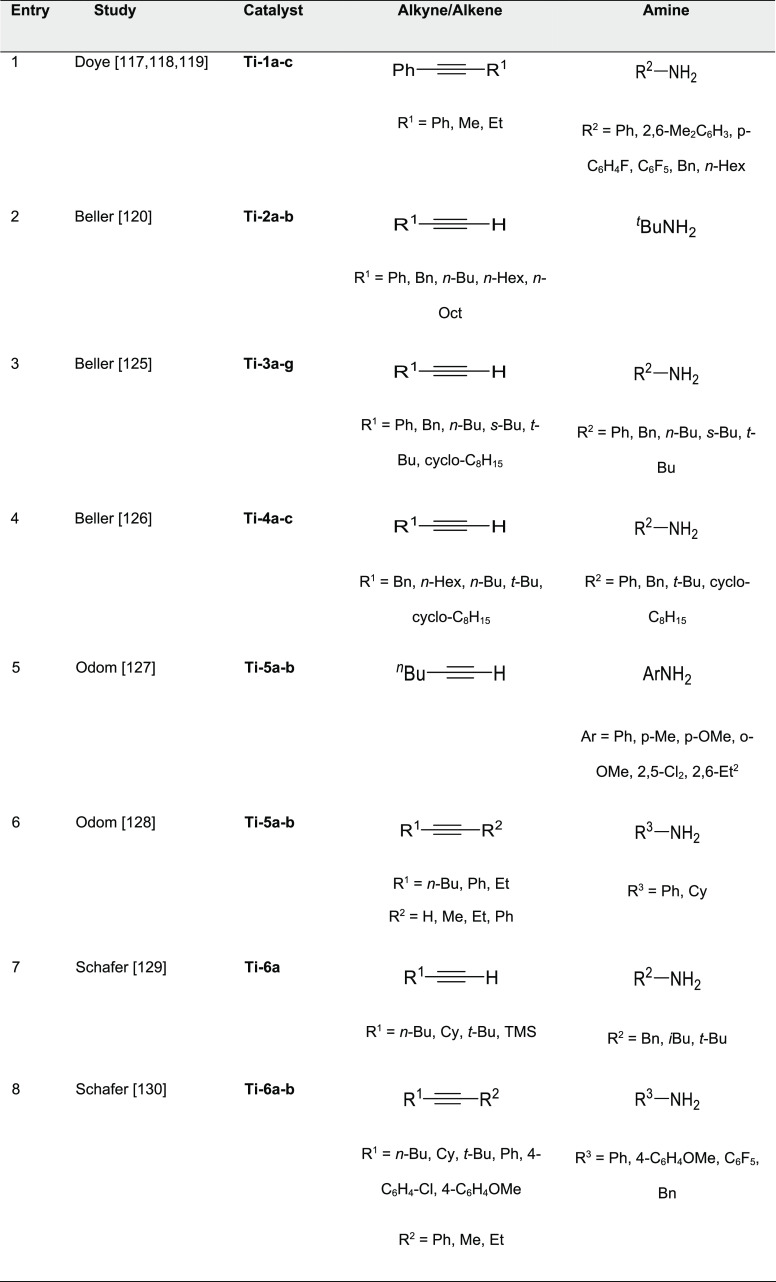
Experimentally Characterized Titanium-Catalyzed Intermolecular Hydroaminations with Anti-Markovnikov Regioselectivity

**Scheme 26:**
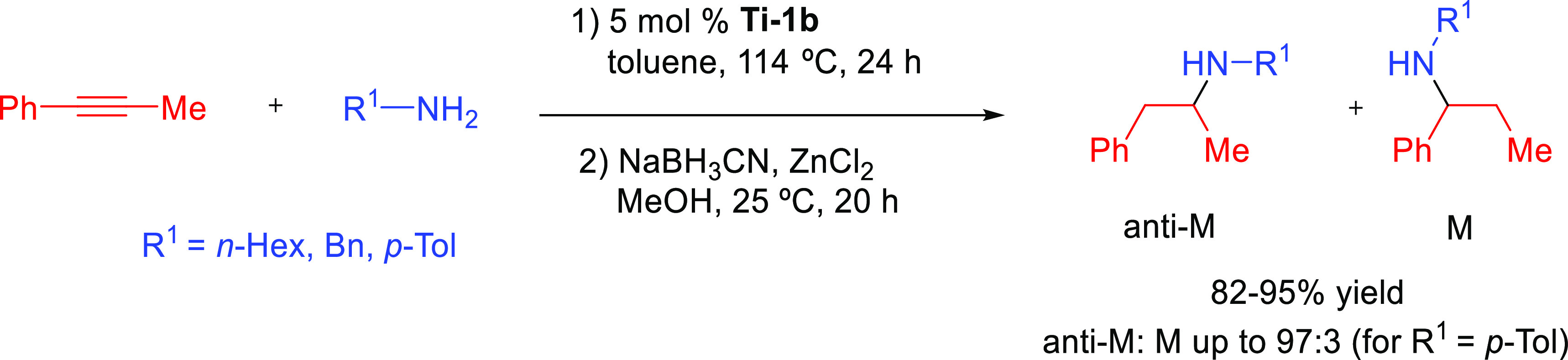
Intermolecular Hydroamination of Internal Alkynes in the Presence of a Titanium Complex Described by the Doye Group^118^

In a later study, the authors showed that an increase of the steric bulk around the cyclopentadienyl ligands afforded in good yields the hydroamination of alkylamines with low steric bulkiness.^118^ In this regard, [Cp*_2_TiMe_2_] (**Ti-1b**) was an active catalyst for the intermolecular addition of various sterically less hindered *N*-alkyl- and benzylamines to unsymmetrical internal alkynes in high yields but with low regioselectivities. Optimal results for catalyst **Ti-1b** were obtained when employing the sterically more demanding amine 4-methylaniline (*p*-Tol-NH_2_), which afforded the anti-Markovnikov product in a 92% yield and a selectivity of 97:3 (Scheme 26). A comparative experiment employing catalyst **Ti-1a** gave similar results (95% yield for the anti-Markovnikov product and selectivity 98:2), indicating that the structure of the amine, and not the Cp*-ligands, is responsible for the low regioselectivity of **Ti-1b** with sterically less demanding *n-*alkyl- and benzylamines. In a next step, the obtained imine products were reduced with Zn-modified NaBH_3_CN in methanol at 25 °C to give the desired amine. In 2004, a broader scope of reactivity could be achieved by replacement of the cyclopentadienyl ligands with indenyl ligands, [Ind_2_TiMe_2_] (Ind = η^5^-indenyl, **Ti-1c**).^119^ This catalyst was highly active (5.0 mol %, toluene, 105 °C, 3–48 h) in the intermolecular hydroamination of a diversity of amines, including primary aryl-, *tert*-alkyl-, *sec*-alkyl-, and *n*-alkylamines, with both internal and terminal alkynes (Table 5, Entry 1); it affords the desired product in high yields and modest to excellent regioselectivities, favoring the formation of the anti-Markovnikov product, whereas alkylalkynes reacted with arylamines to give preferentially the Markovnikov regioisomer (Scheme 26). In particular, 1-phenyl-2-alkylalkynes with small alkyl substituents are hydroaminated much more regioselectively than substrates with bulky substituents. Furthemore, the reaction between the bulky 4-methylaniline and 1-phenyl-2-alkylalkynes gave the desired secondary amine with an excellent anti-Markovnikov selectivity (ratio aM:M 49:1, entry 1, Table 5), while the regioselectivity for the addition of the sterically less demanding *n*-propylamine was only 3:1. A kinetic study showed faster reaction rates for [Ind_2_TiMe_2_] (**Ti-1c**) when compared to [Cp_2_TiMe_2_] (**Ti-1a**), which was attributed to a reversible dimerization of the catalytically active species for **Ti-1a**, which does not occur for **Ti-1c** imido complex.

**Scheme 27:**
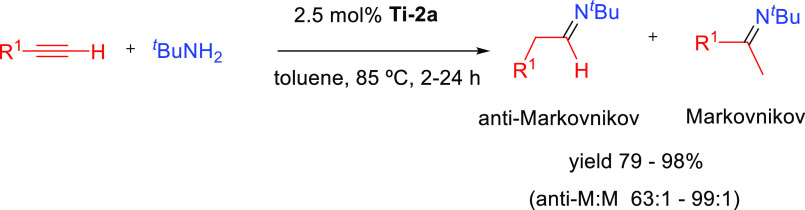
Titanocene-Catalyzed Hydroamination of Terminal Aliphatic Alkynes with Anti-Markovnikov Selectivity Reported by the Beller Group^120^

The group of Beller has been very active in the study and design of titanium catalysts for the anti-Markovnikov hydroamination of alkynes in the past decades, reporting the use of titanocene-based catalysts as well as aryloxo and alkyloxo ligands with excellent regioselectivities (Figure 12). In 2002, this group reported the pioneering use of titanocene catalysts (2.5 mol % Ti catalyst, toluene, 85 °C, 24 h) in the hydroamination of terminal aliphatic alkynes with anti-Markovnikov selectivity (Scheme 27).^120^

In this study, Beller and co-workers employed a titanocene–alkyne complex of the type [Cp_2_Ti(η^2^-Me_3_SiC≡CMe_3_] (**Ti-2a**) for the hydroamination of terminal aliphatic alkynes with aliphatic amines in high yields (79–98%) and with high regioselectivity (63:1 – 99:1) (Scheme 27). Excellent anti-Markovnikov selectivity was observed when using terminal aliphatic alkynes with bulky alkylamines, such as *tert*-butylamine (Table 5, Entry 2); however, when using less bulky amines, a decrease in the anti-Markovnikov selectivity was observed. A reversal of regioselectivity was observed for aromatic amines, which yielded the Markovnikov product with moderate selectivity (3:1).

**Scheme 28:**
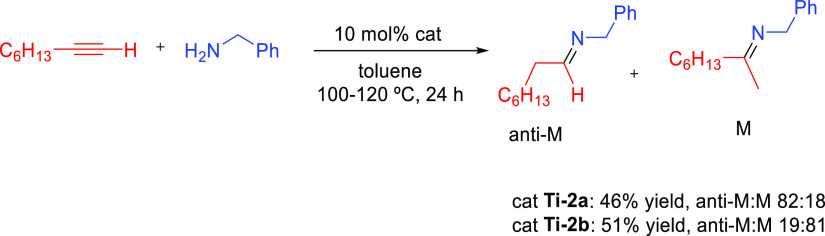
Regioselectivity Using Titanocene Catalysts Reported by the Beller Group^122^

Dual stereo- or regiocontrolled reactions are of utmost importance in synthetic organic chemistry, as they allow the formation of enantiomers or regioisomers by tuning the catalyst structure or by modification of the reaction conditions.^121^ In a continuation of a previous work, Beller and co-workers reported on the tuning of the regioselectivity of the hydroamination product for aliphatic amines by using a more sterically bulky complex derived from Rosenthal’s catalyst.^122^ In this regard, as shown in their previous work, anti-Markovnikov imines were regioselectively obtained in the presence of titanocene–alkyne complex [Cp_2_Ti(η^2^-Me_3_SiC≡CMe_3_] (**Ti-2a**, 2.5 mol %, toluene, 85 °C, 24 h), even for nonhindered amines, which were obtained at higher temperatures (100–120 °C) in lower yields that hindered amines. On the other hand, catalyst [(η^5^-Cp*)_2_Ti(η^2^-Me_3_SiC≡CMe_3_] (**Ti-2b**) favored the Markovnikov product (Scheme 28). The scope of the reaction revealed that an increase in the steric bulk of aliphatic alkynes increased the regioselectivity of the anti-Markovnikov products, whereas the selectivity of the Markovnikov isomer could be favored by increasing the steric bulk in aromatic amines. Electronic effects were also important, as electron donating substituents favored the anti-Markovnikov products.

**Scheme 29:**
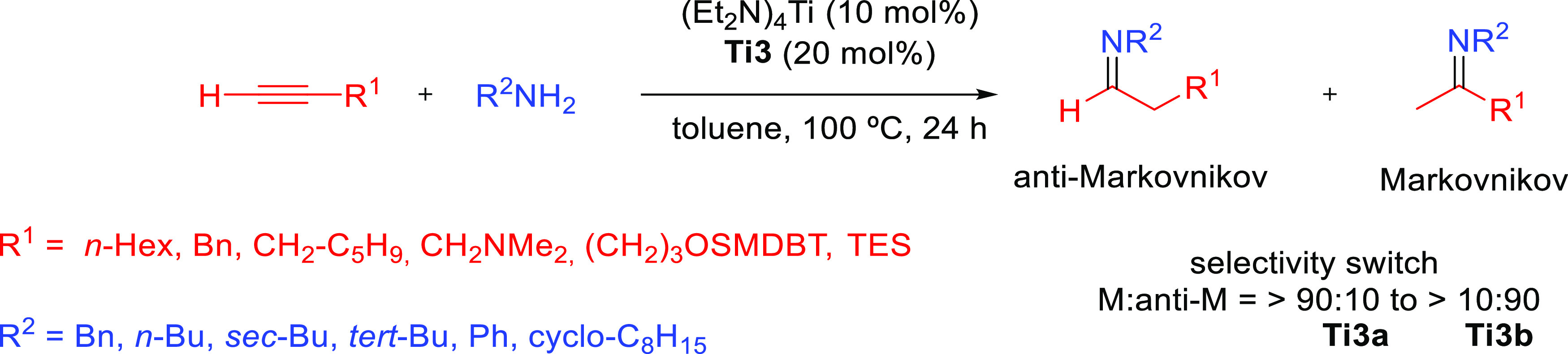
Selectivity Switch with Aryloxo Ligands Reported by the Beller Group^125^

Another family of titanium-based catalysts, which have been efficiently used in hydroamination reactions, are those containing aryloxo and alkoxo ligands (Figure 12).^123^ These catalysts offer an easy handling, higher air and moisture stability, and a wider ligand variety than those of titanocene precatalysts. In this regard, Beller and co-workers reported the use of aryloxotitanium complexes (5 mol %, toluene, 100 °C, 24 h) in the intermolecular hydroamination of terminal alkynes.^124^ A notable inversion in selectivity from the Markovnikov to the anti-Markovnikov product was observed by slightly modifying the titanium ligand (Scheme 29).^125^ In this regard, the use of bulky ligand 2,6-di-*tert*-butyl-4-methylphenol (**Ti-3a**) gave the hydroamination products in high yields and elevated Markovnikov selectivity, which could be reversed by using 2,6-diisopropylphenol (**Ti-3b**) as ligand (Table 5, Entry 3). Nevertheless, high reaction temperatures up to 140 °C were needed with these ligands.

In another work by Beller and co-workers in 2005, they described the use of monodentate and bidentate aryloxo derivatives as efficient ligands in the titanium-catalyzed intermolecular hydroamination of terminal alkynes (Figure 12).^126^ Titanium catalytic species were formed in situ from commercially available tetrakis(diethylamino)titanium, Ti(NEt_2_)_4_, and the corresponding aryloxo ligands in a 1:2 molar ratio (10 mol % Ti(NEt_2_)_4_, 20 mol % ligand, toluene, 100 °C, 24 h). In general, sterically hindered aryloxo ligands except ligand **Ti-3h** showed high activity in favor of the anti-Markovnikov imine. Only for aryloxo ligand **Ti-3a** and alkyloxo ligand **Ti-3g** could the regioselectivity be controlled by the amine. In this regard, the reaction of 1-octyne with benzylamine yielded preferentially the Markovnikov product, whereas the more steric *tert*-butylamine favored selectively the anti-Markovnikov isomer. All the other sterically hindered aryloxo ligands yielded the anti-Markovnikov independently of the used amine. The reaction in the presence of bidentate ligands (Table 5, entry 4) favored the formation of the Markovnikov products but in low yields (10–52%) and with lower selectivity than the monodentate aryloxo derivates.^125^

Odom and co-workers described that commercially available Ti(NMe_2_)_4_ and precatalysts formed with dipyrrolyl titanium exhibited moderate to high reactivity in the hydroamination of alkynes with aniline derivatives but showed low reactivity toward aliphatic amines.^127,128^ The use of Ti(NMe_2_)_4_ as a precatalyst (10 mol %, toluene, 70 °C) and high temperatures offered a high Markovnikov selectivity for the hydroamination of alkynes in long reaction times. On the other hand, using dipyrrolyl titanium catalysts **Ti-5a–b** (10 mol %), only the anti-Markovnikov product was formed under mild conditions for the reaction of cyclohexylamine with 1-hexyne, but with low regioselectivity (Table 5, Entry 5). The scope of hydroamination reactions with **Ti-5a** as catalyst was extended to cyclohexylamine using chlorobenzene as solvent (1:1 alkyne:amine ratio and 5–10 mol % **Ti-5a**), yielding the anti-Markovnikov product with high yields and moderate selectivity (up to 11:1); however, the Markovnikov product was obtained only in the case of reaction with 1-hexyne (Table 5, Entry 6).^128^

The group of Schafer has been quite productive in titanium-catalyzed anti-Markovnikov intermolecular hydroamination reactions in the past decades. In 2003, they reported a flexible diamido bis(amidate) Ti complex **Ti-6a** as a precatalyst (5 mol %, C_6_D_6_, 65 °C, 6–24 h) for the hydroamination of alkynes with primary alkyl amines (Figure 12) and tolerating sterically demanding substrates such as cyclohexyl- and *tert*-butylalkynes under longer reaction times (Table 5, Entry 7).^129^ This bis(amidate)titanium system, which could be characterized by its X-ray crystal structure, contains 2,6-diisopropylphenyl substituents, has *C*_2_ symmetry, and displays a *trans* orientation of the N atoms of the amidate ligands and a *cis* orientation of the amide ligands (Table 5, Entry 8). The scope of the reaction was extended by using precatalyst **Ti-6b**. With this bis(amidate)titanium catalyst (5 mol %, C_6_D_6_, 65 °C), both aliphatic and aromatic amines also reacted with terminal and internal alkynes having diverse functionalities, including halides, amides, esters, and protected amines and alcohols, and in most cases, the anti-Markovnikov products were exclusively obtained.^130^ This precatalyst has also been efficiently applied in the synthesis of *N*-heterocyclic compounds, such as primary and secondary amines,^131^ isoquinolines,^132^ chiral morpholines,^133^ and pyridines,^134^ among others.^21^ These studies revealed that bulky amidate ligands were necessary to achieve high reactivity toward hydroamination reactions.

##### 3.5.1.2 Mechanistic Considerations and Factors Governing the Regioselectivity

**Scheme 30:**
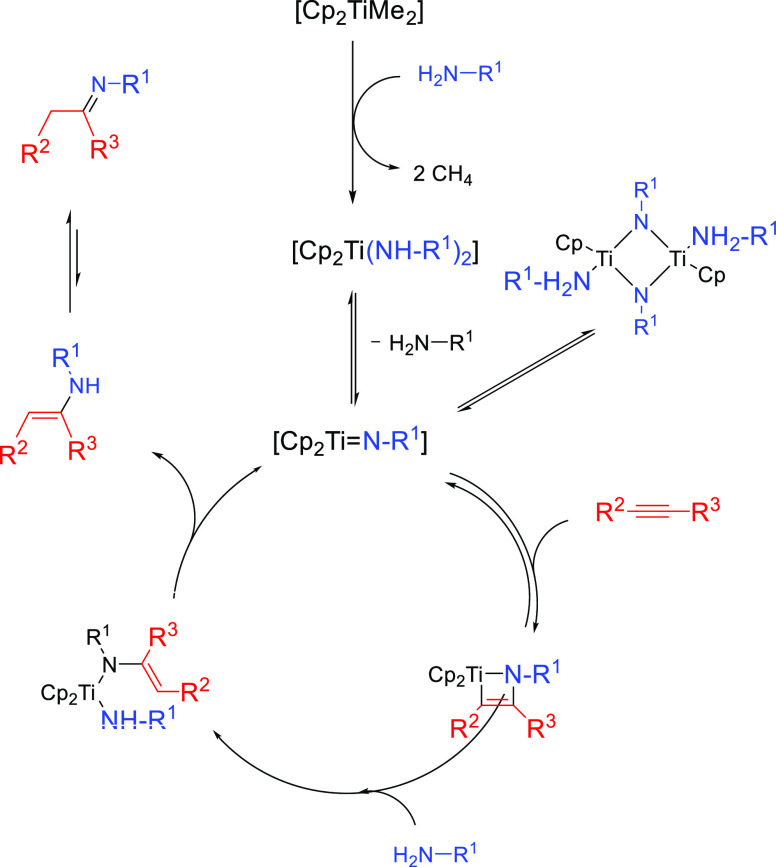
General Mechanism of the [Cp_2_TiMe_2_]-Catalyzed Hydroamination of Alkynes^135^

The most commonly accepted mechanism for Ti-catalyzed hydroamination reactions is the imido mechanism, also called the [2 + 2] cycloaddition mechanism, outlined in section 2.1.2 (Scheme 3). The general mechanism for the hydroamination of alkynes catalyzed by cyclopentanedienyl titanium complexes is depicted in Scheme 30. The catalytic cycle starts with the activation of the amine by reaction with the precatalyst [Cp_2_TiMe_2_], through the formation of an imido–titanium complex, which is the catalytically active species, as described by mechanistic investigations by Bergman et al.^135^ A reversible equilibrium exists between the catalytically active titanium imido complex and Ti-dimeric species, which causes a nonlinear effect between the catalyst concentration and the observed reaction rate, as described in a kinetic study by Pohlki and Doye.^136^ Next, a reversible [2 + 2] cycloaddition of the alkyne with the imido-titanium species yields an azatitanacyclobutene intermediate. This resulting azatitanacylobutene species is irreversibly protonated by an amine to form the bisamide compound, which is then cleaved into an enamine and regenerates the catalytically active species. Finally, the formed enamine is converted into the corresponding imine. DFT calculations (using the B3LYP functional) performed by Straub and Bergman strongly support the described mechanism for the [Cp_2_TiMe_2_]-catalyzed intermolecular hydroamination of alkynes.^137^ However, this mechanistic study used a symmetrical alkyne as a model system, circumventing the problem of regioselectivity.

Based on the mechanism proposed by Straub and Bergman, a more complete computational study was performed by Beller and co-workers using real titanocene complexes and propyne as a model for the terminal aliphatic alkyne.^122^ The difference of regioselectivity observed experimentally could not be rationalized by the difference in the activation energies for each reaction, as the energy difference was negligible (<0.3 kcal mol^–1^). The authors proposed that the difference in the relative stability of the imido–alkyne π complex could be responsible for the observed regioselectivity. The different stability of these π complexes was attributed to the natural charge distribution and structural parameters such as (i) the distance between the Ti=N double bond of the titanium imido complex and the C≡C triple bond of propyne and (ii) the orientation of propyne with respect to the titanium complex.

**Scheme 31:**
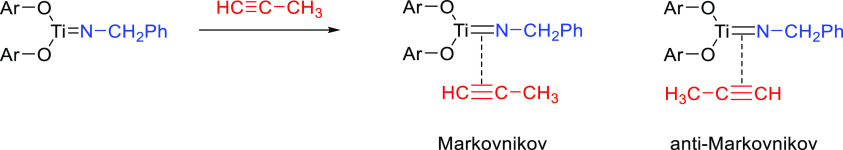
Aryloxo Titanium Complexes in Hydroamination of Terminal Alkynes

As described above, in the case of aryloxo complexes, the regioselectivity depends on the substituents of the aromatic rings of the aryloxo ligands and is determined by the different stabilities of the preformed π complexes between the aryloxo titanium–imido complex and the alkyne. As shown in Scheme 31, the (ArO)_2_Ti(=N—CH_2_Ph) complex can coordinate the attacking propyne in both Markovnikov and anti-Markovnikov orientations. DFT calculations at the B3LYP/LANL2DZ level of theory reported by Beller and co-workers showed that in the case of **Ti-3a** ligand, the Markovnikov π complex was 6.5 kcal mol^–1^ more stable than the corresponding anti-Markovnikov π complex, whereas for the **Ti-3b** ligand, the anti-Markovnikov π complex was found to be 1.2 kcal mol^–1^ more stable, in good agreement with the experimentally observed regioselectivity.^126^

**Figure 13:**
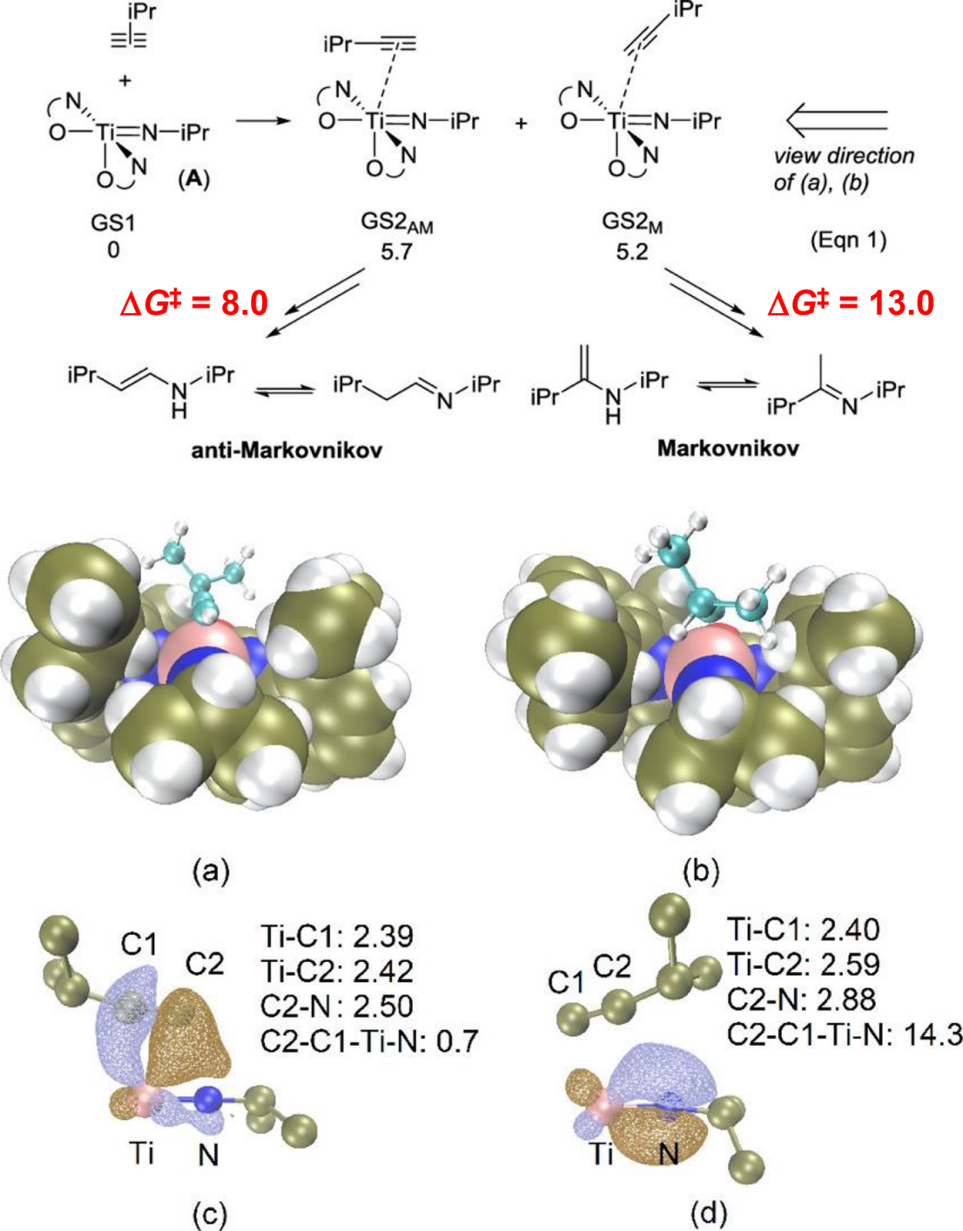
Alkyne molecule in the “pocket” of Ti-imido supported by two amido ligands in anti-Markovnikov (GS2_AM_, (a)) and Markovnikov (GS2_M_, (b)) intermediates. Their relative Gibbs energies (kcal mol^–1^) are also given. Localized Molecular Orbital picture of the Ti-imido to alkyne interaction in GS2_AM_ (c) and GS2_M_ (d). Adapted with permission from ACS Catal.2020, 10 ( (13), ), 7100–7111. Copyright 2020 American Chemical Society.^138^

The Schafer group reported diamido bis(amidate) Ti precatalysts with outstanding selectivity for anti-Markovnikov alkyne hydroamination (**Ti-6a**, Figure 12).^129^ Recently Hao and Schafer have published a thorough DFT study (PBE0-D3 functional with Def2-SVP basis set) aimed to get deeper insights into the mechanism of this reaction and the factors controlling the observed regioselectivity.^138^ The authors broke the catalytic cycle into four parts: (i) the precatalyst activation (formation of catalytically active Ti–imido complex), (ii) the [2 + 2] cycloaddition, (iii) the ring-opening protonolysis, and (iv) the regeneration of the Ti–imido complex (analogous to that presented in Scheme 30). The hypothesis of Beller and co-workers relating the reaction regioselectivity to the stability of the Ti–alkyne adduct^122^ cannot be applied in this system, as it would predict a product ratio of 1:2.4 favoring the Markovnikov product (this is the outcome when comparing the relative Gibbs energies of GS2_AM_ and GS2_M_ in Figure 13). However, looking at the [2 + 2] cycloaddition step, in which the C–N bond is formed, the anti-Markovnikov transition state is 5 kcal mol^–1^ lower than the Markovnikov one. A comprehensive analysis discloses electronic effects, and particularly the favored interaction between the Ti-imido π orbital and the alkyne π* orbital in the anti-Markovnikov approximation, as the main responsible factor for the regioselectivity. The better fitting of the alkyne into the vacant coordination site of the Ti metal center for the anti-Markovnikov addition is already apparent in GS2 intermediates (Figure 13). Overall, although the anti-Markovnikov pathway generally has a lower transition state energy compared to the alternative Markovnikov pathway, substrate dependent regioselectivity is obtained in the experimental systems due to small energy differences in the decisive transition state structures.^138^

#### 3.5.2 Zirconium

Cationic zirconium species are isovalent with group 3 and lanthanide metal complexes and can successfully catalyze hydroamination reactions.

##### 3.5.2.1 Zirconium-Based Catalysts

**Figure 14:**
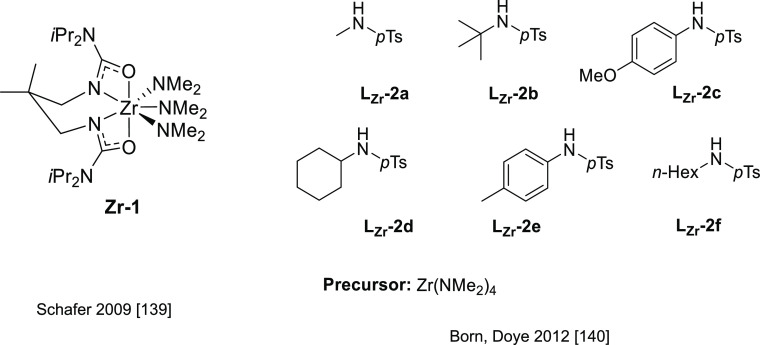
Zirconium-based catalysts and ligands for anti-Markovnikov hydroaminations.

The first example of the use of a catalytic zirconium complex in intermolecular hydroamination of alkynes was reported by Walsh, Baranger, and Bergman in 1992.^43^ In this preliminary work, the authors used a zirconocene catalyst (Cp_2_Zr(NHR)_2_) which is converted into a catalytically active zirconium–imido complex, but it only showed activity with the sterically demanding primary amine 2,6-dimethylaniline, failing to undergo hydroaminations with other amines as substrates. As also described for titanium-catalyzed hydroamination reactions, a reduction step to convert the enamine product into the desired amine is required. Since this seminal work, a few zirconium-based catalysts which afford anti-Markovnikov hydroaminations have been reported. They are summarized in Figure 14 and the reactions in Table 6.

**Table 6:**
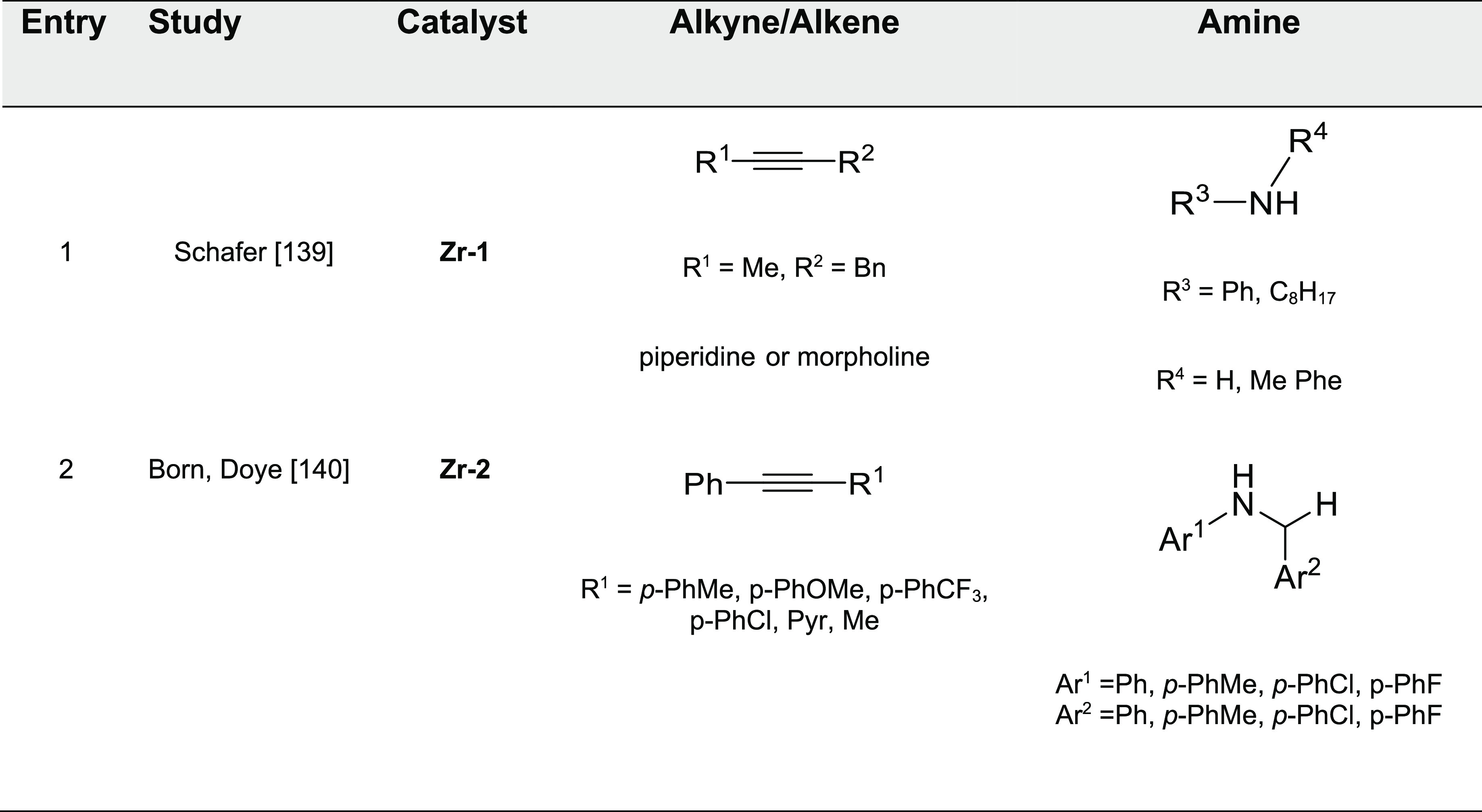
Experimentally Characterized Zirconium-Catalyzed Intermolecular Hydroaminations with Anti-Markovnikov Regioselectivity

**Scheme 32:**
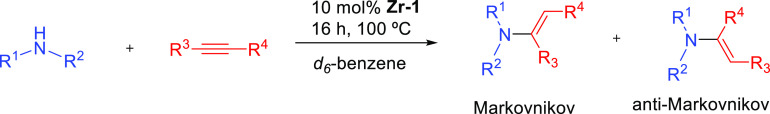
Hydroamination Reactions of Terminal Alkynes Catalyzed by Ureate-Supported Zirconium Catalysts^139^

In 2009, Schafer and co-workers showed that ureate-supported zirconium precatalyst (**Zr-1**) was highly effective in the intermolecular hydroamination of alkynes and the intramolecular hydroamination of alkenes (Scheme 32).^139^ This promising catalyst **Zr-1** was prepared on multigram scale from readily available, inexpensive materials in high yield following a protonolysis methodology. The catalytic activity (10 mol %, 100 °C, 16 h) was evaluated in the hydroamination of both terminal and internal phenyl-substituted alkynes. At elevated temperature, the reaction was regioselective, favoring the formation of the anti-Markovnikov regioisomer, except for internal alkynes, which showed low reactivity, presumably due to increased steric bulk.

**Scheme 33:**
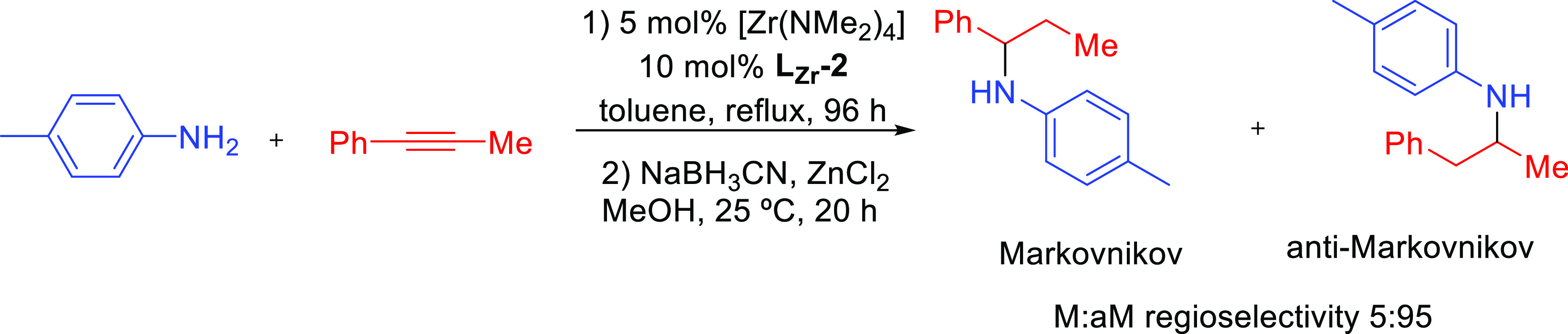
Hydroamination Reactions of Internal Alkynes Catalyzed by Sulfonamide-Based Zirconium Catalysts^140^

In 2012, Born and Doye prepared a zirconium catalyst formed in situ by combination of Zr(NMe_2_)_4_ and sulfonamide ligands **L**_**Zr**_**-2** which efficiently catalyzed the formation of the anti-Markovnikov product in the intermolecular hydroamination of 1-phenylpropyne with primary amines.^140^ The ligand structure had an important influence on the regioselectivity of the addition reaction. In this regard, sterically demanding tosylamide ligands such as *N*-(*tert*-butyl)-*p*-toluenesulfonamide (**L**_**Zr**_**-2b**) afforded the best results in terms of conversion and regioselectivity (Scheme 33). At elevated temperatures and long reaction times (160 °C, 96 h), hydroamination using 5 mol % [Zr(NMe_2_)_4_] and 10 mol % of a sulfonamide ligand **L**_**Zr**_**-2** could be performed with a wide scope of terminal and internal alkynes as well as with a broad variety of sterically demanding primary aromatic amines, leading to the obtention of the amine derivative after reduction of the imine hydroamination product with moderate to high yields. The use of aliphatic amines was reflected in lower yields and poor regioselectivities, ranging between 50:50 and 87:13. However, secondary amines were unreactive under equal conditions. Based on NMR studies using catalyst **Zr-2b**, the authors proposed that one ^t^Bu group and three NMe_2_ groups might be present in the generated catalytic species.

##### 3.5.2.2 Mechanistic Considerations and Factors Governing the Selectivity

**Scheme 34:**
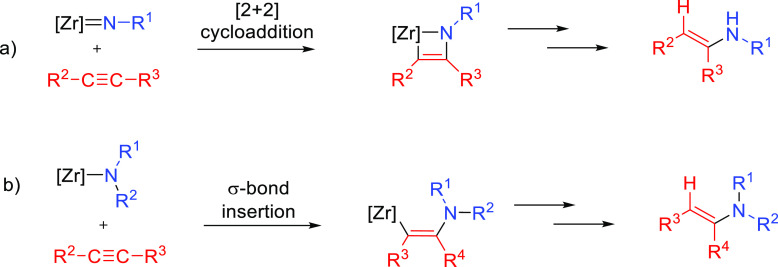
Two Proposed Pathways for Zirconium-Catalyzed Alkyne Hydroaminations

The mechanism of zirconium-catalyzed hydroaminations has been a matter of debate in the past decade.^32,113,141^ A σ-bond insertion of the unsaturated substrate into a metal–amido bond (Scheme 34b), akin to that established for group 3 and lanthanide metals, has been proposed as an alternative to the [2 + 2] cycloaddition mechanism commonly accepted for group 4 metal catalysts (Scheme 34a). Neutral zirconium-based catalysts have been reported to activate reactions with both primary and secondary amines in both intra-^113,139,142^ and intermolecular versions of this transformation.^139,141^ The imido mechanism can only use primary amines as substrates, and therefore, it is not possible for secondary amines. Thus, an insertion mechanism, with similarities to that reported for lanthanide-based catalysts (shown in Scheme 2) is proposed (Scheme 34b).^113^ Schafer and co-workers isolated and characterized vinylamine complexes resulting from electron-rich, nonpolar alkyne insertion into a Zr-NMe_2_ bond.^141^ The trapping of this insertion intermediate in this type of hydroamination reaction is a convincing evidence which clearly supports the insertion mechanism.^32^ A DFT study (BP86 functional) performed by Tobisch on the intramolecular hydroamination/cyclization of aminoallenes mediated by a [Cp_2_ZrCH_3_]^+^ zirconocene catalyst agrees with the intramolecular C=C insertion into the Zr—N σ-bond and the formation of the six-membered azacycle-Zr intermediate.^143^

### 3.6 Late Transition Metals

**Figure 15:**
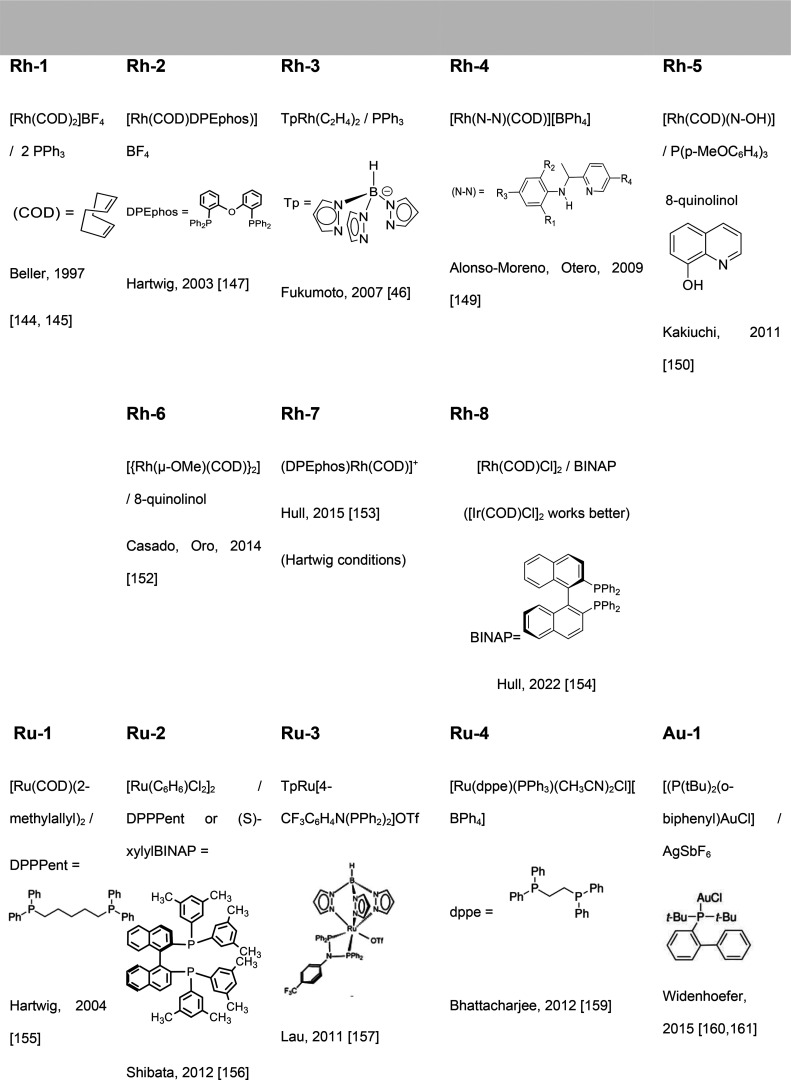

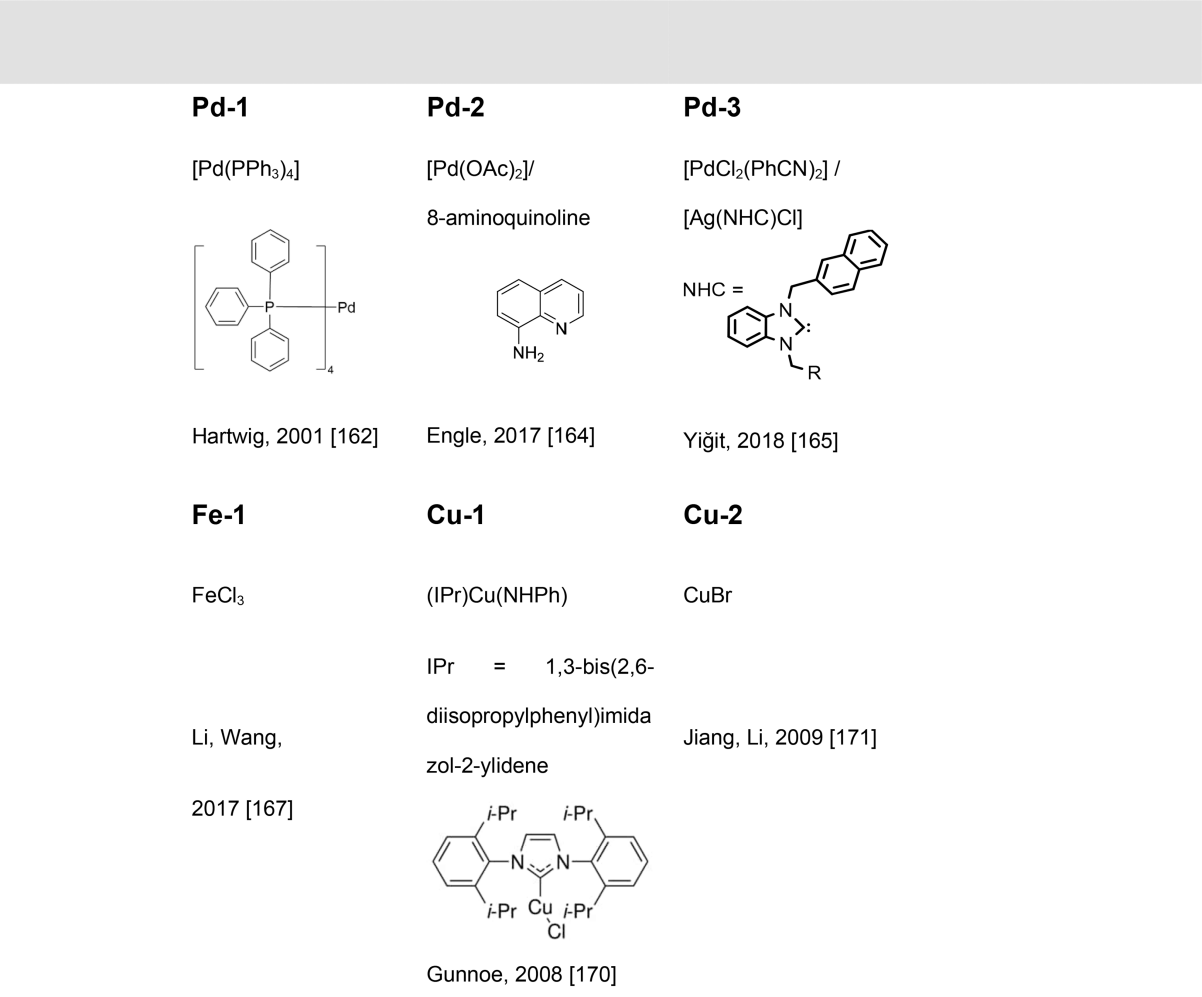
Late transition metal catalysts developed for anti-Markovnikov hydroamination.

In general, catalysts based on late transition metals are relatively stable to air and tolerant to a wide diversity of polar functional groups, whereas protected amines or directing groups are often required; moreover, limitations on the substrate scope are also found. Hydroamination processes catalyzed by late transition metals were exhaustively covered in several reviews.^3,4,33^ Herein, we will focus on those cases with anti-Markovnikov regioselectivity. The catalysts developed are collected in Figure 15, whereas the catalyzed processes are presented in Table 7.

**Table 7:**
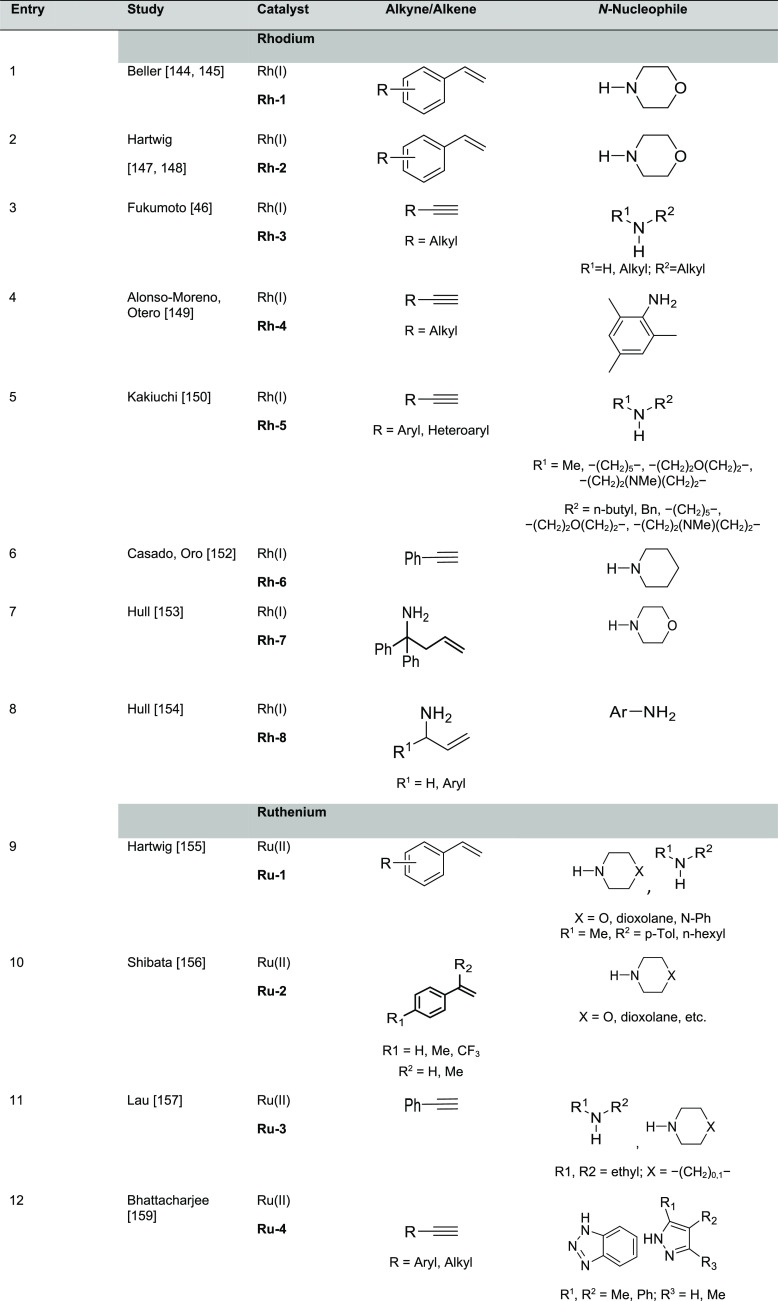

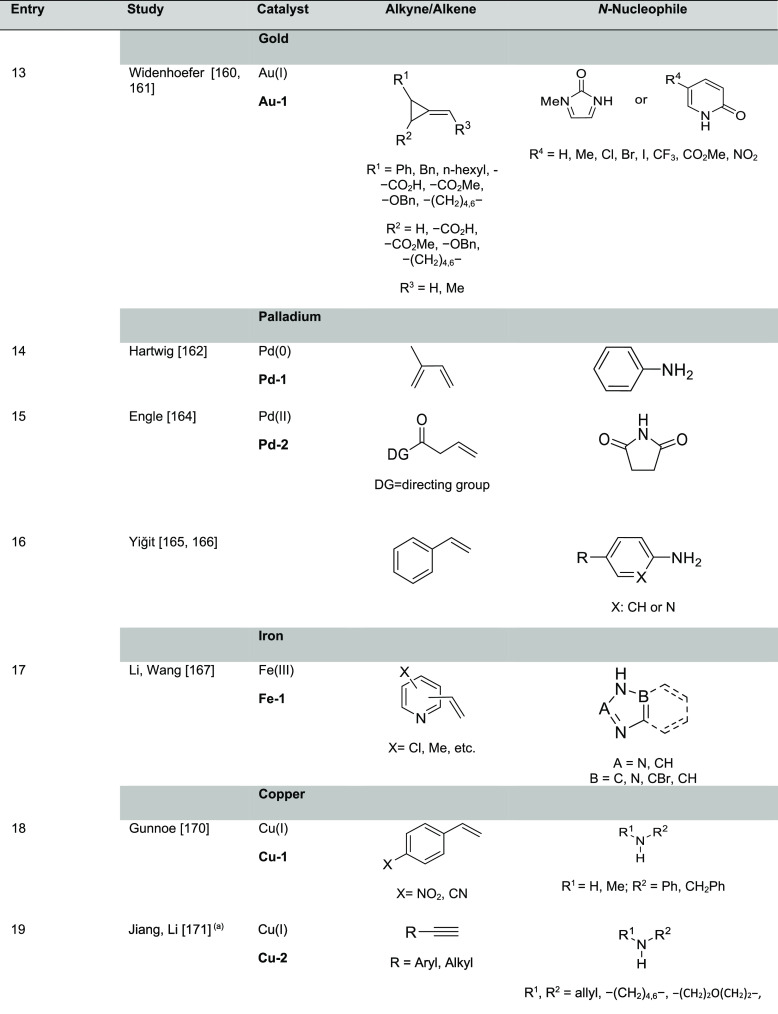
Nature of the C–C Multiple Bond Reactant and *N*-Nucleophile for the Late Transition Metal-Catalyzed Intermolecular Hydroaminations with Anti-Markovnikov Regioselectivity

(a) This reaction involves an anti-Markovnikov hydroamination and a subsequent alkyne addition to alkynes.

#### 3.6.1 Late Transition Metal-Based Catalysts

All the late transition metal-based catalysts developed for anti-Markovnikov hydroamination of unsaturated C–C bonds are collected in this section and grouped based on the metal center.

##### 3.6.1.1 Rhodium

**Scheme 35:**
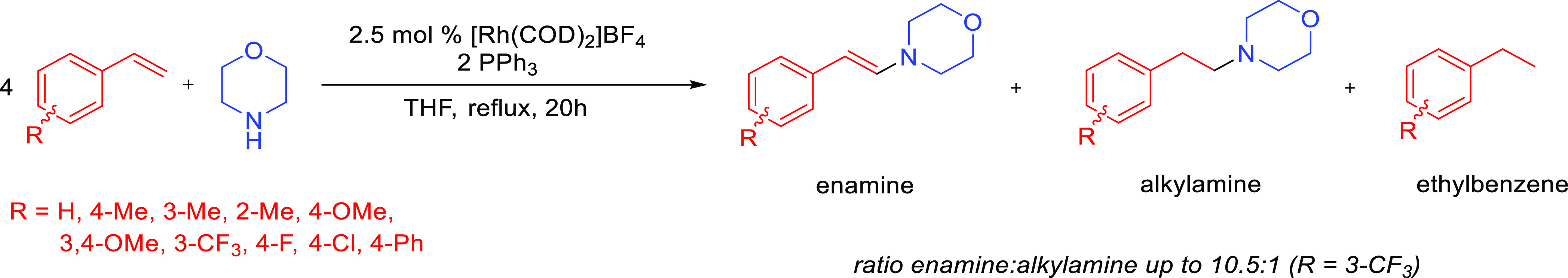
Rhodium-Catalyzed Amination of Styrenes and Morpholine^145^

In 1997 the group of Beller described the anti-Markovnikov oxidative amination of olefins.^144^ During the course of the catalyst screening, they found out that rhodium complexes with the general formula [RhL_4_]BF_4_ (L = olefin, phosphane) catalyze the selective formation of the enamines, giving rise to anti-Markovnikov products. In 1999, the same group reported the first intermolecular anti-Markovnikov hydroamination of styrene catalyzed by a cationic [Rh(COD)_2_]BF_4_/PPh_3_ complex, **Rh-1**, although with moderate yields due to the formation of the enamine (oxidative amination product; Table 7, entry 1). Under the optimized reaction conditions a styrene:amine ratio of 4:1 was used and the corresponding *N*-(2-phenylethenyl)morpholine and ethylbenzene were formed in good yields; the ratio enamine:alkylamine ranged from 2.1:1 (R = 3,4-OMe) to 10.5:1 (R = 3-CF_3_). It is worth mentioning that in most cases, the formation of ethylbenzene was significantly higher than that corresponding to the hydroamination product.^145^ In another study, the authors showed that the ratio of enamine to alkylamine is strongly influenced by the solvent, the phosphane, and the ratio of olefin to amine.^146^ Interestingly, when reaction was performed in THF at 120 °C with [Rh(COD)_2_]BF_4_ catalyst (2.5 mol %), an amine:enamine ratio of 49:1 was obtained, whereas in the presence of [Rh(COD)_2_]BF_4_/2 PPh_3_] as the catalytic system, the ratio was reversed (1:5) (Scheme 35).

**Scheme 36:**
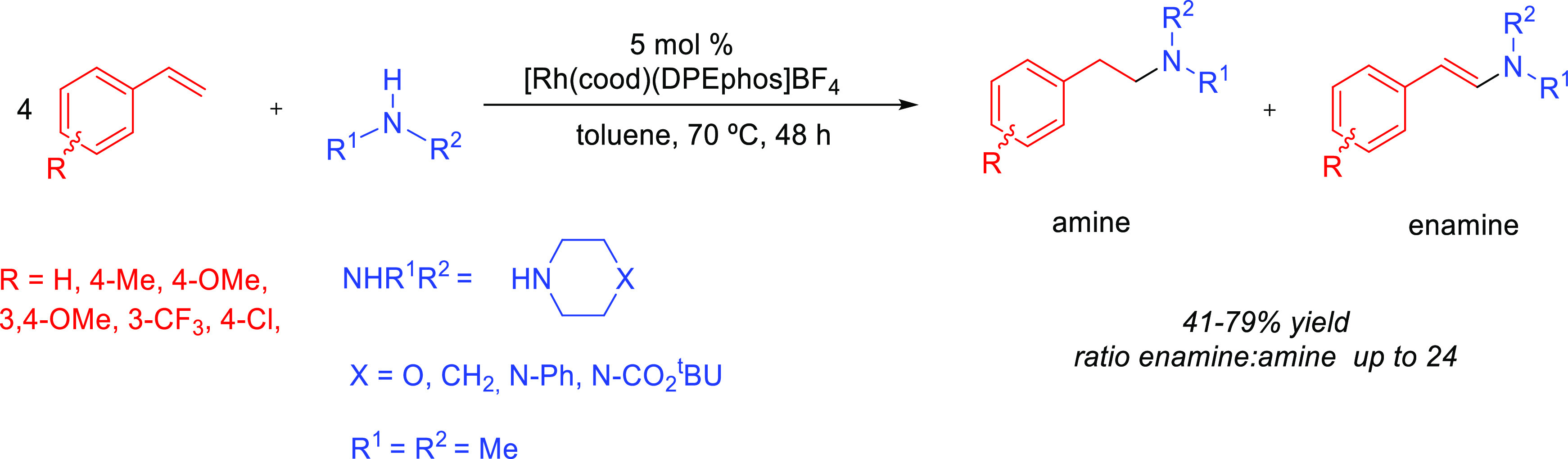
Rhodium-Catalyzed Anti-Markovnikov Hydroamination of Vinylarenes with Secondary Amines^148^

In 2003, Hartwig reported the selective hydroamination of alkenes to afford terminal amines as the major product catalyzed by [Rh(COD)(DPEphos)](BF_4_), **Rh-2**, (DPEphos = bis[(2-diphenylphosphino)phenyl] ether; Table 7, entry 2).^147^ They showed that the intermolecular anti-Markovnikov hydroamination of unactivated vinylarenes was the major product with secondary amines under the optimized reaction conditions (5 mol % cat, toluene, 70 °C, 2–3 days); the corresponding enamine (from the oxidative amination process) and ethylbenzene were also observed as minor products. The same group developed the intramolecular hydroamination process to generate *N*-containing heterocycles. The influence of the phosphine ligand was examined, obtaining the best results for the [Rh(COD)(DPPB)](BF_4_) (DPPB = 1,4-bis(diphenylphosphino)butane) complex (5 mol %, THF, 70 °C, 1–3 days), which favored the formation of anti-Markovnikov products from the intramolecular hydroamination of different *N*-methylaminopropyl)styrene derivatives (Scheme 36).^148^

**Scheme 37:**
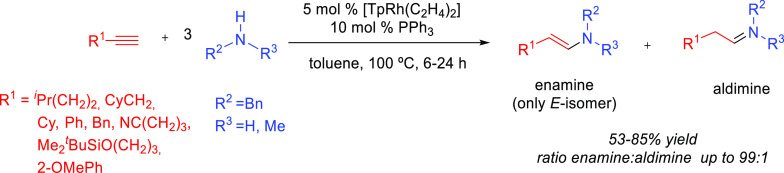
Rhodium-Catalyzed Anti-Markovnikov Addition of Primary and Secondary Amines to Terminal Alkynes^46^

In 2007 Fukumoto and co-workers published an anti-Markovnikov hydroamination for terminal alkynes with primary and secondary amines based on a rhodium catalyst, **Rh-3**, (Table 7, entry 3).^46^ They were able to describe the hydroamination of oct-1-yne with benzylamine or 1-octylamine catalyzed by a rhodium tris(pyrazolyl)borate system in moderate yields (10 mol %, toluene, 100 °C, 24 h). The use of both phosphine ligands and tris(pyrazolyl)borate was crucial to promote the formation of *E*-enamines and to avoid undesired alkyne dimerization. The reaction was shown to proceed more effectively for secondary amines than for primary amines, whereas the hydroamination of arylalkynes was unsuccessful (Scheme 37).

In 2009, the anti-Markovnikov addition of primary aromatic amines to terminal alkynes was also achieved by Alonso-Moreno, Otero, and co-workers with a Rh(I) complex (**Rh-4**) having iminopyridine-based bidentate nitrogen donor ligands ([Rh(N-N)(COD)][BPh_4_]; (N-N) = iminopyridine-based bidentate nitrogen donor ligands, i.e., 2,6-diisopropyl-*N*-[1-(pyridin-2-yl)ethylidene]aniline; Table 7, entry 4).^149^ This method catalyzes the regioselective formation of the *E* isomer of the corresponding imine in moderate to good yields (1.5 mol %, acetone-*d*_6_, 50 °C, 16–97 h).

**Scheme 38:**
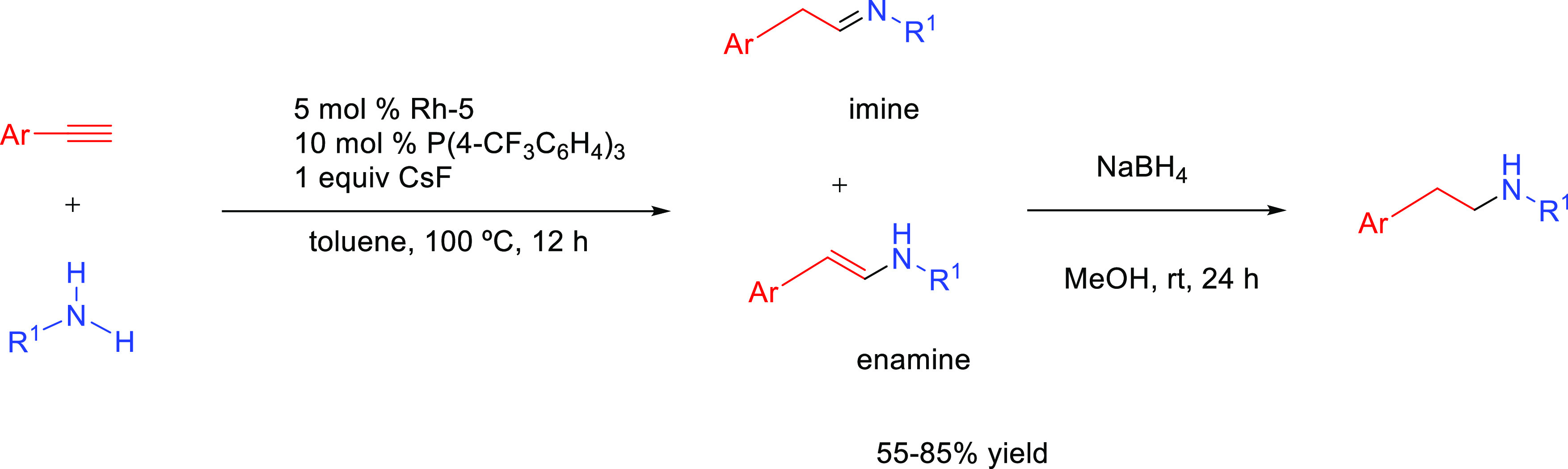
Rhodium-Catalyzed Anti-Markovnikov Hydroamination of Arylacetylenes with Primary Amines^151^

Kakiuchi and co-workers developed in 2011 an efficient protocol for the preparation of *E*-enamines by the anti-Markovnikov addition of secondary amines to terminal alkynes (Table 7, entry 5).^150^ The effective catalyst, **Rh-5**, was found to be a combination of an 8-quinolinolato Rh complex from [Rh(Cl)(COD)]_2_ precursor (10 mol %) and a P(*p*-OMeC_6_H_4_)_3_ phosphine (20 mol %). Remarkably, the reaction takes place at room temperature, and yields up to 87% were obtained. Recently, Kakiuchi’s group described the synthesis of enamines through an anti-Markovnikov hydroamination of terminal alkynes with primary amines.^151^ Under the optimized conditions, which involved the use of 5 mol % Rh catalyst precursor [Rh(8-quinolinonato)(COD)], 10% mol of P(*p*-CF_3_-C_6_H_4_)_3_ phosphine, CsF as a base, and toluene as solvent, alkylacetylenes and arylacetylenes reacted with primary amines at high temperatures (toluene, 100 °C) to afford aldimines, which were converted to the corresponding amines by reduction with NaBH_4_. In contrast, the reaction with secondary amines took place at room temperature due to their higher nucleophilicity. The reaction was successfully applied to a wide variety of aliphatic and aromatic substrates with reactive groups such as hydroxy, bromo, cyano, and thioesters, among others (Scheme 38).

Casado, Oro, and co-workers reported in 2014 a method based on a dinuclear precursor [{Rh(μ-OMe)(COD)}_2_], **Rh-6**, that was able to polymerize phenylacetylene in the presence of secondary amines (Table 7, entry 6). Interestingly, the reactivity is modified to obtain the anti-Markovnikov addition of the amine to the phenylacetylene by the addition of strong coordinating phosphines (P(*p*-OMeC_6_H_4_)_3_).^152^ In the described protocol (10 mol % Rh, 40 mol % phosphine, toluene, rt, 24–48 h), the catalytic species is generated by reaction of the Rh complex with 8-quinolinol, producing the complex [Rh(8-quinolinonato)(COD)] along with phosphines (P(*p*-OMeC_6_H_4_)_3_).

**Scheme 39:**
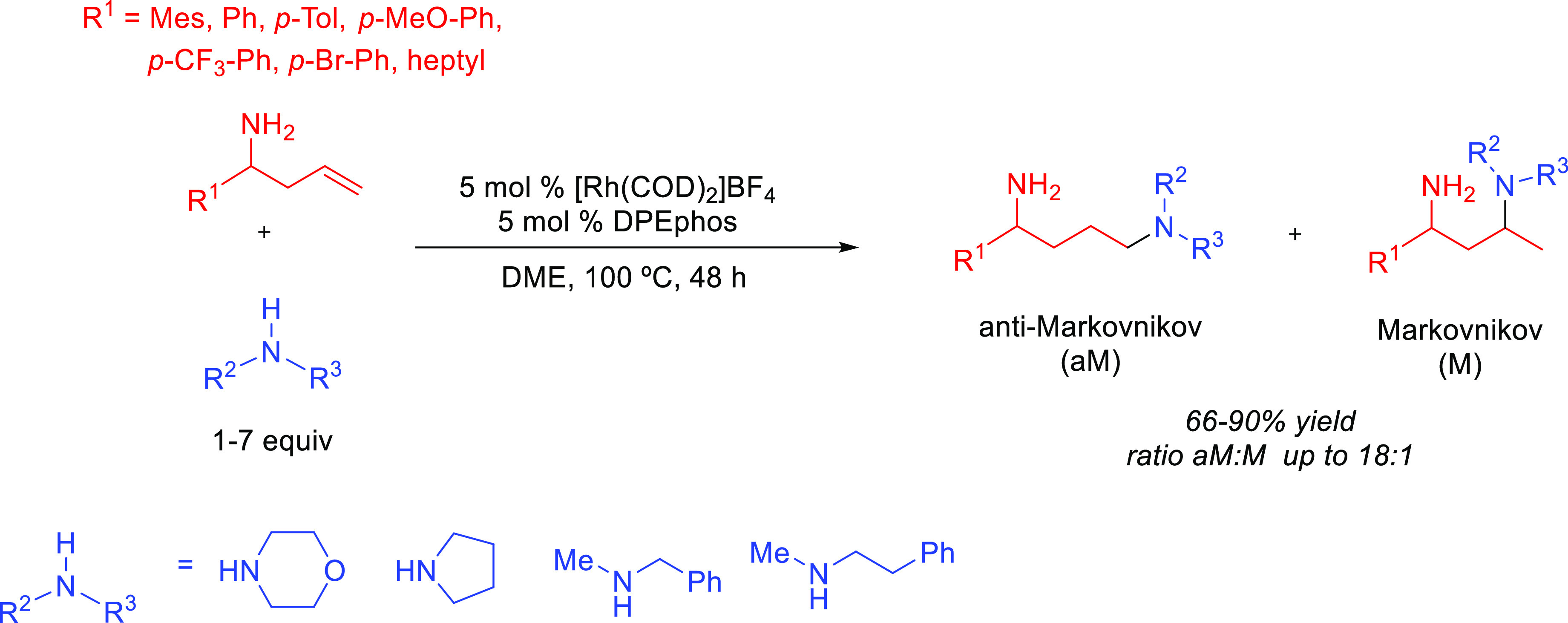
Rhodium-Catalyzed Anti-Markovnikov Hydroamination of Homoallylic Amines^153^

In 2015, Hull and co-workers established that the anti-Markovnikov hydroamination of homoallylic amines was feasible employing similar conditions to those developed by Hartwig (Table 6, entry 7), using the [Rh(COD)_2_]BF_4_ complex along with DPEphos ligand (5 mol % **Rh-7**, 5 mol % DPEphos, DME, 100 °C, 48 h).^153^ They hypothesized that coordination of a Lewis basic group (the amine from homoallylic amine) would promote an anti-Markovnikov selectivity. This was supported by comparing the reactions of 1,3-diamine and 1,4-diamine, giving rise to Markovnikov and anti-Markovnikov products, respectively. Interestingly, primary amines were identified as effective directing groups whereas no reaction was observed with carbamates or secondary amines. During their work they also observed that the distribution of products depends on the group present on the homoallylic amine and the ligand employed (Scheme 39).

In a very recent work, the Hull group developed a method for the synthesis of diamines by the hydroamination of allyl amines with aniline as nucleophile. The anti-Markovnikov regioselective product (with a 1:1.8 ratio) was obtained when using [Rh(COD)Cl]_2_ as catalyst and BINAP or DTBM-SEGPHOS and LiI as ligands and additives, respectively (1 mol % catalyst, 2.5 mol % DTBM-SEGPHOS, 1.5 equiv of LiI, toluene, 120 °C, 1–18 h), **Rh-8** (Table 7, entry 8).^154^ The regioselectivity was improved up to 1:6.4 ratio when the [Ir(COD)Cl]_2_ catalyst with the same BINAP ligand was employed.

##### 3.6.1.2 Ruthenium

**Scheme 40:**
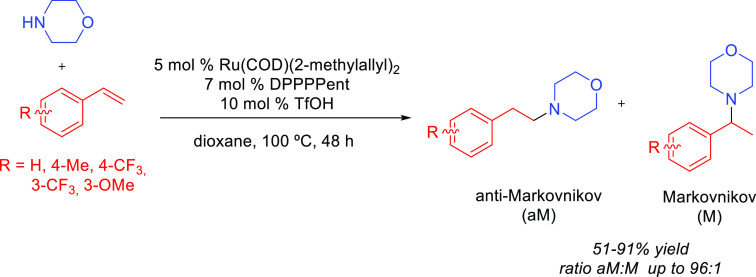
Ruthenium-Catalyzed Anti-Markovnikov Hydroamination of Vinylarenes^155^

In 2004, the Hartwig group developed a Ru-based catalyst ([Ru(COD)(2-methylallyl)_2_]/DPPPent (DPPPent = 1,5-bis-diphenylphosphinopentane)), **Ru-1**, that was able to efficiently catalyze the anti-Markovnikov hydroamination of vinylarenes with secondary amines and enhanced their previous results with Rh (Table 7, entry 9).^155^ By using this new catalyst, the reaction could be extended to α-methylstyrenes which underwent the hydroamination with modest conversions (Scheme 40). The anti-Markovnikov products could be obtained in yields up to 96% (5 mol % Ru catalyst, 7 mol % DPPPent, 10 mol % CF_3_SO_3_H, dioxane, 100 °C, 24 h) and excellent selectivities (>99%). It is worth mentioning that the presence of the phosphine ligand as well as triflic acid are indispensable for the success of these Ru-catalyzed reactions.

**Scheme 41:**
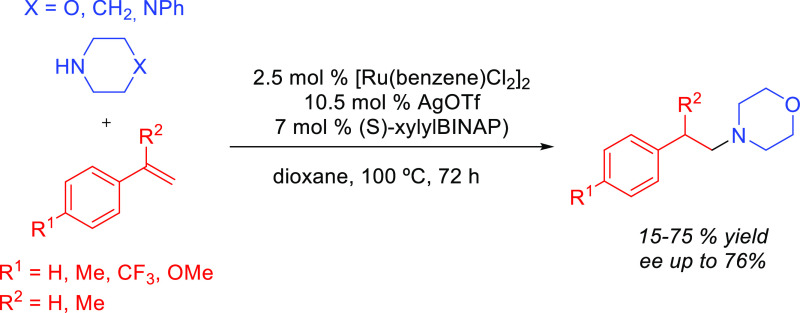
Ru-Catalyzed Chiral Anti-Markovnikov Addition of Amines to Styrenes^156^

Shibata and co-workers developed an enantioselective reaction using a related Ru catalyst but with a Ru precursor bearing a benzene ligand (Table 7, entry 10).^156^ They hypothesized that substitution of the benzene by a styrene derived reactant should facilitate the catalysis. The reaction works with [Ru(benzene)Cl_2_]_2_ (**Ru-2**), AgOTf, and a phosphine ligand (2.5 mol % [Ru(benzene)Cl_2_]_2_, 10.5 mol % AgOTf, 7 mol % (*S*)-xylylBINAP), without the need of strong acids. Remarkably, using a chiral phosphine, they were able to develop an enantioselective addition of amines to styrenes, obtaining the best results for (*S*)-xylylBINAP (Scheme 41).

In 2011, Lau and co-workers developed a process for the hydroamination of aromatic 1-alkynes by secondary amines based on a TpRu(II) complex. Concretely, they employed TpRu[4-CF_3_C_6_H_4_N(PPh_2_)_2_](OTf) (Tp = hydrotris-(pyrazolyl)borate), **Ru-3** (Table 7, entry 11).^157^ The catalytic system (0.5 mol % **Ru-3**, neat, 120 °C, 6 h) was originally developed for the addition of β-diketones to terminal alkynes, but the authors discovered that the catalyst was also effective for the hydroamination of terminal alkynes with moderate yields. During the screening process, they observed that whereas secondary amines were active, primary amines failed; besides, bulky and less nucleophilic amines were not reactive either. The anti-Markovnikov hydroamination of terminal alkynes was found to be highly selective, affording exclusively the *trans* products.

**Scheme 42:**
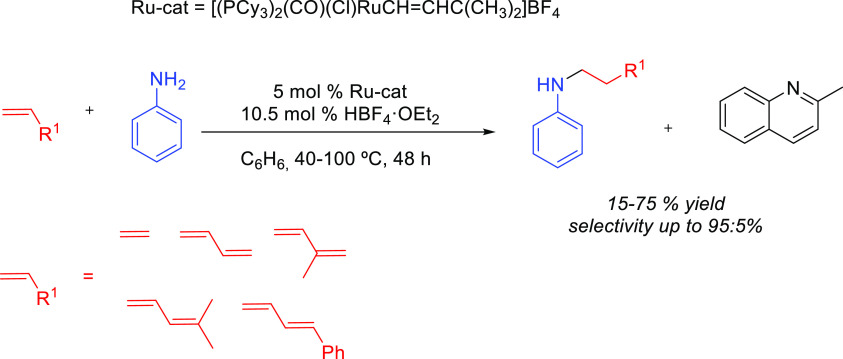
Ru-Catalyzed Anti-Markovnikov Addition of Arylamines to Ethylene and 1,3-Dienes^158^

Yi and Yun developed a Ru-based catalyst for the hydroamination of ethylene with aniline to deliver *N*-ethylaniline and 2-methylquinoline.^158^ The same work also reported an analogous reaction with 1,3-dienes; however, the formation of the Markovnikov addition products was favored, although the anti-Markovnikov addition was also observed for several substrates (Scheme 42). Therefore, the described methodology cannot be considered as an operative anti-Markovnikov hydroamination method.

In 2012, Bhattacharjee and co-workers developed a synthetic approach for the anti-Markovnikov addition of azoles to terminal aromatic and aliphatic alkynes, using **Ru-4** catalyst (Table 7, entry 12).^159^ Depending on the reaction conditions, the *E*-isomer of the enamine is obtained exclusively at temperatures higher than 100 °C in high yields (0.5 mol % **Ru-4**, toluene, 100–110 °C, 8–10 h).

##### 3.6.1.3 Gold

**Scheme 43:**
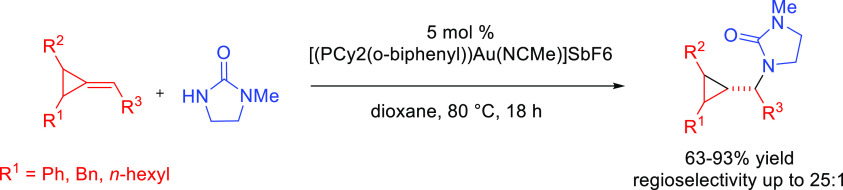
Gold-Catalyzed Anti-Markovnikov Hydroamination of Alkylidenecyclopropanes with Imidazolidin-2-ones^160^

Gold complexes have been also shown to be efficient catalysts for anti-Markovnikov hydroamination of unsaturated substrates. In 2015, Widenhoefer and co-workers reported the anti-Markovnikov intermolecular hydroamination of monosubstituted and *cis*- and *trans*-disubstituted alkylidenecyclopropanes by a cationic gold phosphine complex [{PCy_2_(*o*-biphenyl)}Au(NCMe)]SbF_6_ (Cy = cyclohexyl), **Au-1** (Table 7, entry 13).^160^ The reaction forms 1-cyclopropyl alkylamine derivatives with regio- and diastereoselectivity (5 mol % **Au-1**, dioxane, 80 °C, 16 h). Interestingly, this was the first example of a transition metal-catalyzed anti-Markovnikov hydroamination of an aliphatic and noncumulated alkene, as well as the first case of transition metal-catalyzed hydroamination of alkylidenecyclopropanes without ring opening. The reaction was then extended to the anti-Markovnikov hydroamination of methylenecyclopropanes (also monosubstituted and *cis*- and *trans*-disubstituted) using 2-pyridones as nucleophiles, as a new method for the *N*-alkylation of 2-pyridones (Scheme 43).^161^

##### 3.6.1.4 Palladium

**Scheme 44:**
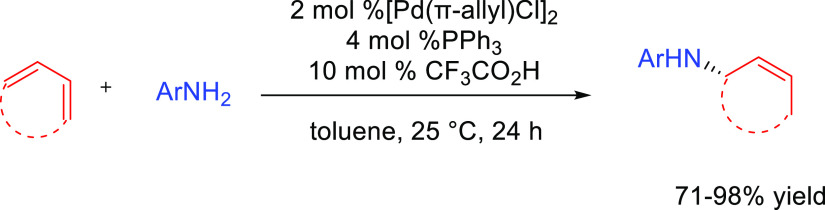
Pd-Catalyzed Anti-Markovnikov Addition of Arylamines to Dienes^163^

In the early 2000s, Hartwig’s group discovered by means of high-throughput screening studies that tetrakis(triphenylphosphine)palladium(0), [Pd(PPh_3_)_4_] (**Pd-1**) was a very efficient catalyst for the addition of aniline to dienes in the presence of trifluoroacetic acid (TFA) (Table 7, entry 14).^162,163^ They investigated several acids, observing that weaker acids than TFA were not suitable for the catalysis; however, triflic acid showed faster kinetics than other stronger acids. The reaction was developed for dienes such as cyclobutadiene or substituted 1,3-dienes such as 2-methyl-butadiene or 2,3-dimethyl-butadiene (2 mol % Pd(TFA)_2_, 4 mol % DPPF, 20 mol % TFA, 100 °C, 12 h). For the open dienes, the reaction gives the anti-Markovnikov product, in some cases almost exclusively (Scheme 44). The reaction also works for arylamines. The authors developed an asymmetric version of the process employing [Pd(π-allyl)Cl]_2_ precursor with chiral diphosphine ligands. Under the optimized conditions (2.5 mol % [Pd(π-allyl)Cl]_2_, 5 mol % ligand, neat, rt, 72–120 h), allylic amines were formed in high yields and enantioselectivities.

**Scheme 45:**
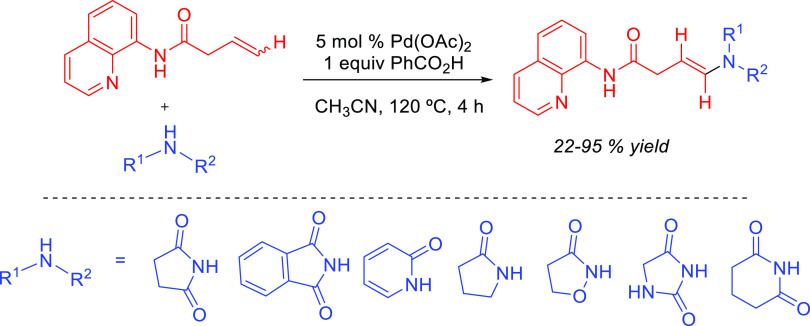
Pd-Catalyzed Anti-Markovnikov Hydroamination of Alkenes Using a Directed Nucleopalladation/Protodepalladation Strategy^164^

In 2017, Gurak and Engle proposed a strategy based on aminopalladation/protodemetalation to produce anti-Markovnikov hydroamination of alkenes with **Pd-2** (Table 7, entry 15).^164^ A key part of the strategy was selecting the proper auxiliary ligand that favors these two reactions (i.e., 8-aminoquinoline). For that purpose, they also needed to incorporate a directing group on the alkene to help with its coordination and activation, as well as to prevent the β-hydride elimination. They succeeded for several directing groups employing Pd(II)-based catalysts (alkene (1.0 equiv), succinimide (1.0 equiv), Pd(OAc)_2_ (10 mol %), acid (1.0 equiv) (Scheme 45).

In 2018, Yiğit and co-workers developed Pd(II)-N-heterocyclic complexes as catalysts (**Pd-3**) for the anti-Markovnikov hydroamination of styrene in ionic liquids (*N*-butylpyridinium hexafluorophosphate) (Table 7, entry 16).^165^ Under the optimized conditions (1 mol % [PdCl_2_(PhCN)_2_], CH_2_Cl_2_, rt, 24 h), the reactions were highly selective, observing only the formation of the anti-Markovnikov addition product in high yields. The methodology was then extended to other Pd(II)-pyridine-based catalysts with benzimidazolium and *N*-heterocyclic carbene ligands.^166^

##### 3.6.1.5 Iron

**Scheme 46:**
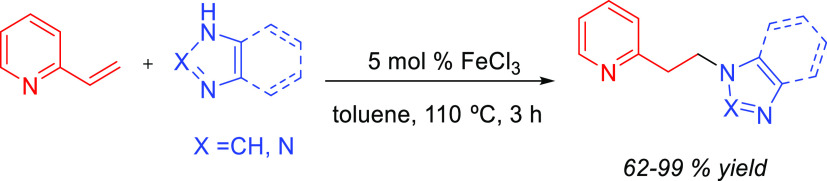
Iron-Catalyzed Anti-Markovnikov Hydroamination of Vinylpyridines with Azoles^167^

In 2017, Li, Wang, and co-workers published a Fe(III)-catalyzed hydroamination of vinylpyridines with azoles (Table 7, entry 17).^167^ The reaction is catalyzed by FeCl_3_ (5 mol % **Fe-1**) in toluene at 110 °C, affording the anti-Markovnikov product in excellent regioselectivities and moderate to excellent yields in short reaction times (3 h). Once the reaction conditions were optimized (1.5 mmol 2-vinylpyridine; 1.0 mmol benzotriazole; 0.05 mmol FeCl_3_), they investigated the scope of both reactants, vinylpyridines and azoles. They observed that in most cases the hydroamination provides the anti-Markovnikov product in good to excellent yields (Scheme 46). Interestingly, they screened other first-row transition metals (FeCl_2_, Cu(OTf)_2_, Zn(OTf)_2_, Sc(OTf)_3_, CuCl_2_, and ZnBr_2_), revealing that all these compounds were also successful with high to excellent yields for the anti-Markovnikov hydroamination reaction.

##### 3.6.1.6 Cobalt

In 2013, Astruc and co-workers reported that ethynylcobalticinium complex reacts with both primary and secondary amines, generating the anti-Markovnikov addition product corresponding to cobalticinium *trans*-enamines.^168^ Nevertheless, it seems that the cobalt complex is only a reactant, and it does not play any role as a catalyst in this process. Therefore, despite that it is here commented, it is included neither in Figure 15 nor in Table 7.

A recent publication showed that a divergent Markovnikov and anti-Markovnikov hydroamination under cobalt catalysis can be achieved by modification of the addition sequence of the reagents.^169^ In this communication, Ye and co-workers employed catalytic amounts of a Co(salen) metallic complex to afford the Markovnikov hydroamination of styrenes with moderate to excellent yields (42–93%) upon the addition of *N*-fluorobenzenesulfonimide (NFSI), which acted as oxidant and aminating agent, and 9-borabicyclo[3.3.1]nonane (9-BBN) as the hydrogen source (10 mol % Co catalyst, 2.5 equiv of NFSI, 1.5 equiv of 9-BBN, EtOAc, 60 °C, 10 h). However, if NFSI was added to a mixture of the cobalt complex and 9-BBN, then, the anti-Markovnikov product was isolated with moderate to good yields (33–62%).

##### 3.6.1.7 Copper

In 2008, Gunnoe and co-workers published the anti-Markovnikov hydroamination and hydrothiolation of activated (electron deficient) styrenes by Cu(I) amido and thiolate complexes (IPr)Cu(X) {X = NHR or SR; IPr = 1,3-bis(2,6-diisopropylphenyl)imidazol-2-ylidene} (**Cu-1**, Table 7, entry 18).^170^ The use of an excess of amine (aniline or benzylamine) is required for the reaction to produce significant or complete conversion (5 mol % (IPr)Cu(NHPh), C_6_D_6_, 80–120 °C, 21 h). This is the only anti-Markovnikov hydroamination described so far for copper. The other processes described with this metal are not direct hydroaminations.

**Scheme 47:**
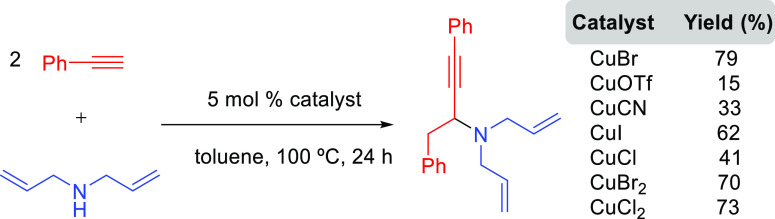
Copper-Catalyzed Reaction between Phenylacetylene and Diallylamine

Jiang, Li, and co-workers developed a tandem anti-Markovnikov hydroamination and alkyne addition to alkynes for the synthesis of propargylamines catalyzed by a Cu catalyst (5 mol % CuBr, **Cu-2**, Table 7, entry 19).^171^ According to their mechanistic proposal, the reaction proceeds by hydroamination of alkyne followed by the addition of a second alkyne instead of dimerization of alkyne and subsequent addition of amine to enyne. Thus, the first reaction consists of the anti-Markovnikov addition of the amine to the Cu(I)-activated alkyne (Scheme 47).

#### 3.6.2 Mechanistic Details for Direct Anti-Markovnikov Hydroaminations

In the previous section, the catalysts developed for the anti-Markovnikov hydroamination of alkenes and alkynes were gathered based on the metal center. In this section, they are discussed according to their proposed reaction mechanism for the catalytic processes. A general classification of the hydroamination reaction mechanisms was performed in section 2. The mechanisms proposed for direct hydroaminations using late transition metals can be divided in two main types: C–C multiple bond activation mechanisms and amido mechanism. There are no examples described by means of amino activation or imido mechanisms.

##### 3.6.2.1 C–C Multiple Bond Activation Mechanism

As outlined in section 2.1.3, this hydroamination mechanism can be divided in three different subtypes: (i) nucleophilic attack on an η^2^-coordinated C–C unsaturated substrate, (ii) nucleophilic attack on a vinylidene intermediate, and (iii) nucleophilic attack on an arene-coordinated substrate. The first is the most common mechanism, whereas the second pathway can only be found for alkynes. The third mechanism has only been described for two processes up to now.

*3.6.2.1.1. Nucleophilic Attack on an η^2^-Coordinated Substrate.* The following processes share in their mechanism that the C–C multiple bond is activated by the catalyst through a η^2^-coordination to the metal center.

**Scheme 48:**
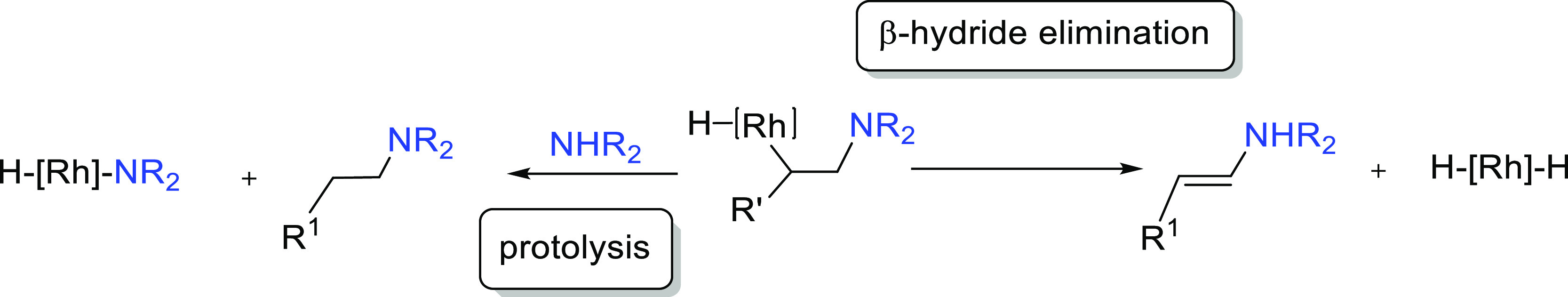
Alternative Reactions of the Postulated H-[Rh]-Alkyl Intermediate to Produce Hydroamination and Oxidative Amination^145^

*3.6.2.1.1.1. Rhodium.* The first Rh-catalyzed anti-Markovnikov hydroamination (and oxidative amination) of alkenes was developed by Beller’s group in 1997 (Table 7, entry 1).^144^ Regarding the mechanism, they investigated both oxidative amination and hydroamination processes. Initially, they discarded the formation of the hydroamination product by hydrogenation of the enamine (formed by oxidative amination of the olefin).^145^ According to their experimental evidence, the alkylamine is probably formed by the protolysis of the corresponding alkyl rhodium complex, instead of by an enamine hydrogenation. The analysis of *N*-deuterated amines with styrene, in the presence of a Rh catalyst, [Rh(COD)_2_]BF_4_/2PPh_3_, led to a scrambling of deuterium; most of the deuterium is incorporated in all olefinic positions of the styrene. Thus, the authors concluded that rhodium–hydride complexes are present in the reaction route. The H-[Rh]-alkyl intermediate may evolve to the formation of the hydroamination or the oxidative amination products (Scheme 48). The olefin and catalyst concentrations are important, as revealed by kinetic investigations: The kinetic analysis indicated that the rate-determining step involves the reaction of the rhodium species with the olefin. Overall, an olefin activation mechanism was suggested. All these observations for the reaction mechanism fit with the different routes shown in Scheme 48.

**Scheme 49:**
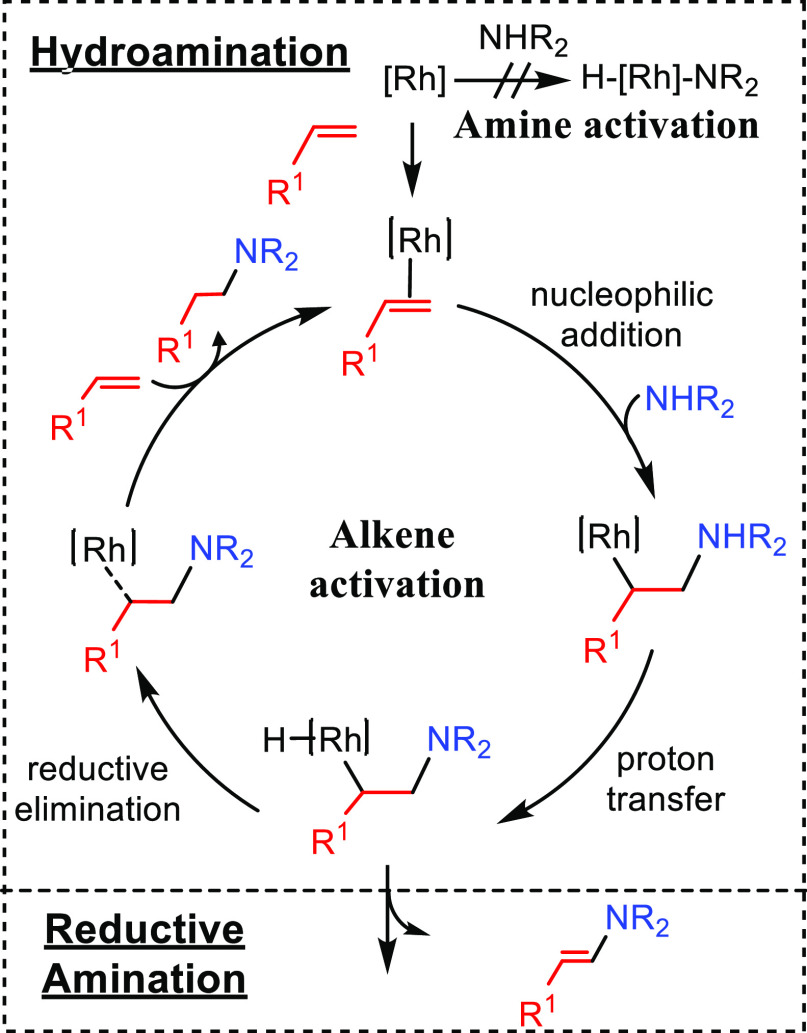
Computed Mechanisms for the Hydroamination of Alkenes by [Rh(DPEphos)]^+^ ^172^

For Hartwig’s Rh(I)-based system, that is somehow similar to that of Beller, a discrimination between amine or alkene activation mechanisms could not be clearly obtained by experiment (Table 7, entry 2).^148^ A computational study on the [Rh(DPEphos)]^+^ catalyst (DFT calculations with the M06 functional) showed that the alkene activation pathway is with no doubt the one at work.^172^ The amine activation pathway is much higher in energy, with an energy barrier higher than 50 kcal mol^–1^. The experimental mechanistic analysis also revealed that enamine (from oxidative amination) and amine (from hydroamination) are formed in parallel, not in sequential steps. In full agreement, the computational study disclosed that the formation of both products could be explained by the alkene activation mechanism (Scheme 49).^172^

**Figure 16:**
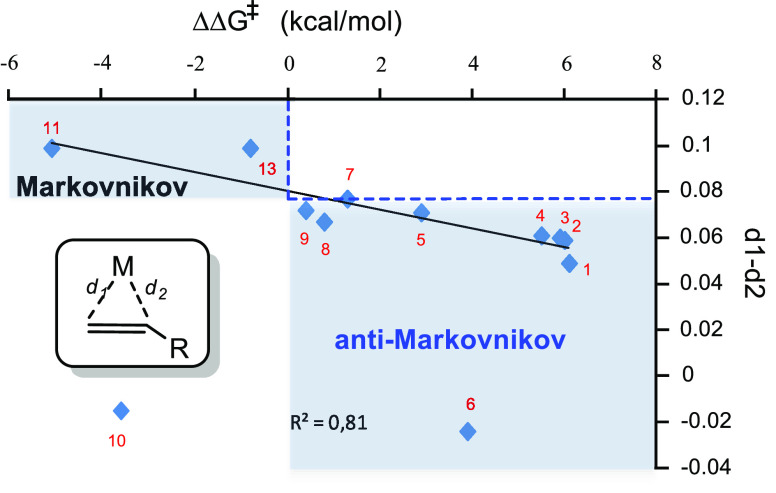
Plot of ΔΔ*G*^‡^ (the difference between the Markovnikov and anti-Markovnikov barriers (kcal/mol) against the *d*_1_ – *d*_2_ parameter (Å) for a set of terminal alkenes.^172^ M = Rh. Adapted with permission from Chem. Eur. J.2016, 22, 9311–932027226329. Copyright 2016 John Wiley and Sons.

The origin of the regioselectivity is defined as the nucleophilic addition step of the amine to the coordinated alkene. Analysis of the barriers for both Markovnikov and anti-Markovnikov pathways with the Activation Strain Model (ASM)^173,174^ supports the critical role of the distortion term: for both pathways the interaction energies along the reaction coordinate are similar; the main difference lies on the term associated with the distortion energy (the energy necessary to distort reactants from their original geometry to that in the transition state structure). A symmetrically η^2^-coordinated olefin is not activated toward a nucleophilic attack. Indeed, a η^2^ to η^1^ slippage of the olefin is required to facilitate the nucleophilic attack.^175,176^ Certainly, a good correlation was found between the difference of the Markovnikov and anti-Markovnikov barriers and the geometrical parameters measuring the degree of slippage (Figure 16). Besides, alkene coordination to [Rh(DPEphos)]^+^ leads to a LUMO with significant contribution of a p orbital from the terminal C atom, thus driving the regioselectivity for this process to the anti-Markovnikov product.^172^

**Scheme 50:**
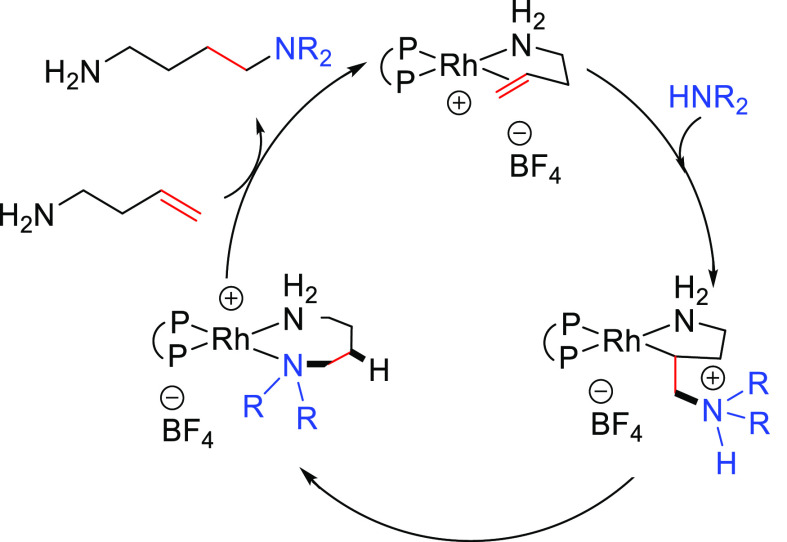
Proposed Mechanism for the Rh-Catalyzed Anti-Markovnikov Hydroamination of Homoallylic Amines (L = Amine)^153^

Hull and co-workers developed the anti-Markovnikov hydroamination of homoallylic amines (Table 7, entry 7). Their mechanistic proposal is presented in Scheme 50.^153^ The homoallylic amine is supposed to coordinate bidentately through the alkene and amine functional groups. Next, the nucleophilic attack by an external secondary amine onto the alkene affords a metallacyclic intermediate. This is the regioselectivity-determining step; under optimized conditions, the nucleophilic addition on the terminal carbon atom takes place. According to the authors, the bidentate nature of the substrate should help the formation of the proper metallacycle to obtain the anti-Markovnikov product. Then, the authors suggest proteolytic cleavage of the Rh–C bond or proton transfer/reductive elimination to generate the final product. Finally, ligand exchange of the hydroaminated product with the homoallylic amine closes the catalytic cycle.

*3.6.2.1.1.2. Gold.* It is well-known that gold(I) strongly coordinates C–C multiple bonds.^177−181^ The mechanism for the anti-Markovnikov hydroamination of alkylidenecyclopropanes was proposed to involve an outer-sphere addition of the amine nucleophile over the coordinated alkene, followed by a protodeauration step (Table 7, entry 13).^160,161^ The reaction mechanism for this process was computationally evaluated by two groups, and both confirmed a mechanism involving nucleophilic addition of the amine and protodeauration steps.^182,183^ For the latter step the computational study revealed that the oxygen of the *N*-nucleophile (1-methyl-imidazolidin-2-one), as well as a second molecule of *N*-nucleophile are essential in the proton-transfer process.^183^ The regioselectivity, however, is defined in the first step, the nucleophilic addition, that was also identified as the rate-determining step.

**Figure 17:**
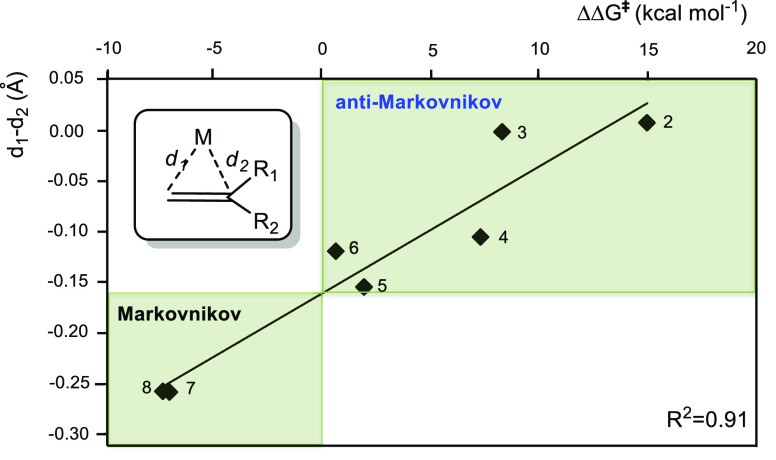
Correlation between the degree of slippage (*d*_1_ – *d*_2_ parameter) and the Gibbs energy difference (ΔΔ*G*^‡^) between Markovnikov and anti-Markovnikov addition barriers.^183^ M = Au. Adapted with permission from ACS Catal.2019, 9, 848–858. Copyright 2019 American Chemical Society.

The pathways for both regioisomers were computationally evaluated (DFT calculations with the M06 functional), concluding that the anti-Markovnikov addition is kinetically preferred, in agreement with experiment. The application of the Activation Strain Model revealed that the difference can be related to the comparatively inferior deformation energy required by the initial π complex to adopt the anti-Markovnikov transition state structure, compared to the Markovnikov one.^183^ Moreover, these authors found a good correlation between the Gibbs energy barrier and the back-bonding ability of the alkene for the anti-Markovnikov pathway; this reflects that the higher the capacity of the alkene for accepting electronic density from the transition metal fragment, the lower the energy barrier for the anti-Markovnikov addition. Interestingly, as for the Rh catalyst,^172^ the difference between the Markovnikov and the anti-Markovnikov barriers can be related to a structural parameter that measures the extent of slippage of the π bond of the coordinated alkene intermediate (from η^2^ to η^1^ coordination;^175^ see Figure 17). This observation is somehow related to that previously pointed out in the case of Rh (Figure 16). Finally, the coordination mode of the initial π complex is directly related to the regioselectivity observed in the process.

**Scheme 51:**
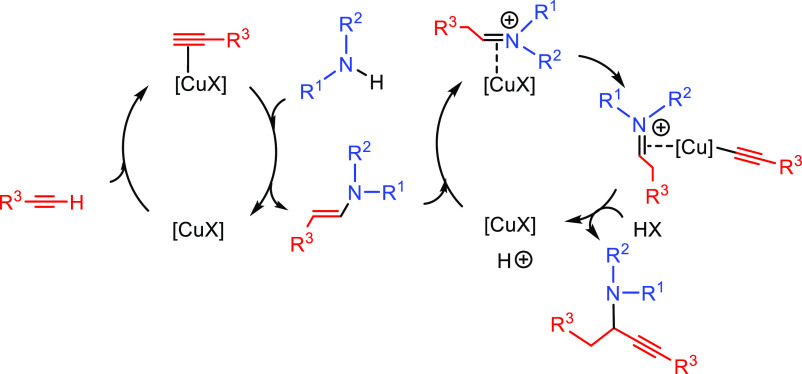
Proposed Mechanism for the Copper-Catalyzed Consecutive Hydroamination and Alkyne Addition to Alkynes^171^

*3.6.2.1.1.3. Copper.* The reaction mechanism for the tandem anti-Markovnikov hydroamination and alkyne addition to alkynes developed by Jiang, Li and co-workers is proposed to take place in two different steps (Table 7, entry 18, Scheme 51).^171^ The first step is a hydroamination to give an enamine, while the second is the addition of the alkyne to the formed enamine. The reaction mechanism for the hydroamination process can be classified within the C–C multiple bond activation. Thus, the first step involves the activation of the terminal alkyne by the Cu(I) complex. The secondary amine reacts with the coordinated alkyne to give an intermediate with the enamine formed. This intermediate is protonated to give an iminium intermediate. Subsequently, an intramolecular transfer of the new coordinated alkyne moiety to the iminium ion delivers the propargylamine final product and regenerates the copper catalyst. The hydroamination process may alternatively proceed via an acetylide intermediate. The alternative process where the alkyne addition is taking place first was discarded because such a product was not observed when the reaction was carried out in the absence of the amine.

**Scheme 52:**
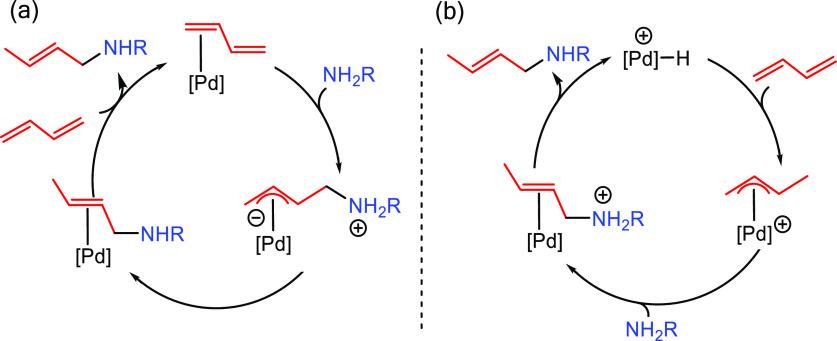
Proposals for Pd-Catalyzed Hydroamination of 1,3-Dienes: (a) in the Absence of Acid Cocatalyst; (b) in the Presence of Cocatalyst^162^

*3.6.2.1.1.4. Palladium.* Hartwig and co-workers developed a Pd-based catalyst for the hydroamination of dienes (Table 7, entry 14). According to their initial mechanistic studies, there are two possible pathways for the process.^162^ The first path involves attack by the amine to the coordinated η^2^-diene complex to form an allylpalladium product; this intermediate can undergo a proton transfer to generate free allylic amine. The second mechanism involves insertion of diene into a palladium hydride and external nucleophilic attack of the amine on the resulting η^3^-benzyl intermediate (Scheme 52). The authors suggested in their work that probably the first mechanism occurs in the absence of acid cocatalyst, while the second should take place when an acid cocatalyst is added. The first pathway can be associated with the C–C activation mechanism, whereas the second would be better described by a hydrometalation/reductive amination process.

**Scheme 53:**
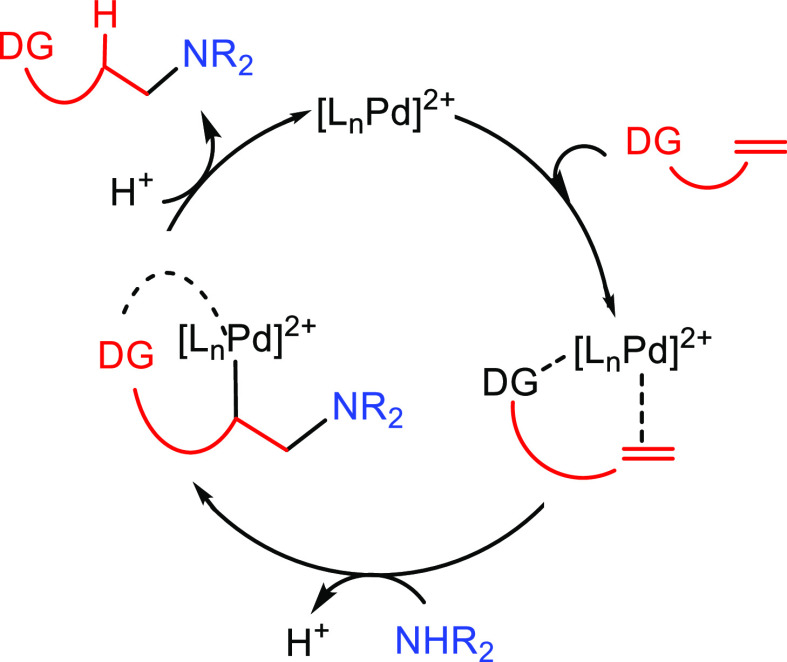
General Scheme for Pd(II)-Catalyzed Anti-Markovnikov Hydroamination of Alkenes Decorated with Proper Substituents (DG = Directing Groups) and Catalyst with Appropriate Auxiliary Ligands (L)^164^

Gurak and Engle developed what they described as an aminopalladation/protodepalladation strategy based on a Pd(II) catalyst (Table 7, entry 15).^164^ This strategy consists of the sequential combination of two different processes, aminopalladation and protodepalladation, to produce the formal regioselective hydroamination (Scheme 53). In order to accomplish the first process, aminopalladation, the alkene must bear a directing group and the catalyst needs the appropriate auxiliary ligands; these auxiliary ligands should favor the proper coordination and activation of the alkene to achieve the regioselective nucleophilic addition. Moreover, these ligands should also favor the nucleophilic addition over the β-hydride elimination (that is a very common process for Pd(II) but undesired for this strategy). They realized that the nucleophile adds *anti* to the directing group, therefore indicating that the process goes via an outer-sphere *anti* aminopalladation; therefore, this process belongs to the C–C multiple bond activation mechanistic group.

*3.6.2.1.2. Nucleophilic Attack on Vinilydene Intermediate (M=C=CRR′).*

**Scheme 54:**
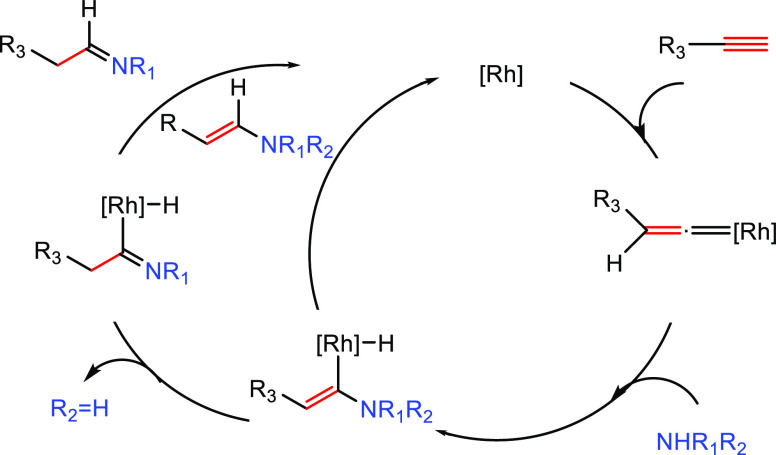
Fukumoto’s Proposed Mechanism for Rh(I)-Based Alkyne Anti-Markovnikov Hydroamination via Formation of a Rh-Vinylidene Intermediate^46^

*3.6.2.1.2.1. Rhodium.* Fukumotos’s proposal for the reaction mechanism for hydroamination of alkynes is different than that for other Rh-based anti-Markovnikov hydroamination catalysts (Table 7, entry 3).^46^ They propose that it also starts by an alkyne activation, but in this case, such an activation affords the formation of a Rh-vinylidene intermediate (Scheme 54). Then, the amine undertakes the nucleophilic addition on the α carbon atom, thus defining the regioselectivity for the process. Finally, the intermediate with the aminovinyl and hydride ligands undertakes a reductive elimination to generate the anti-Markovnikov hydroaminated product.

For the reaction developed by Kakiuchi and co-workers (Table 7, entry 5),^150^ they speculate that the reaction may proceed through nucleophilic attack of amines at the α carbon of vinylidene-rhodium intermediates, similarly to Fukumotos’s proposal. For the reaction developed by Alonso-Moreno, Otero, and co-workers (Table 7, entry 4), the authors could not discriminate between alkyne or amine activation pathways.^149^ For the reaction developed by Casado, Oro, and co-workers (Table 7, entry 6),^152^ they also suggested an initial activation of the alkyne, but they were not able to propose any mechanism with the available data.

**Scheme 55:**
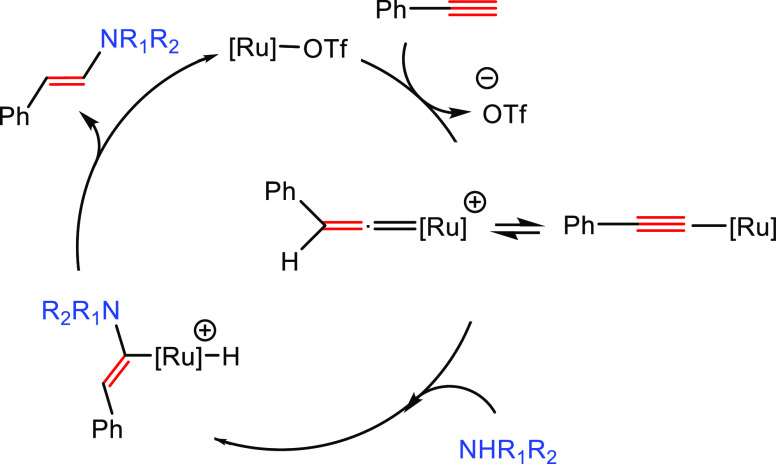
Proposed Mechanism for the Ru-Catalyzed Hydroamination of Alkynes Developed by Lau and Co-workers^157^ [Ru] = TpRu(4-CF_3_C_6_H_4_N(PPh_2_)_2_.

*3.6.2.1.2.2. Ruthenium.* The Ru-based catalytic system reported by Lau and co-workers was originally developed for the addition of β-diketones to terminal alkynes and then extended to the hydroamination (Table 7, entry 11).^157^ The reaction mechanism for the addition of β-diketones to terminal alkynes is described by the direct attack of the deprotonated β-diketone on the η^2^-coordinated alkyne. During their mechanistic investigation, they detected a Ru-vinylidene intermediate that evolves to the formation of a Ru-alkynyl species. They speculate with the protonation of this intermediate to form undetectable η^2^-coordinated alkyne that is subsequently attacked by the deprotonated β-diketone. For the case of hydroamination, however, they suggested that the mechanism is most probably going through the regioselective nucleophilic addition of the amine to the Ru-vinylidene intermediate to produce the anti-Markovnikov addition. The Ru-alkynyl intermediate was identified as the resting state, but assuming that it is not directly participating in the mechanism (Scheme 55).

**Scheme 56:**
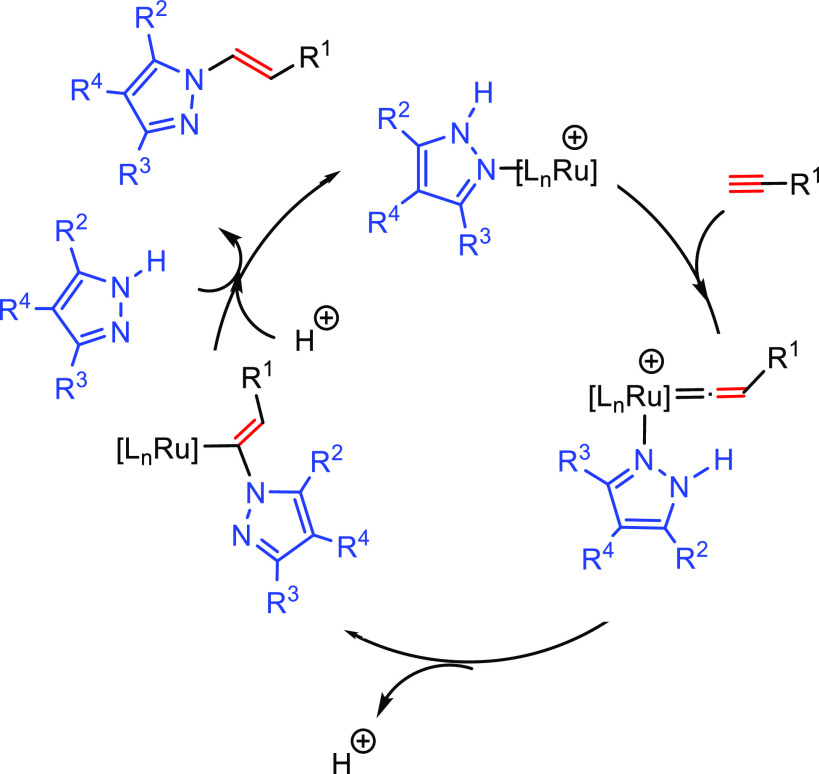
Proposed Reaction Mechanism for Ru-Catalyzed Anti-Markovnikov Hydroamination of Alkynes^159^^,^ [L_*n*_Ru]^+^ = [Ru(dppe)(PPh_3_)Cl]^*n*+^.

The reaction mechanism for the anti-Markovnikov hydroamination of alkynes was analyzed by Das and Bhattacharjee (Table 7, entry 12).^159^ According to their analysis, the first step is the replacement of a coordinated acetonitrile by the pyrazole on the coordination sphere of the Ru complex (Scheme 56). Then, there is the coordination of the alkyne and its rearrangement to a vinylidene species. Next, a nucleophilic attack by the pyrazole nitrogen to the coordinated vinylidene carbon takes place; the regioselectivity is decided in this step. Finally, the product is released by protonolysis and the active catalyst is regenerated.

*3.6.2.1.3. Nucleophilic Attack on Arene-Coordinated Substrate*

*3.6.2.1.3.1. Ruthenium*

**Scheme 57:**
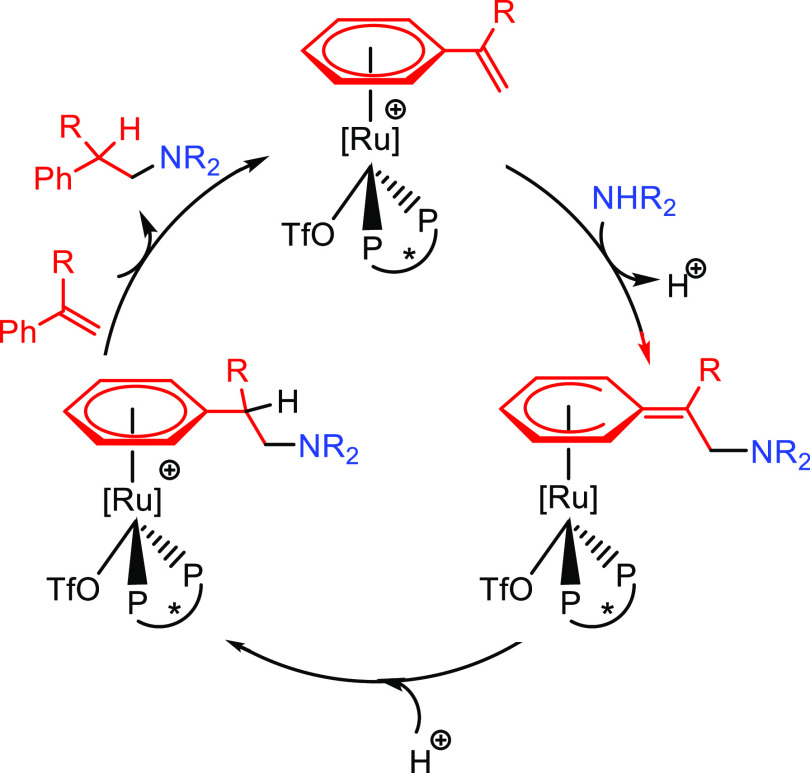
Proposed Reaction Mechanism for Ru-Catalyzed Anti-Markovnikov Hydroamination of Vinylarenes..^47^ Chiral phosphines introduce asymmetry in the process at the protonation step.

Mechanistic studies on the Ru-based hydroamination reaction developed by Hartwig show that the arene ligand of the catalyst is substituted by the vinylarene reactant to initiate the reaction (Table 7, entry 9; Scheme 57).^47^ The equilibrium between these two η^6^-vinylarene intermediates was supported by ^31^P NMR spectroscopy. Experimental observations also supported that the most likely mechanism involves the direct nucleophilic attack of the *N*-nucleophile to the terminus of the vinyl group (giving the anti-Markovnikov addition). Thus, the Ru catalyst activates the arene toward nucleophilic attack through η^6^–κ^1^ coordination. However, the reason why the addition is on the terminal carbon atom remains elusive. The intermediate formed after addition must lose a proton from the nucleophile, thus generating a formal negative ligand that should break the aromaticity of the ring. Protonating this ligand on the carbon atom generates the final product. When using chiral phosphine ligands, the chirality is introduced in this step since the proton enters from the opposite side of the Ru-metal center.^156^ Shibata developed a related Ru-based catalyst for the addition of amines to styrene with a similar mechanistic proposal (Table 7, entry 10).^156^

**Scheme 58:**
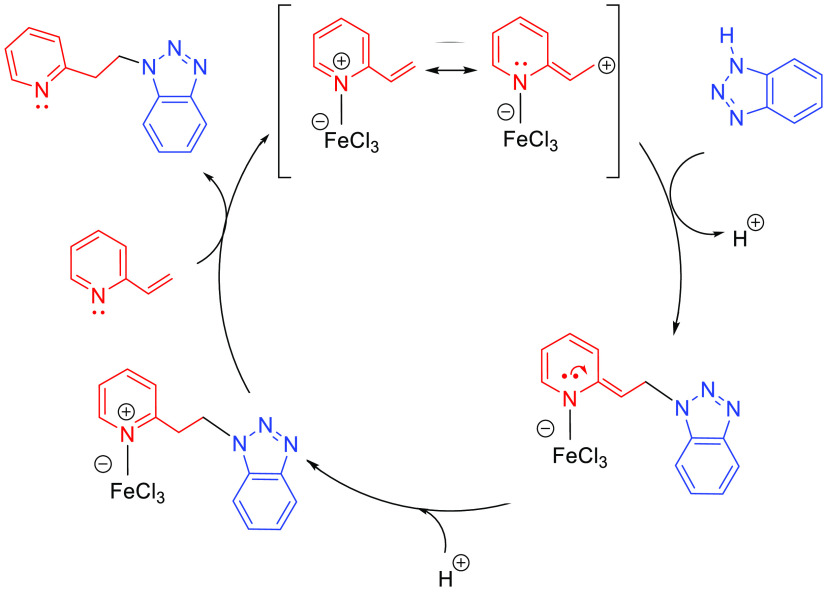
Proposed Mechanism for Fe-Catalyzed Hydroamination of Vinylpyridines with Azoles^167^

*3.6.2.1.3.2. Iron.* The proposed mechanism for the Fe-catalyzed hydroamination reaction of vinylpyridines with azoles (Table 7, entry 17) is shown in Scheme 58.^167^ The first step is the coordination of the 2-vinylpyridine to the FeCl_3_ by means of the N atom. The complex formed leads to a polarized zwitterionic intermediate with a formal positive charge on the N atom; by resonance, this charge can be located at the terminal carbon atom of the vinyl group. Then, the conjugate addition of azoles gives the anti-Markovnikov hydroamination intermediate. Such an intermediate needs to be protonated to form the zwitterionic complex with the coordinated product. Replacing the product by another vinylpyridine closes the catalytic cycle. In this mechanism, the presence of the metal catalyst produces the coordination of the vinylpyridine to the metal center through the N atom, thus conducting the reaction to undergo a 1,4-conjugated addition. In this way, the anti-Markovnikov regioselectivity is obtained. This process may be included in the C–C multiple bond activation mechanistic group.

##### 3.6.2.2 Amido Mechanism

 

**Scheme 59:**
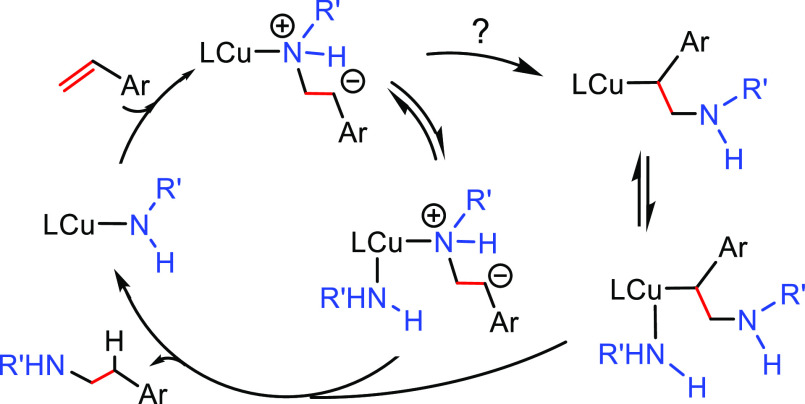
Proposed Mechanism for Cu(I)-Catalyzed Hydroamination of Electrondeficient Styrenes^170^

*3.6.2.2.1. Copper.* The anti-Markovnikov hydroamination of vinylarenes developed by Gunnoe^170^ suggested a mechanism where the amine is activated by the Cu(I) catalyst giving rise to a Cu-amido complex (Table 7, entry 18). The proposed mechanism assumes the formation of an initial Cu-amido intermediate (Scheme 59). Then, there is a nucleophilic addition of the amido ligand to the free alkene; regioselectivity is defined in this step. This was identified as the rate-determining step. An isomerization process of the amido ligand to produce an alkyl ligand may also be at work. Finally, a protodemetalation gives rise to the hydroaminated product. Such protodemetalation generates a new Cu-amido complex, therefore closing the catalytic cycle. The rate-determining step corresponds to the nucleophilic addition of the amido ligand to the free alkene. Overall, this process can be classified within the named amido mechanisms.

## 4 Formal Anti-Markovnikov Hydroamination

As commented in section 2.2, there have been developed several nondirect hydroamination processes to generate anti-Markovnikov hydroamination adducts. Conversely to most of the reactions described so far, these processes are not optimum from an atom economy point of view because they involve several reagents (different sources for H and N moieties) to accomplish the formal hydroamination of alkenes and alkynes. Among all the possibilities of combining reactions to obtain hydroaminated products, here we have selected those processes that have been specifically designed for obtaining the formal hydroamination of alkenes or alkynes. All of them involve transition metal catalysts. They are presented in section 4.1, whereas their mechanisms are described in section 4.2.

### 4.1 Description of the Processes

**Figure 18:**
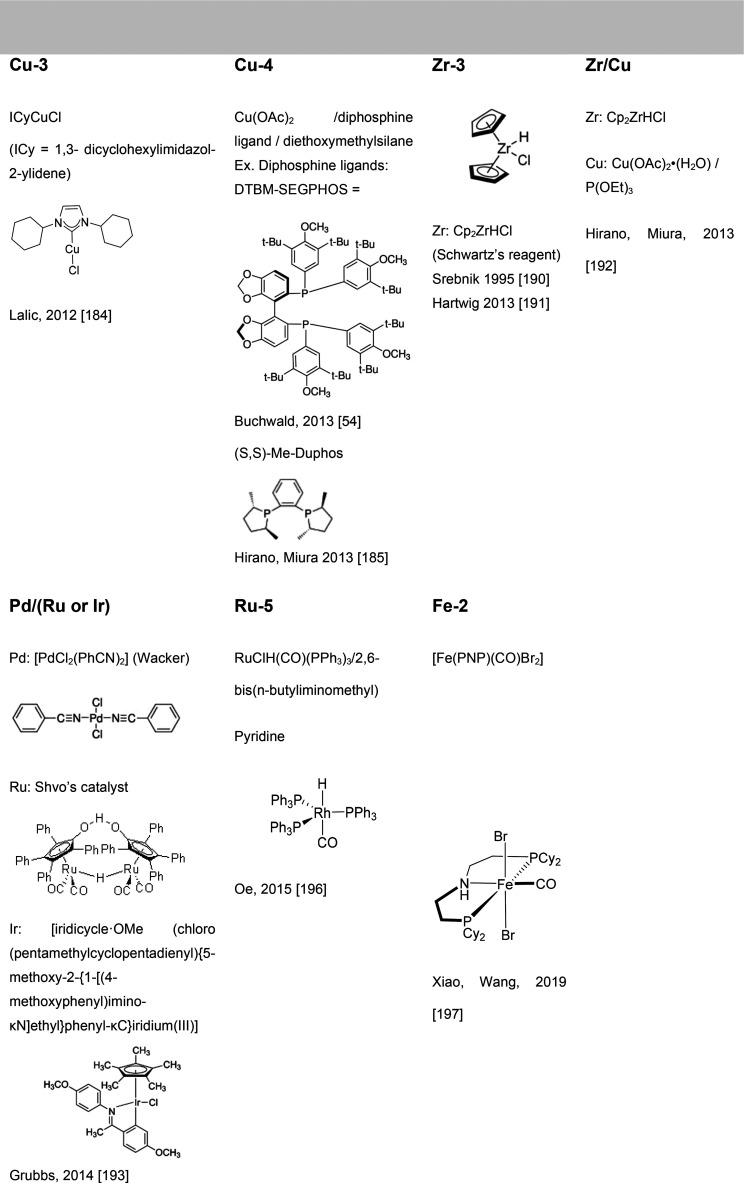
Transition metal catalysts developed for formal anti-Markovnikov hydroamination.

In Figure 18 and Table 8 are gathered the catalysts and type of processes developed to obtain formal hydroaminated products. They are grouped by the nature of the metal catalyst.

#### 4.1.1 Copper

**Scheme 60:**
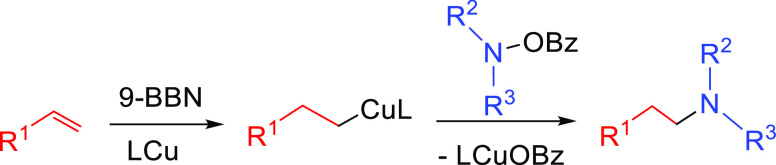
Formal Anti-Markovnikov Hydroamination of Terminal Alkenes Takes Place in Two Steps, Mediated by the Formation of Alkylboronate^,^^184^ Reaction based on a Cu catalyst. 9-BBN = 9-borabicyclo[3.3.1]nonane.

In 2012, Lalic and co-workers reported a two-step, formal anti-Markovnikov hydroamination of terminal alkenes, involving a Cu-based catalyst (ICyCuCl (ICy = 1,3-dicyclohexylimidazol-2-ylidene)) to generate tertiary amines (**Cu-3**, Table 8, entry 1).^184^ The general process is shown in Scheme 60 (5 mol % **Cu-3**, 1,4-dioxane, 45 °C, 6 h). The reaction proceeded in two steps, with the initial conversion of the alkene to an alkyl boronate ester and the subsequent replacement by the amine to produce the formal anti-Markovnikov hydroamination. In their work, the authors initially explored the hydroamination of 4-phenyl-1-butene using 9-BBN as a hydroboration reagent and *O*-benzoyl-*N*,*N*-dibenzylhydroxylamine as the electrophilic source of nitrogen; they extended the scope of the process to other alkenes and amines ((1.0 equiv), R_2_N-OBz (1.3 equiv, 0.2 M), *tert*-BuOM (1.3 equiv), ICyCuCl (0.05 equiv), 9-BBN (1.0 equiv), and *tert*-BuOLi (1.1 equiv)).

**Scheme 61:**
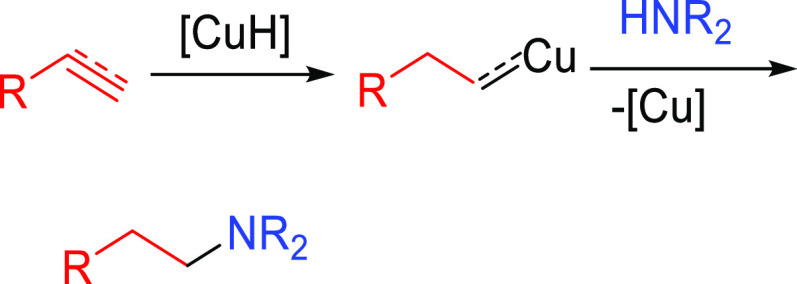
Formal Anti-Markovnikov Hydroamination of Alkenes and Alkynes Based on a CuH Catalysis

In 2013, the groups of Miura and Hirano^185^ and the group of Buchwald^186^ reported independently a novel approach for hydroamination of alkenes based on CuH catalysts (Scheme 61). They constructed their strategy based on a regiocontrolled hydrometalation of the alkene and a subsequent C–N bond formation through electrophilic alkene amination; they also required a suitable hydride source.^187^ This protocol is effective for an extensive variety of terminal alkenes and amine electrophiles giving access to a wide range of highly substituted chiral alkyl amines; the Markovnikov regioselectivity was observed in the protocols developed by both groups (Table 8, entries 2 and 8). In Buchwald’s work, however, the anti-Markovnikov addition to terminal aliphatic olefins was also accomplished. They employed the same optimized reaction conditions, Cu(OAc)_2_ (2 mol % Cu) and diphosphine ligands (e.g., (*R*)-DTBM-Segphos or (*S*,*S*)-Me_Duphos; Cu/L = 1:1.1), **Cu-4**, with diethoxymethylsilane (DEMS) serving as a suitable hydride source. Remarkably, when applied to terminal aliphatic alkenes, they observed linear tertiary amines in high yields.^188^ Thus, they developed an effective method for the regioselective anti-Markovnikov addition of amines to terminal aliphatic alkenes.

This methodology was then extended to alkynes.^189^ Depending on the reaction conditions, enamines or alkylamines could be selectively synthesized using the same starting materials. When protic additives were included (EtOH or *i*-PrOH), alkylacetylenes and arylalkynes were converted to linear alkylamines and α-chiral branched aliphatic amines, respectively. A series of enamines were efficiently obtained from the respective arylalkynes without using a proton source (HSiMe(OEt)_2_) but in the presence of an alcohol (2 mol % Cu(OAc)_2_, 2.2 mol % ligand, THF, 45 °C, 18 h). Excellent regio- and diastereoselectivities (>20:1 α:β amination, >20:1 *E*/*Z*) were observed for enamine products.

#### 4.1.2 Zirconium, Zirconium/Copper

**Scheme 62:**
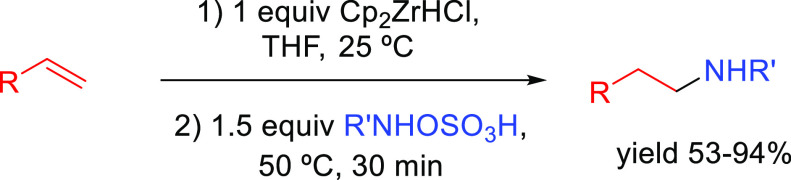
Anti-Markovnikov Amination of Alkenes via Hydrozirconation with *N*-Methylhydroxylamine-*O*-sulfonic acid^191^

In 1995, Zheng and Srebnik reported an anti-Markovnikov hydroamination of unactivated alkenes via a hydrozirconation catalyzed by Cp_2_ZrHCl (**Zr-3**, Schwartz’s reagent; Figure 18) followed by reaction with *O*-mesitylsulfonyl hydroxylamine to form primary alkylamines from olefins; the disadvantage of this process is that the hydroxylamine decomposes over time and requires a multistep synthesis.^190^ In 2013, based on the same strategy, Strom and Hartwig developed a one-pot anti-Markovnikov hydroamination of unactivated alkenes using the same zirconium complex (**Zr-3**) but employing *N*-methylhydroxylamine-*O*-sulfonic acid as electrophilic amine (an stable amine source) for the amination reaction (Scheme 62).^191^ Within this methodology (1 equiv of Cp_2_ZrHCl, 1.5 equiv of RNHOSO_3_H, THF, 50 °C, 30 min) primary and secondary amines could be synthesized from olefinic derivatives; this procedure works under mild conditions in good to high yields (53–94%) and without isolation of intermediates. Different commercially available hydroxylamine-*O*-sulfonic acids were assayed, yielding the desired product in high yields. The main advantages of this efficient methodology are the wide functional group tolerance and the short reaction times.

**Scheme 63:**
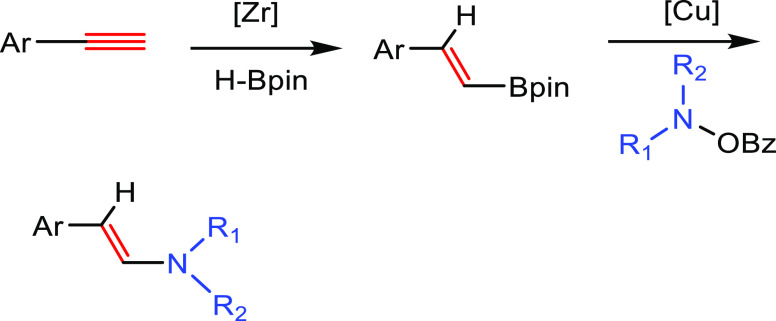
Formal Anti-Markovnikov Hydroamination of Alkynes Based on a Sequential Zr/Cu Catalysis.^192^ Bpin = 4,4,5,5-tetramethyl-1,3,2-dioxaborolanyl.

In 2013, Hirano, Miura, and co-workers developed also a sequential process based on Zr/Cu catalysis for providing access to enamines from terminal alkynes (Table 8, entry 8; Scheme 63).^192^ The process involves initially the Zr-catalyzed reaction of alkyne with pinacolborane (H-Bpin) and the subsequent Cu-catalyzed electrophilic amination using *O*-benzoylhydroxylamines. Overall, the reaction gives rise to terminal enamines by formal regioselective anti-Markovnikov hydroamination of terminal aryl alkynes under mild conditions (10 mol % Cu(OAc)_2_·H_2_O, 10–20 mol % ligand, 2.0 equiv of *t*-BuOLi, THF, rt, 4 h).

#### 4.1.3 Palladium/Ruthenium, Palladium/Iridium

**Scheme 64:**
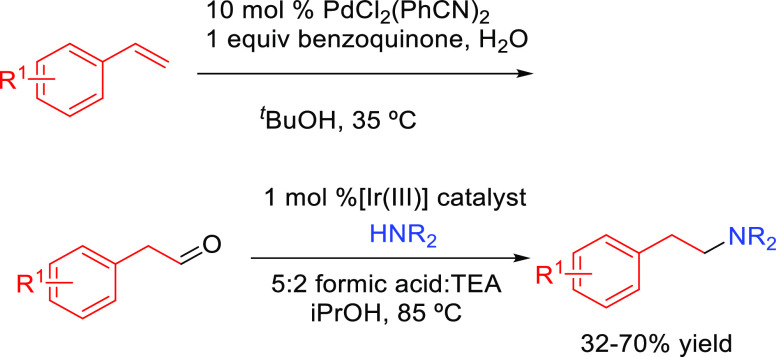
Formal Anti-Markovnikov Hydroamination of Terminal Alkenes Mediated by the Formation of an Aldehyde and Its Subsequent Amination

The formal anti-Markovnikov hydroamination of terminal olefins using a combination of Pd/Ru or Ir catalysts was developed by Grubbs and co-workers in 2014 (Table 8, entry 3).^193^ They developed a one-pot, two-step Wacker oxidation/transfer hydrogenative reductive amination approach (Scheme 64). Thus, the first step is the oxidation of the olefins to produce the aldehyde (avoiding the ketone). This is the key step to accomplish the desired regioselectivity; they benefit from the previously developed aldehyde-selective Wacker oxidation reaction of olefin (Pd(II)-based catalyst (10 mol % [PdCl_2_(PhCN)_2_], 85 °C)).^194^ The next step involves the reaction of the aldehyde with the amine to produce the imine. The imine product is reduced to the amine by an Ir(III) catalyst [iridacycle·OMe(chloro(pentamethylcyclopentadienyl){5-methoxy-2-{1-[(4-methoxyphenyl)imino-κN]ethyl}phenyl-κC}iridium(III)] with a 5:2 formic acid:triethylamine (TEA) azeotropic mixture as the hydride source. Overall, the process produces the formal anti-Markovnikov hydroamination of terminal alkenes.

**Table 8:**
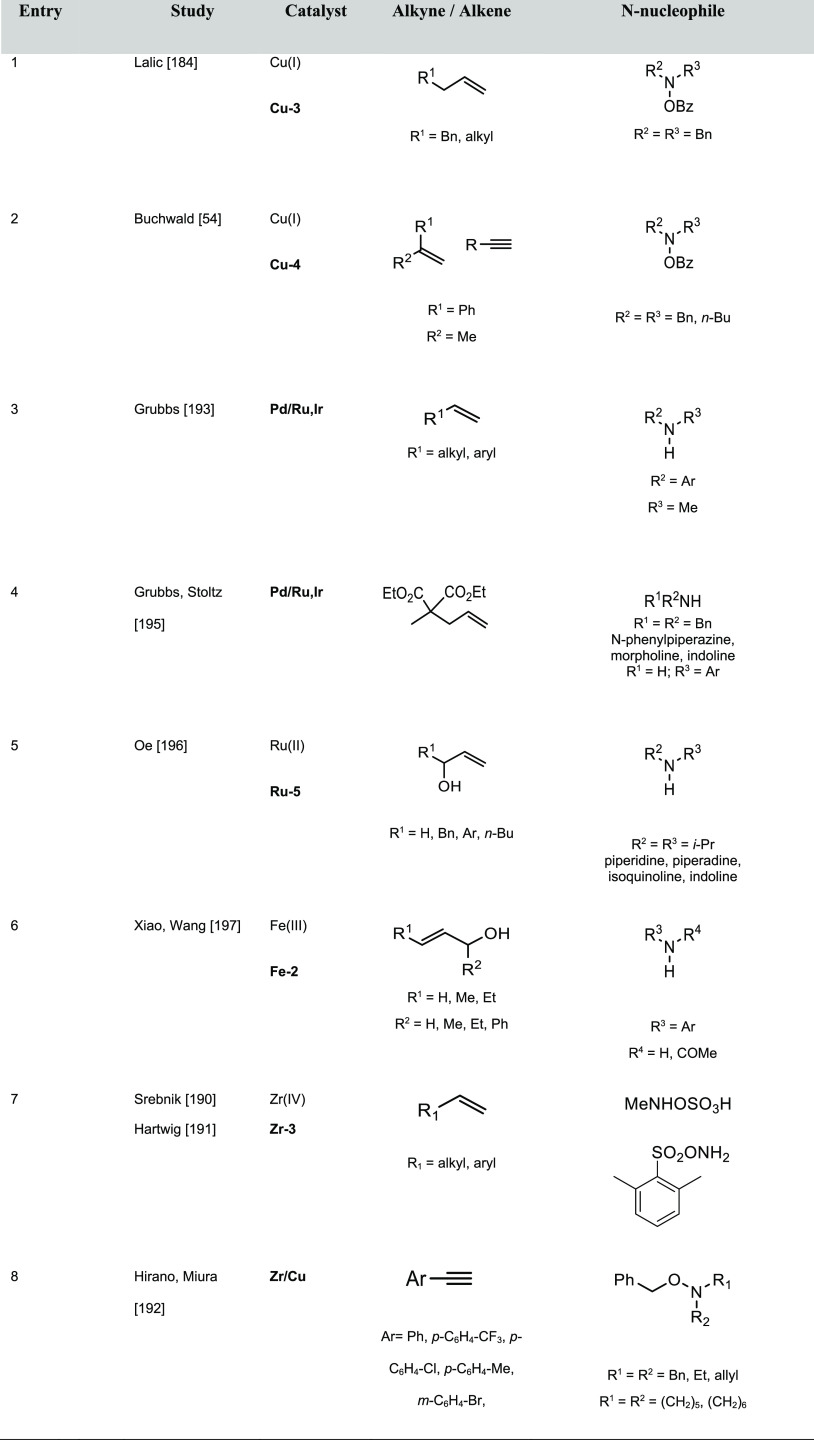
Nature of the C–C Multiple Bond Reactant and *N*-Nucleophile for the Formal Catalyzed Intermolecular Hydroaminations with Anti-Markovnikov Regioselectivity

Based on a related strategy Stoltz, Grubbs, and co-workers succeeded in the hydroamination of sterically hindered terminal alkenes (12 mol % PdCl_2_(PhCN)_2_, 12 mol % CuCl_2_·2H_2_O, 6 mol % AgNO_2_, 15:1 *t*-BuOH/CH_3_NO_2_, 23 °C) in moderate to high yields (Table 8, entry 4).^195^ Upon full conversion of the unsaturated compound under Wacker oxidation conditions, the aldehyde is formed. After filtration through a pad of silica followed by treatment of the residue with NaBH(OAc)_3_ and the amine, the reductive amination product is obtained. Tertiary and secondary amines and aliphatic and aromatic amines were efficiently obtained in almost quantitative yields.

#### 4.1.4 Ruthenium

In 2015, Oe and co-workers described a formal anti-Markovnikov hydroamination of allylic alcohols using a Ru complex as catalyst (2 mol % Ru, 2.2 mol % ligand, 2-propanol, 3 mol % *t*-BuOK, 70 °C, 22 h), namely (RuClH(CO)(PPh_3_)_3_/2,6-bis(*n*-butyliminomethyl)pyridine), **Ru-5** (Table 8, entry 5).^196^ The strategy developed by the authors is described as a “hydrogen borrowing” process consisting in three reactions, as discussed in the next subsection. The Ru catalyst was involved in the hydrogen borrowing process by taking and returning a formal H_2_ molecule from and to the alcohol group. Xiao, Wang, and co-workers have also employed the hydrogen borrowing strategy for the formal anti-Markovnikov hydroamination of allylic alcohols to give racemic γ-amino alcohols (1 mol % catalyst, 2 mol % NaHBEt_3_, 20 mol % K_3_PO_4_, cyclohexane, 80 °C, 12–24 h), based on a pincer Fe-PNP catalyst, **Fe-2** (Table 8, entry 6).^197^ Very recently, they also developed a chiral version of the process based on Noyori type Ru catalysts which afforded chiral γ-amino alcohols in good yields and excellent enantioselectivities under mild conditions (1 mol % catalyst, 1.5 equiv K_3_PO_4_, toluene, 30 °C, 12–72 h).^198^

### 4.2 Mechanistic Details for Formal Anti-Markovnikov Processes

The reaction mechanisms for the formal hydroamination processes are grouped in two different types, according to whether they involve a sequential multistep process or a single catalytic reaction in their overall processes.

#### 4.2.1 Sequential, Multistep Processes

This subsection collects the procedures in which the initial catalytic reaction involves attaching some other functional groups in an anti-Markovnikov fashion to the unsaturated substrate and then converting the resulting intermediate into the corresponding amine.

##### 4.2.1.1 Hydroboration/Electrophilic Amination

**Scheme 65:**
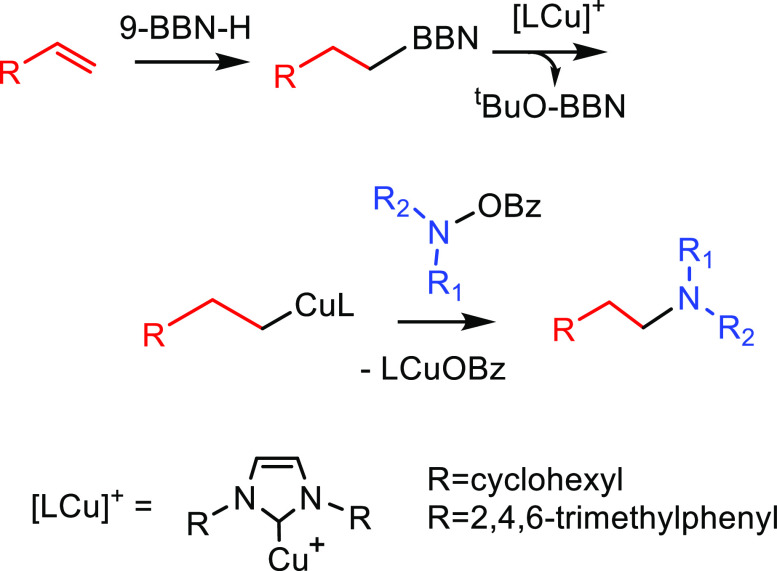
Proposed Reaction Steps for Formation of Organoborane from Alkene and the Subsequent Cu-Catalyzed Amination of Organoborane Species^184,199^

The Cu-catalyzed hydroamination of alkenes described by Lalic and co-workers can be included in the group of hydroboration/electrophilic amination processes (Table 8, entry 1).^184^ The reaction takes place in two steps: initial formation of an organoborane species and its subsequent replacement by the amine to produce the hydroaminated product. In this method the first part corresponds to an uncatalyzed hydroboration of the alkene. The second part, the one accelerated by a Cu-based catalyst, is proposed to proceed through transmetalation from boron to copper to produce an organocopper intermediate.^199^ Such an intermediate undertakes the amination with hydroxylamine *O*-benzoate reagent to yield the linear amine (Scheme 65). The mechanism was experimentally supported by testing the stoichiometric reactivity of an alkylcopper species (IMesCuEt) with amines, showing that the electrophilic amination was viable, even though it does not strictly correspond to an arene-coordinated substrate.

**Scheme 66:**
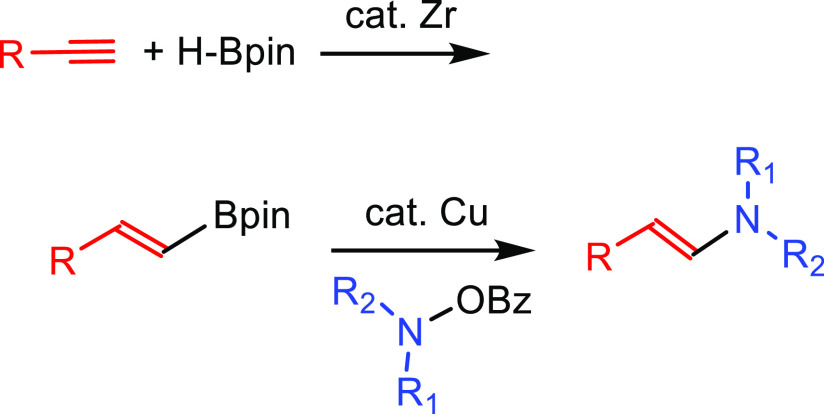
Proposed Sequential Steps for Zr/Cu-Catalyzed Formal Anti-Markovnikov Hydroamination of Alkynes^192^

The formal hydroamination of terminal alkynes was described by Hirano, Miura, and co-workers (Table 8, entry 8).^192^ The process involves Zr-catalyzed hydroboration with pinacolborane to produce the *E*-isomer of the alkenylborane species. Then, a Cu-catalyzed electrophilic amination with *O*-benzoylhydroxylamines leads to the corresponding regioselective enamines under very mild conditions. The regioselectivity is defined at the formation of the alkenylborane intermediate, because the hydroboration produces the *E*-isomer. The amination keeps the regioselectivity, generating a formal anti-Markovnikov hydroamination (Scheme 66).

##### 4.2.1.2 Oxidation/Reductive Amination

**Scheme 67:**
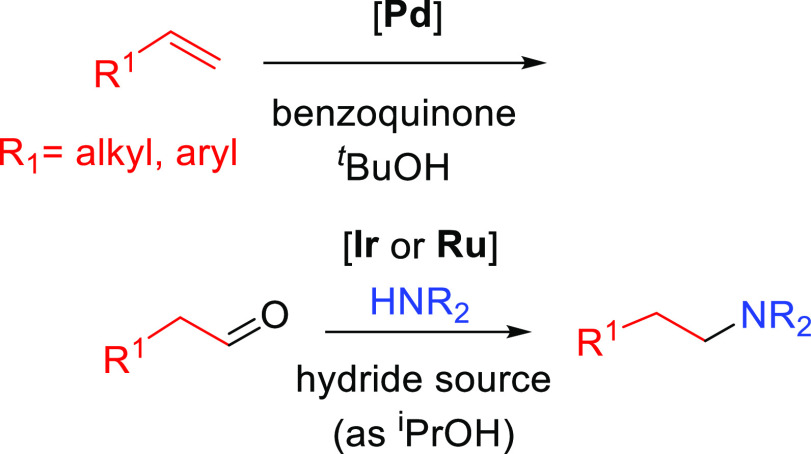
General Scheme for the Oxidation – Reductive Amination Process to Produce Anti-Markovnikov Hydroamination^193^

Grubbs and co-workers developed the formal hydroamination of terminal alkenes by means of an oxidation–reductive amination process (Table 8, entry 3; Scheme 67).^193^ For the first reaction, they employed the aldehyde-selective Wacker oxidation.^194^ Depending on the reaction conditions, the selectivity was very high.^200,201^ Next, by the addition of an amine and subsequent reaction with the aldehyde, an imine/iminium intermediate was formed. This species could be reduced in a subsequent step in the presence of a transfer hydrogenation catalyst (M = Ru or Ir) and an appropriate hydrogen source (*tert*-butanol). To address chemoselectivity issues in the reduction step, the authors replaced Shvo’s catalyst by a commercially available Ir complex that is more selective for transfer hydrogenative reduction of imines in the presence of carbonyls. Note that a one-pot methodology is beneficial over a two-pot one because, besides producing the direct formation of the linear amine, it avoids the isolation of the unstable aldehyde. A similar mechanism applies for the related process published by Grubbs, Stoltz, and co-workers (Table 8, entry 4).^195^

#### 4.2.2 Single Catalytic Reaction

This section gathers those processes where the developed formal hydroamination does not involve the conversion of the unsaturated reactant into another functionalized group; instead, the hydroamination is accomplished in a single catalytic reaction.

##### 4.2.2.1 Hydrometalation/Electrophilic Amination

The hydroamination of alkenes based on CuH catalysts can be classified within the hydrometalation/electrophilic amination processes.^185−188^ This process was conceived based on the following approach: the hydrogen atom is delivered from a hydridic source, the CuH species, while the amino product is obtained from its reaction with an electrophilic aminating reagent. The mechanistic proposals for the Markovnikov addition found in the literature^53^ were computationally evaluated by Tobisch (DFT calculations with the PWPB95-D3 functional and Def2-TZVP basis set).^202^ The proposed mechanism for this transformation for alkenes involves a Cu(I) alkoxide species (*t*-BuO-CuL, L = biphosphine ligand) that is generated under reaction conditions; it undergoes transmetalation with the hydrosilane to form the key CuH complex. The catalytically active copper hydride is in a rapid equilibrium with the dimer, in favor of the latter. The alkene reacts with the CuH intermediate to form hydrocuprated species; this step can introduce chirality by using a chiral biphosphine ligand. Importantly, the regioselectivity is also introduced at this step. Next, umpolung electrophilic amidation of the nucleophile (hydrocuprated intermediate) with a dioxazolone electrophile generates a copper amidate. The computational evaluation revealed that this process comprises an oxidative Cu=N coupling with the extrusion of CO_2_ (coming from the *N*-nucleophile reactant) and irreversible reductive elimination.^202^ Finally, the copper(I) amidate undergoes a nucleophilic attack at the hydrosilane followed by a slower hydrogen atom transfer. The reaction mechanism analyzed gives Markovnikov regioselectivity. Note that this particular mechanism only applies to the hydroamidation reaction using dioxazolones. The vast majority of CuH-catalyzed hydroamination reactions do not involve C=N intermediates and use hydroxylamine esters as the electrophilic partner.

**Scheme 68:**
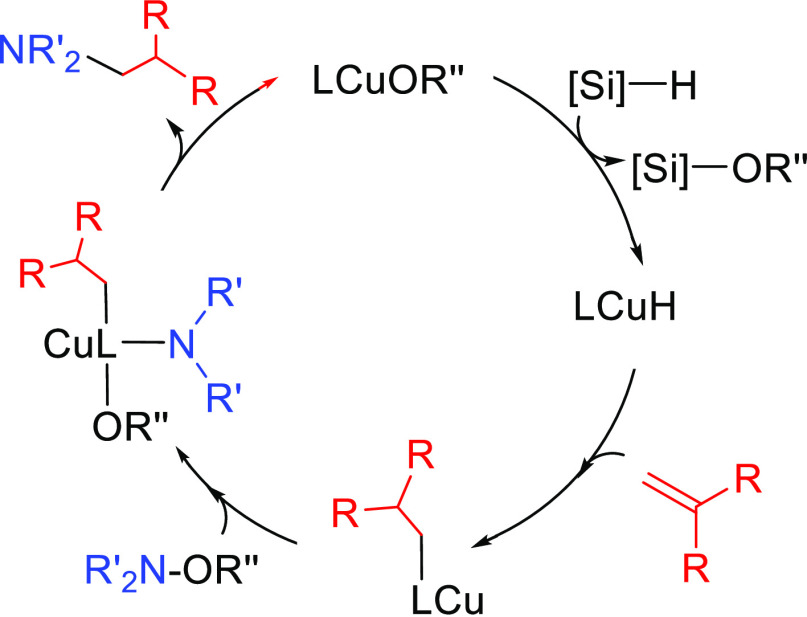
Proposed Reaction Mechanism for CuH-Catalyzed Hydroamination of 1,1-Disubstituted Alkenes with Dioxazolones^188^

For the cases where the anti-Markovnikov product is obtained, i.e., 1,1-substitued alkenes, the divergence in regioselectivity is introduced at the hydrocupration of the alkene step (Table 8, entry 2). Such regiodiscrimination has been attributed to the lack of an electronic advantage for Cu to form the secondary alkyl–Cu bond (conversely to the case of the Markovnikov hydroamination of styrenes), along with the formation of the less sterically crowded terminal copper intermediate (Scheme 68).^188^ In a recent computational evaluation using energy decomposition analysis (EDA), Lv, Lu, and co-workers pointed out that, at the transition states, Pauli repulsion controls the regioselectivity for terminal and internal alkenes.^203^ For 1,1-dialkyl terminal alkenes it is controlled by the repulsive electrostatic interactions between both the negatively charged hydride and the terminal carbon.

**Scheme 69:**
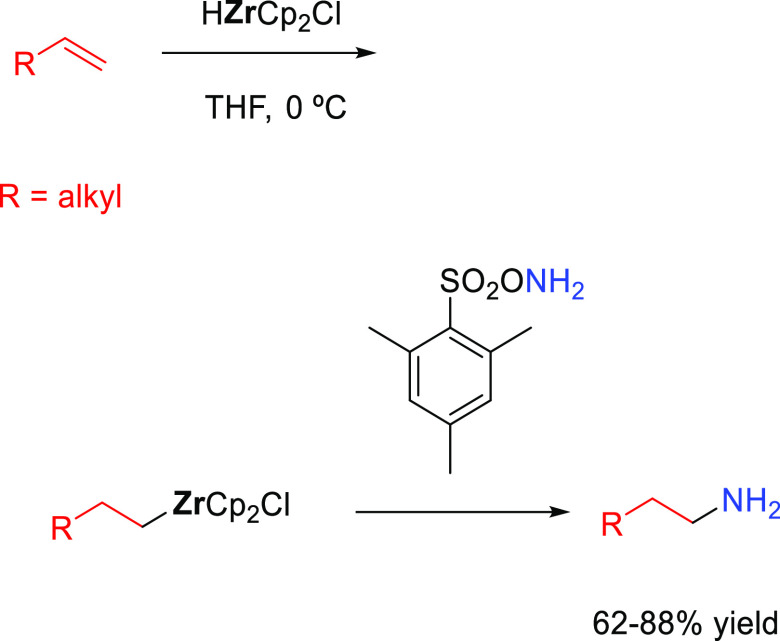
Reaction Sequence for the Amination of Zirconocene Alkyl Chlorides with *O*-(Mesitylsulfonyl)hydroxylamine

**Scheme 70:**
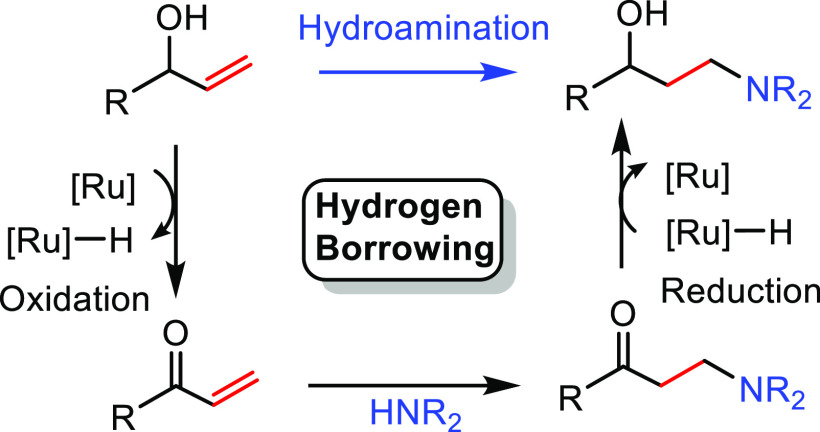
General Scheme for the Hydroamination of an Allylic Alcohol by Means of the Hydrogen Borrowing Mechanism^196^

**Figure 19:**
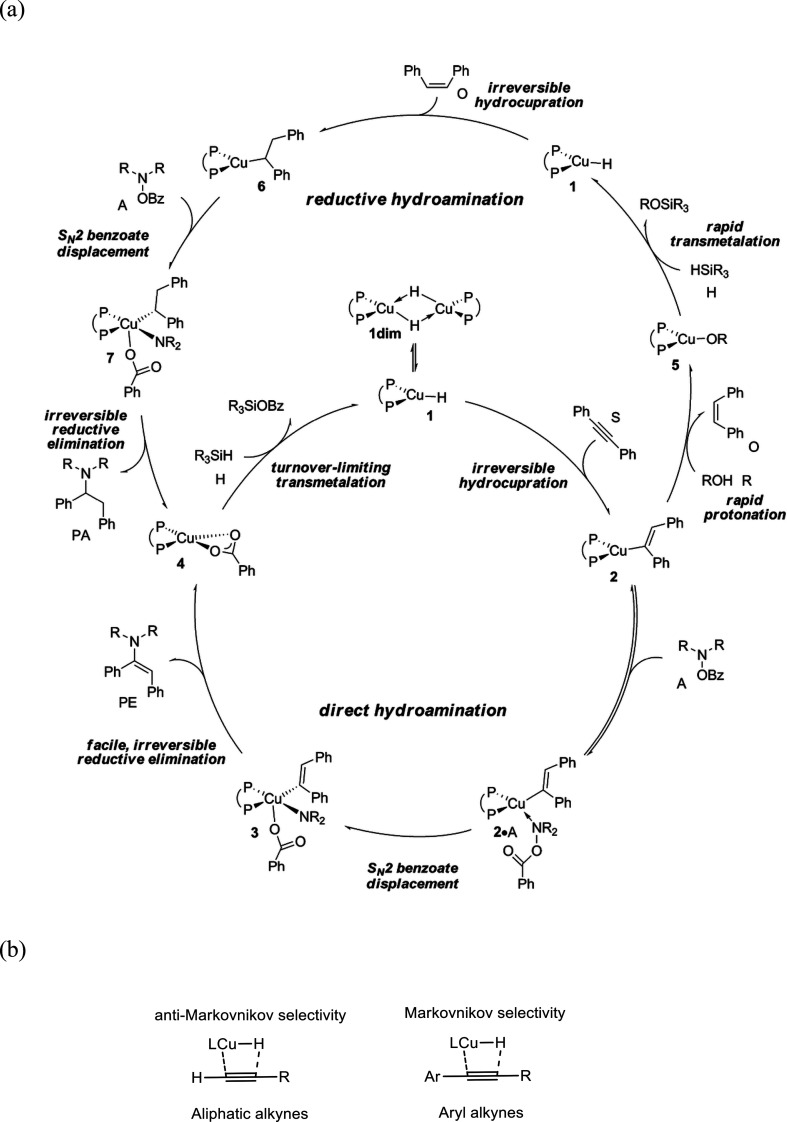
(a) Computationally evaluated mechanism^204^ for hydroamination of arylalkynes in the presence (reductive amination) and absence (direct hydroamination) of proton sources. Intermediate **2** is crucial for the chemo- and regioselectivity of the process. Reproduced from ref (204). (b) Formal representation of hydrocupration of arylalkynes (giving Markovnikov addition) and aliphatic alkynes (anti-Markovnikov addition).^189^

The related CuH-catalyzed hydroamination of alkynes may give rise to two different products, alkylamine and enamine, mostly depending on the reaction conditions (presence and absence of protonating agent, ROH, respectively).^189^ The proposed mechanisms for both alkyne hydroamination processes (enamine and alkylamine formation) were also computationally analyzed by Tobisch (DFT calculations with the PW6B95-D3 functional and def2-TZVP basis set) (Figure 19a).^204^ The insertion of the alkyne into the CuH species forms a vinylcopper intermediate. This step defines the regioselectivity of the process, as shown in Figure 19b; aryl alkynes give rise to Markovnikov additions, whereas aliphatic alkynes give rise to anti-Markovnikov selectivity. If there is no proton source available, the alkenylcopper intermediate reacts with the hydroxylamine *O*-benzoate by means of an oxidative addition to give the benzoate and the amide ligands coordinated to the metal center. The reductive elimination of the vinyl and amide ligands produces the hydroamination product. Transmetalation of the Cu(I)-benzoate intermediate with silane closes the catalytic cycle, regenerating the copper hydride species. In the presence of a proton source (alcohol additive), the reaction from the alkenylcopper intermediate gives rise to the final alkylamine product (reductive hydroamination; Figure 19). The reaction mechanism for the hydroamination of alkynes is more complex than that for alkenes, among other things because reaction conditions guide the regioselectivity toward the formation of alkylamines or enamines in the presence and absence of proton sources (ROH), respectively. Assuming that the reaction mechanism for terminal alkynes is similar to that shown in Figure 19, the chemo- and regioselectivity of the process are also decided at the hydrocupration step.

In the initial work of Srebnik and Zheng,^190^ then extended by Strom and Hartwig,^191^ they developed the conversion of alkenes to terminal amines by employing a Cp_2_ZrHCl catalyst in a one-pot reaction. In this process there is an initial hydrometalation of the alkene and a subsequent conversion of the alkylzirconium compound into the corresponding primary or secondary amine (Scheme 69).

##### 4.2.2.2 “Hydrogen Borrowing” Process

Oe and co-workers developed a methodology described as a “hydrogen borrowing” process (Table 8, entry 5).^196^ This strategy consists of the combination of three different reactions: (i) an initial oxidation to generate an α,β-unsaturated carbonyl compound, (ii) a 1,4-conjugate addition giving rise to a β-amino carbonyl compound, and finally, (iii) the reduction to regenerate the alcohol (Scheme 70). The Ru catalyst is involved in the hydrogen borrowing process by taking and returning a formal H_2_ molecule from and to the alcohol group (reactions (i) and (iii) of the process). Generation of the α,β-unsaturated carbonyl compound facilitates the 1,4-conjugate addition of the amine giving rise to the formal anti-Markovnikov hydroamination product. In order to support this mechanistic proposal, the reaction substituting the -OH of the allylic alcohol by -OMe was tested, giving no reaction products. Thus, this is an indication that the overall process goes via formation of an α,β-unsaturated carbonyl intermediate. The results obtained by Xiao, Wang, and co-workers for hydroamination of allylic alcohols can be explained in a similar way (Table 8, entry 6).^197^

## 5 New Directions in Anti-Markovnikov Hydroamination Processes

Anti-Markovnikov hydroaminations described in the preceding sections were achieved by means of metal catalysis. This has been for many years the usual approach for anti-Markovnikov hydroamination processes. However, in the last few years, fostered by the compelling search for metal-free catalyzed synthetic processes, some reports have appeared under metal-free conditions or nondirect transition metal-catalyzed anti-Markovnikov reactions. Among them, a wide variety entail radical mechanisms and will be described in the first subsection. The next subsection collects other recent anti-Markovnikov hydroamination processes. Finally, the last subsection reports recently developed heterogeneous processes.

### 5.1 Radical Hydroamination

Efficient radical pathways for the most challenging intermolecular anti-Markovnikov hydroamination of unactivated alkenes have been devised in the last few years. Such reactions involve the generation of either an *N*-centered radical^205^ or a C-centered cation radical from amine or alkene substrates by a single electron oxidation. Thus, a radical is present in the C–N bond formation step. Usually, oxidation of the substrates is performed by means of a photooxidant, although in some specific cases thermal radical initiation can happen. A hydrogen atom transfer (HAT) must take place as the final step of the process; therefore, a hydrogen atom donor is also required. Table 9 collects the reported radical-mediated intermolecular anti-Markovnikov hydroaminations.

**Figure 20:**
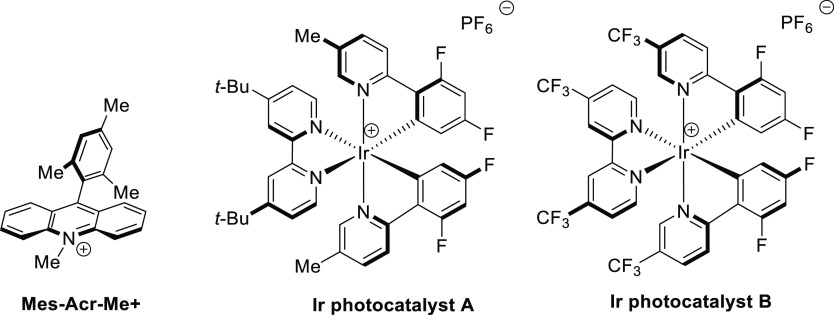
Photooxidants employed in anti-Markovnikov alkene hydroaminations.

Nicewicz described the catalytic hydroamination of substituted styrenes as well as aliphatic alkenes by using an organocatalytic photoredox system.^206,207^ Both trifluoromethanesulfonamide (TfNH_2_) and heterocyclic amines can be employed as *N*-nucleophiles (Table 9, entries 1 and 2). Irradiation of the substrates with visible light in the presence of catalytic quantities of 9-mesityl-10-methylacridinium tetrafluoroborate (**Mes-Acr-Me+**, Figure 20) and thiophenol or phenyl disulfide as cocatalyst affords the amine coupled with complete anti-Markovnikov regioselectivity. Shu and co-workers described a related process using a similar catalyst (**Mes-Acr-Ph+**, Figure 20) but employing ammonium carbonate as ammonia surrogate (Table 9, entry 3).^208^

**Table 9:**
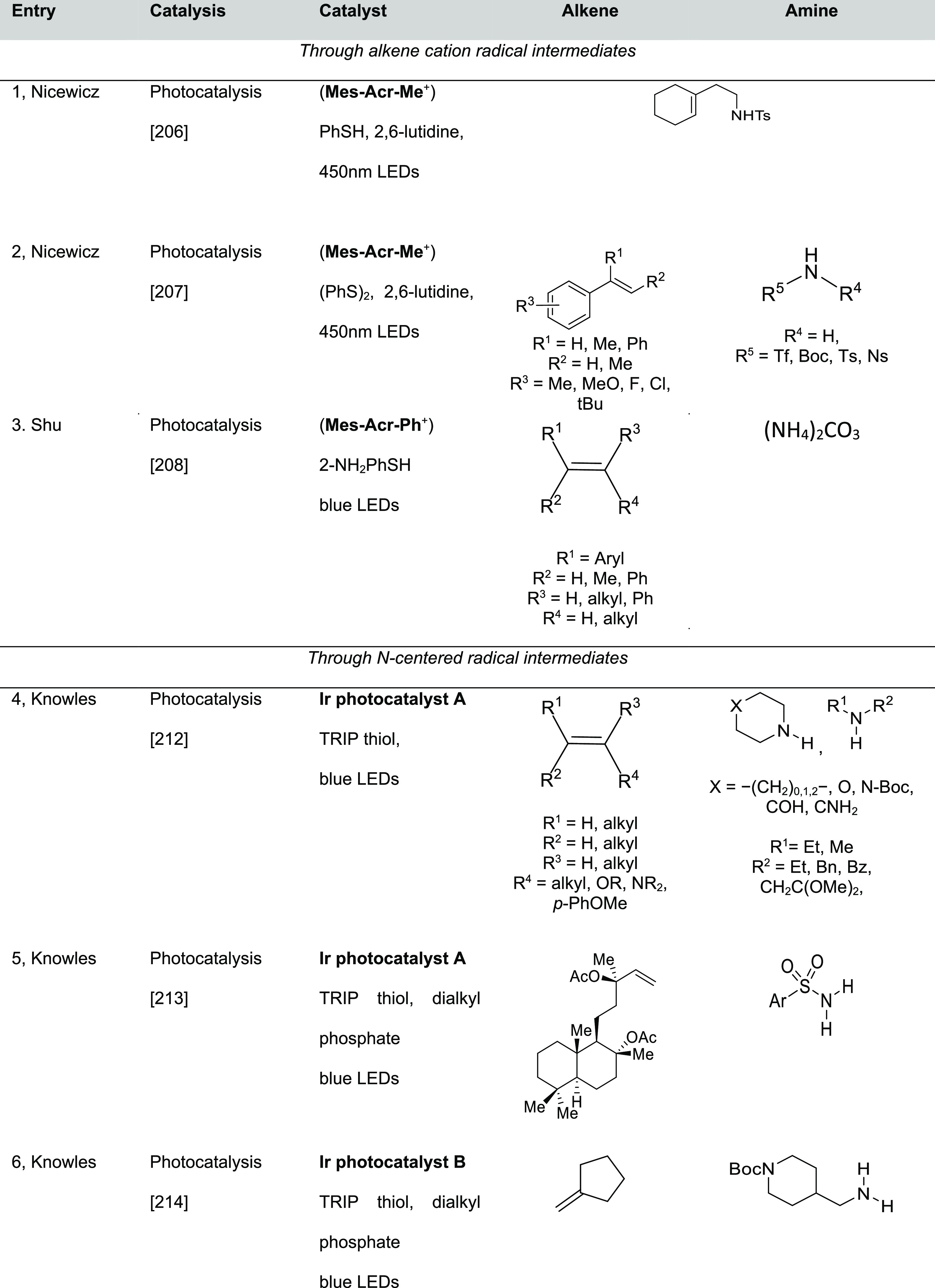

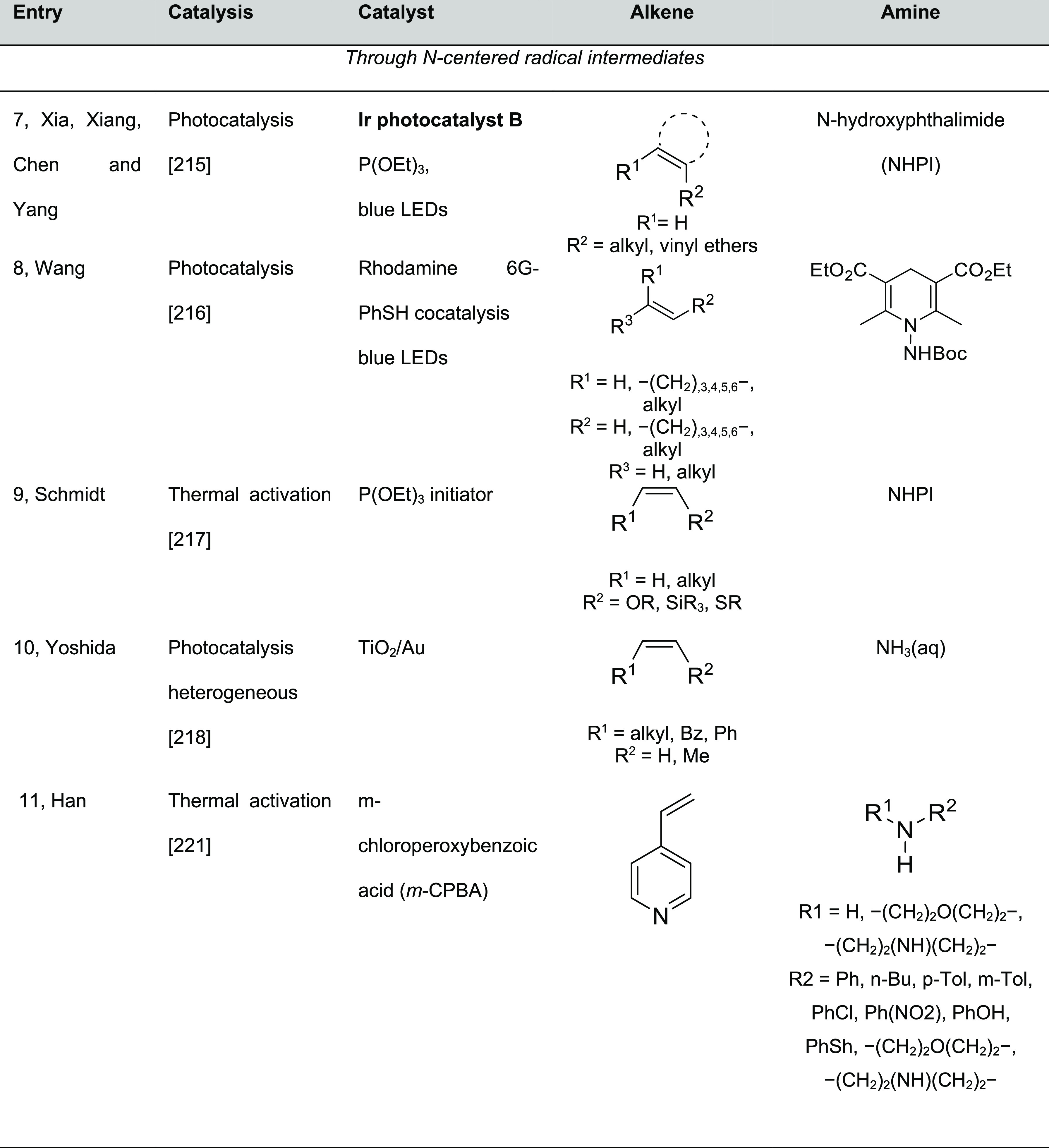
Experimentally Characterized Radical Mediated Intermolecular Alkene Hydroaminations with Anti-Markovnikov Regioselectivity

**Scheme 71:**
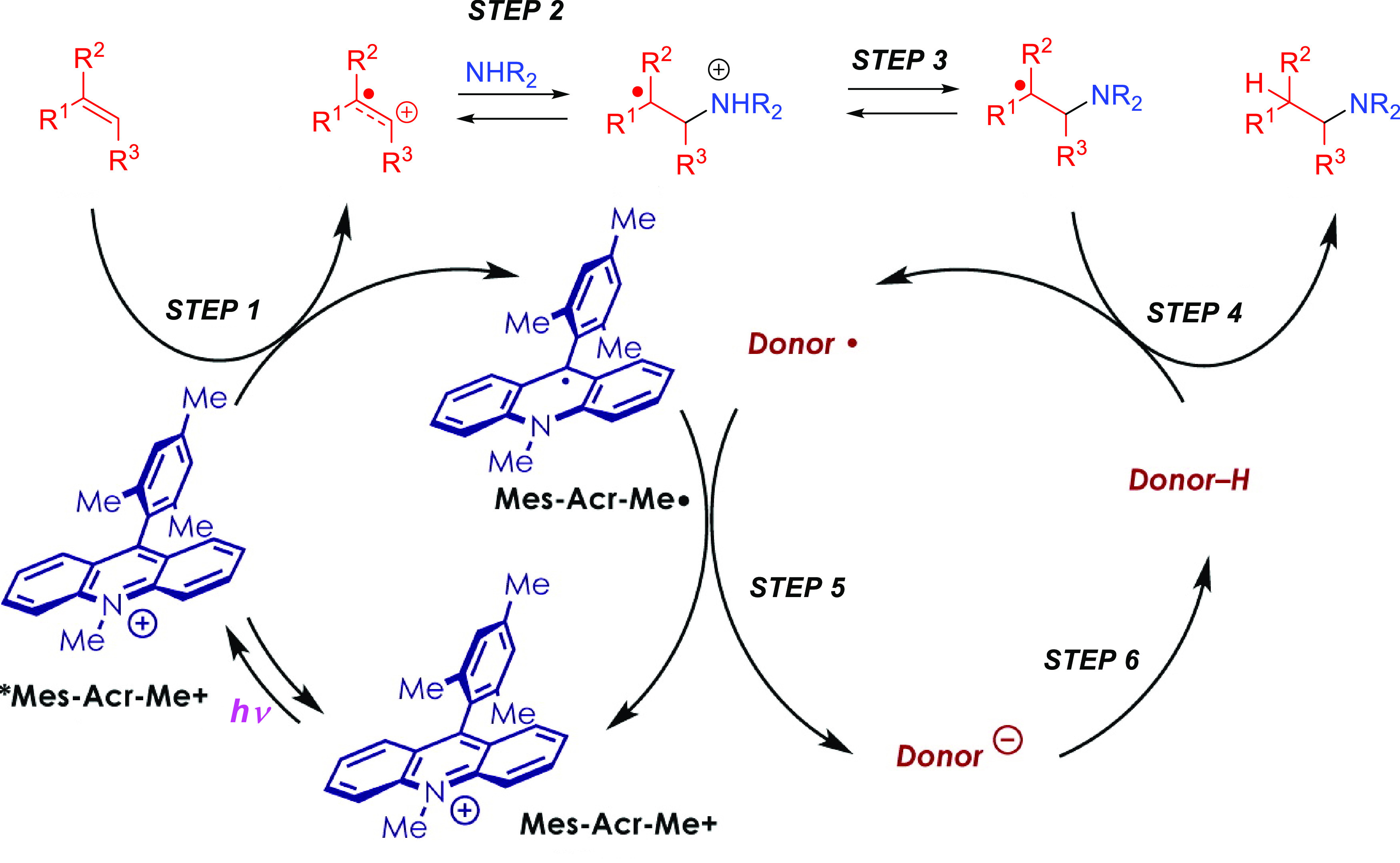
Proposed General Mechanism for Catalytic Anti-Markovnikov Alkene Hydroamination via Acridinium Phtoredox Catalysis^210^^,^ Adapted with permission from Acc. Chem. Res.2016, 49, 1997–20062758881810.1021/acs.accounts.6b00304. Copyright 2020 American Chemical Society.

Thorough mechanistic studies using spectroscopic techniques were able to detect alkene cation radical intermediates and confirmed that alkenes are oxidized by the acridinyl radical.^209^ The system works as a dual organic catalyst consisting of an acridinium photooxidant and a redox-active hydrogen atom donor. Scheme 71 depicts the proposed mechanism for this process.

**Scheme 72:**
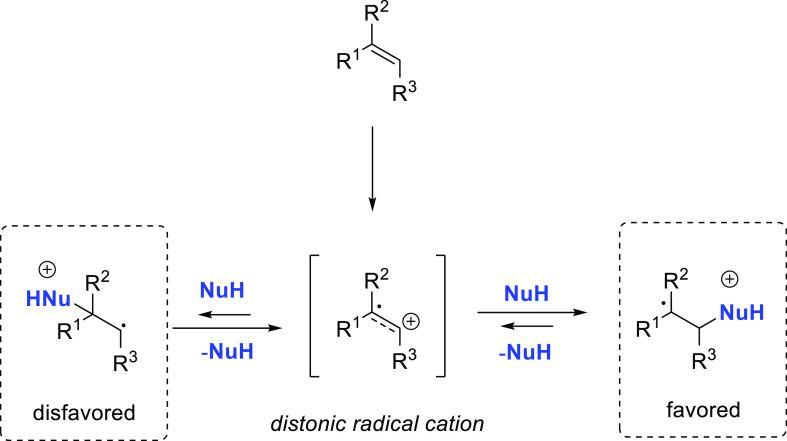
Regioselectivity in Addition of Polar Nucleophiles to Olefin Cation Radicals

Single electron transfer from the alkene to the excited acridinium photocatalyst, *Mes-Acr-Me^**+**^, forms the reactive alkene cation radical intermediate. The C–N bond is formed by nucleophilic addition of the amine to this cation radical. Deprotonation of the resulting intermediate affords a neutral radical, from which subsequent hydrogen atom transfer (HAT) furnishes the anti-Markovnikov hydroaminated adduct.^210^ In this mechanistic scheme, the anti-Markovnikov regioselectivity of addition is defined by the stability of the distonic radical cation (Scheme 72). Of the two competing distonic cation radicals arising from the *N*-nucleophile addition, the more substituted radical is likely more highly populated.^211^

**Scheme 73:**
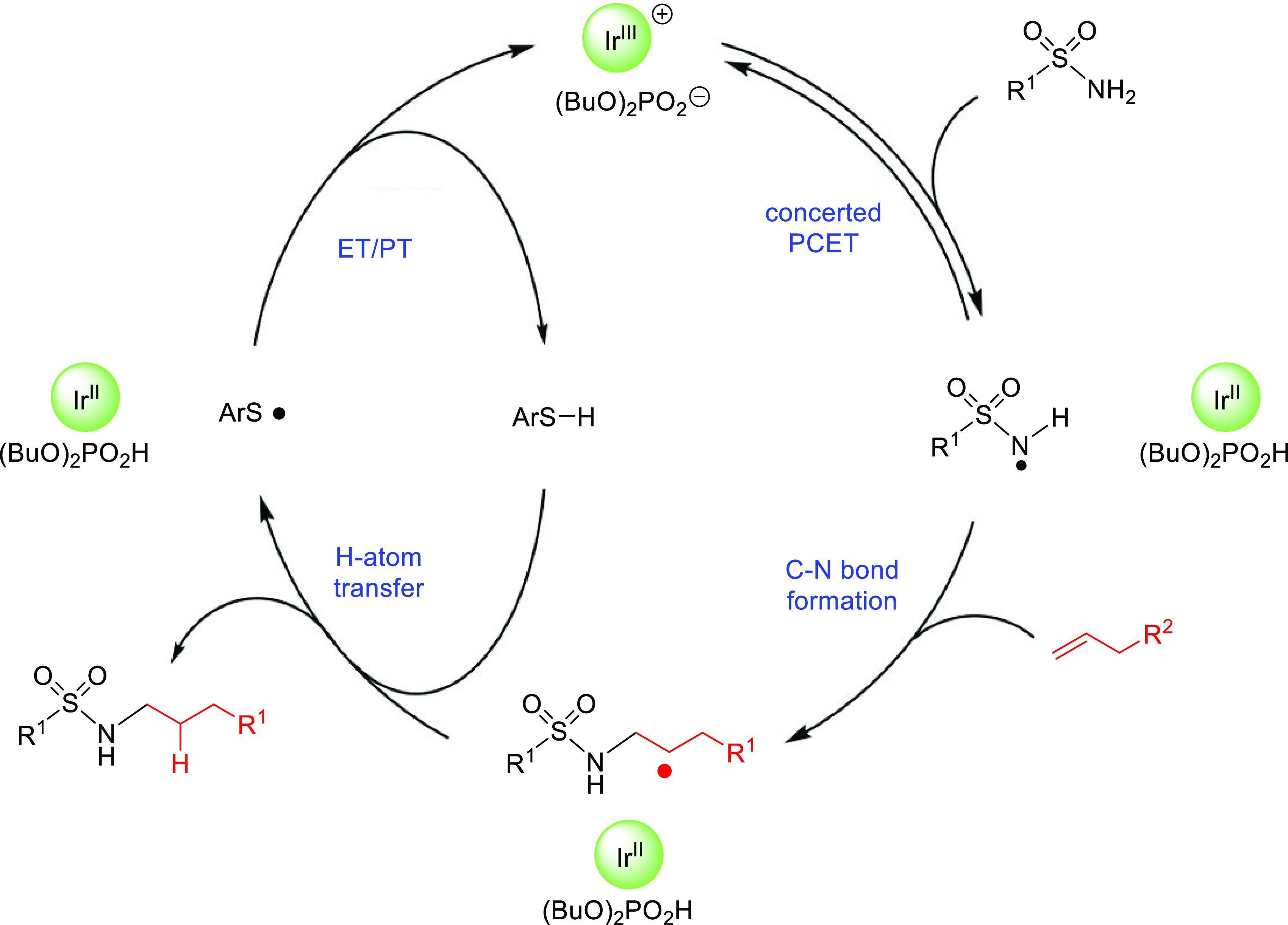
Proposed Mechanism for the Photocatalytic Anti-Markovnikov Hydroamination of Unactivated Alkenes with Sulfonamides

An alternative radical pathway entails photooxidation of the amine substrate to form an aminium radical cation intermediate. With this approach Knowles reported efficient additions of cyclic and acyclic secondary alkyl amines to a wide range of alkyl alkenes with complete anti-Markovnikov selectivity (Table 9, entry 4).^212^ The amine is oxidized by the excited state redox catalyst, the Ir(III) complex [Ir(dF(Me)ppy)_2_(dtbbpy)]PF_6_ (Ir photocatalyst A, Figure 20). An aryl thiol cocatalyst is also participating in the reaction in a HAT step. The scope of amine substrates was further extended to sulfonamides (Table 9, entry 5).^213^ With these substrates the reaction happens through the formation of an intermediate *N*-centered sulfonamidyl radical generated through proton-coupled electron transfer (PCET) activation of the sulfonamide N–H bond. A thiol hydrogen atom donor and the dialkyl phosphate base are required for the success of the process. The proposed catalytic cycle is shown in Scheme 73. The phosphate base initially forms a hydrogen bonded complex with the sulfonamide N–H bond. The noncovalent adduct formed would participate in a concerted PCET even with the excited state of the iridium photocatalyst; this results in a formal homolysis of the N–H bond along with the formation of the sulfonamidyl radical, a key intermediate. The anti-Markovnikov regioselectivity is justified by the favored addition of the N-radical species to the olefin to furnish a new C–N bond and a vicinal carbon-centered radical.^213^ When the more oxidizing Ir(III) photocatalyst B is employed (Figure 20), primary alkyl amines can be used as *N*-nucleophiles, with high selectivities for secondary over tertiary amine products (Table 9, entry 6).^214^

**Scheme 74:**
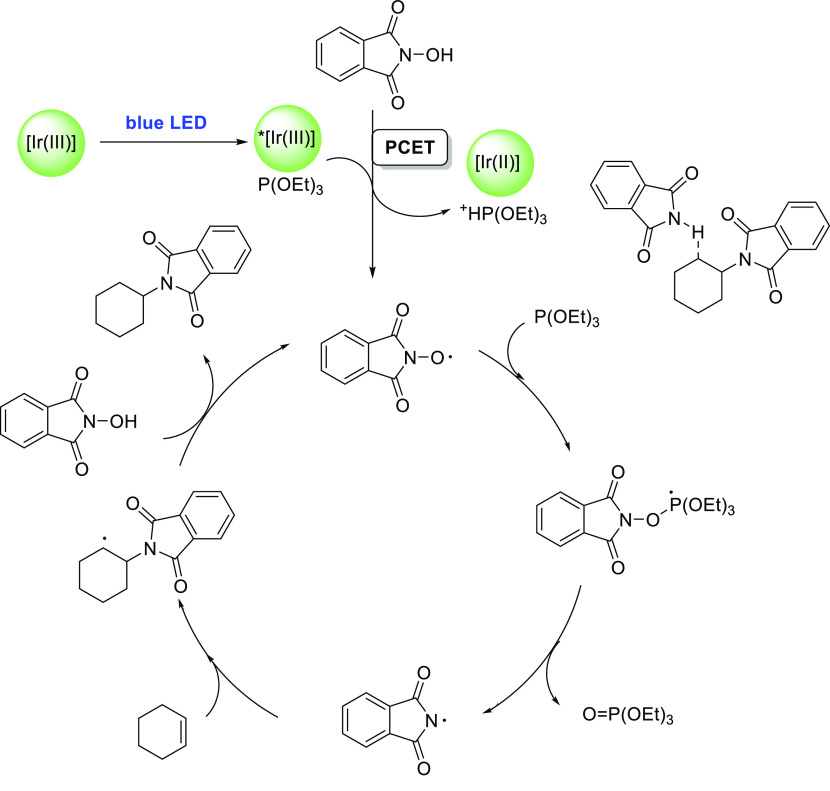
Proposed Mechanism for the Radical Mediated Anti-Markovnikov Alkene Hydroamination Using *N*-Hydroxyphthalimide and Ir(III) Photocatalyst B under Visible Light Irradiation^215^

Chen, Yang, and co-workers accomplished a phosphite-promoted (*N*-hydroxyphthalimide, NHPI) anti-Markovnikov hydroamination of alkenes with *N*-hydroxyphthalimide in good to moderate yields by using Ir(III) photocatalyst B under visible light irradiation (Table 9, entry 7).^215^ Control experiments supported a radical mechanism after showing product inhibition when using radical trapping agents. The role of *N*-hydroxyphthalimide as the hydrogen source was confirmed by using *N*-hydroxyphthalimide sodium salt, which hampered the formation of the hydroamination product. The photocatalytic excitation of [Ir(III)] to *[Ir(III)] under blue LED irradiation is followed by a proton-coupled electron transfer that enables the direct cleavage of the N–O bond in *N*-hydroxyphthalimide (PhthN–OH); this produces the *N*-centered radical intermediate and delivers O=P(OEt)_3_. The radical addition to the alkene generates the radical carbon intermediate which delivers the final product upon hydrogen transfer from *N*-hydroxyphthalimide (Scheme 74).

In a recent work, Zhao and Wang described the photocatalytic intermolecular hydroamination of unactivated alkenes with *N*-aminated dihydropyridines using a metal-free system, based on rhodamine 6G and thiophenol.^216^ Despite moderate yields being achieved, excellent anti-Markovnikov selectivity was reported after 3 h of visible light irradiation. The authors proposed a plausible mechanism based on Stern–Volmer emission quenching studies, which helped to support the generation of the amino radical cation from the oxidation of *N*-aminated dihydropyridine by an oxidizing excited state of rhodamine 6G.

**Scheme 75:**
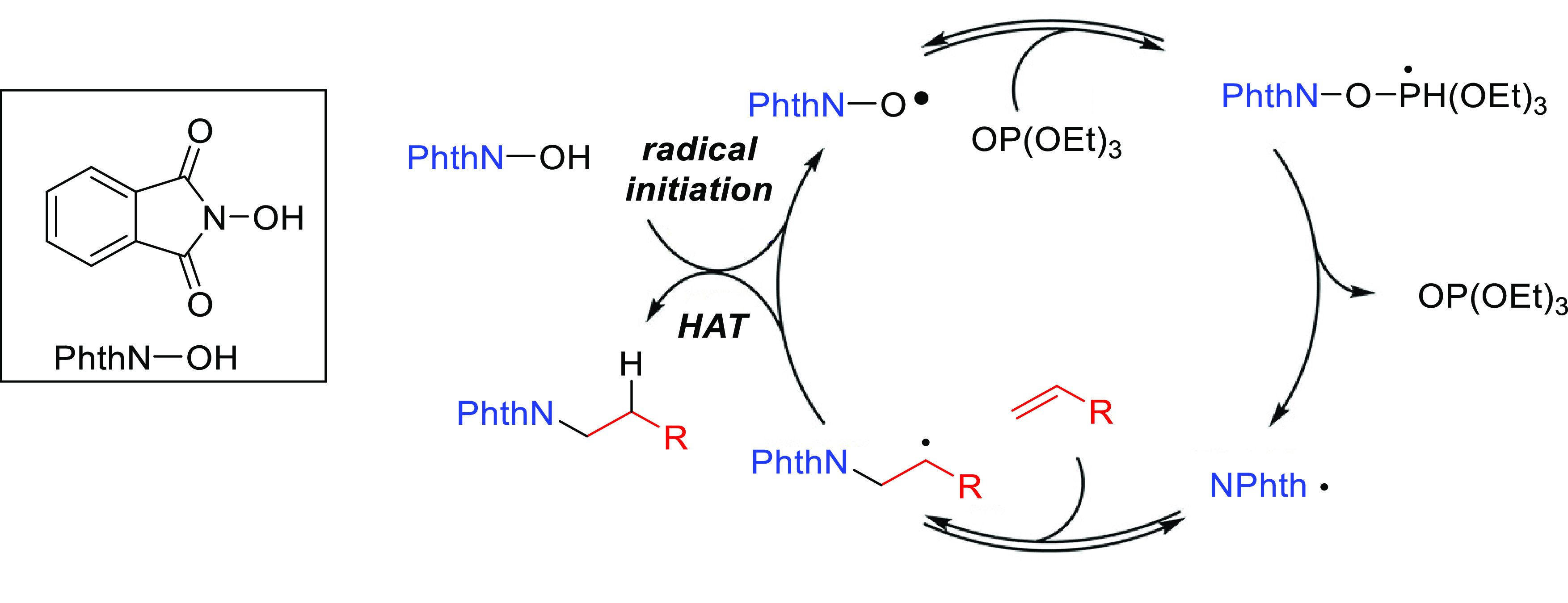
Proposed Mechanism for the Radical-Mediated Anti-Markovnikov Alkene Hydroamination Using *N*-Hydroxyphthalimide under Thermal Activation^217^

In some cases, the *N*-centered radical can be formed by thermal activation. That is the case when using *N*-hydroxyphthalimide (PhthN-OH) and triethyl phosphite (Table 9, entry 9).^217^ Mechanistic studies support that the process is mediated by a phosphite promoted radical deoxygenation of *N*-hydroxyphthalimide to afford the formation of phthalimidyl radicals. In the proposed mechanism, schematized in Scheme 75, a phthalimidyl radical PhthN^•^ is formed from the β-scission of the N–O bond of the PhthNO–phosphite adduct. The *N*-radical adds to the alkene preferentially delivering the more substituted *C*-centered radical. This alkene addition product can abstract a H atom from another molecule of *N*-hydroxyphthalimide, affording the hydroaminated product and regenerating PhthNO.^217^

Recently, Yoshida, and co-workers described the synthesis of anti-Markovnikov primary amines from alkenes using aqueous ammonia as *N*-nucleophile.^218^ Au-loaded TiO_2_ is the photocatalyst of this transformation (Table 9, entry 10). It has been proposed that a photocatalytically formed amide radical (NH_2_) takes part in the process. This radical reacts with an alkene molecule to form a radical intermediate (amide radical adduct); this species reacts with a hydrogen radical to yield an anti-Markovnikov product. The regioselectivity would be determined by the thermodynamic stability of the radical intermediate formed from an alkene and the amide radical.^218^

**Scheme 76:**
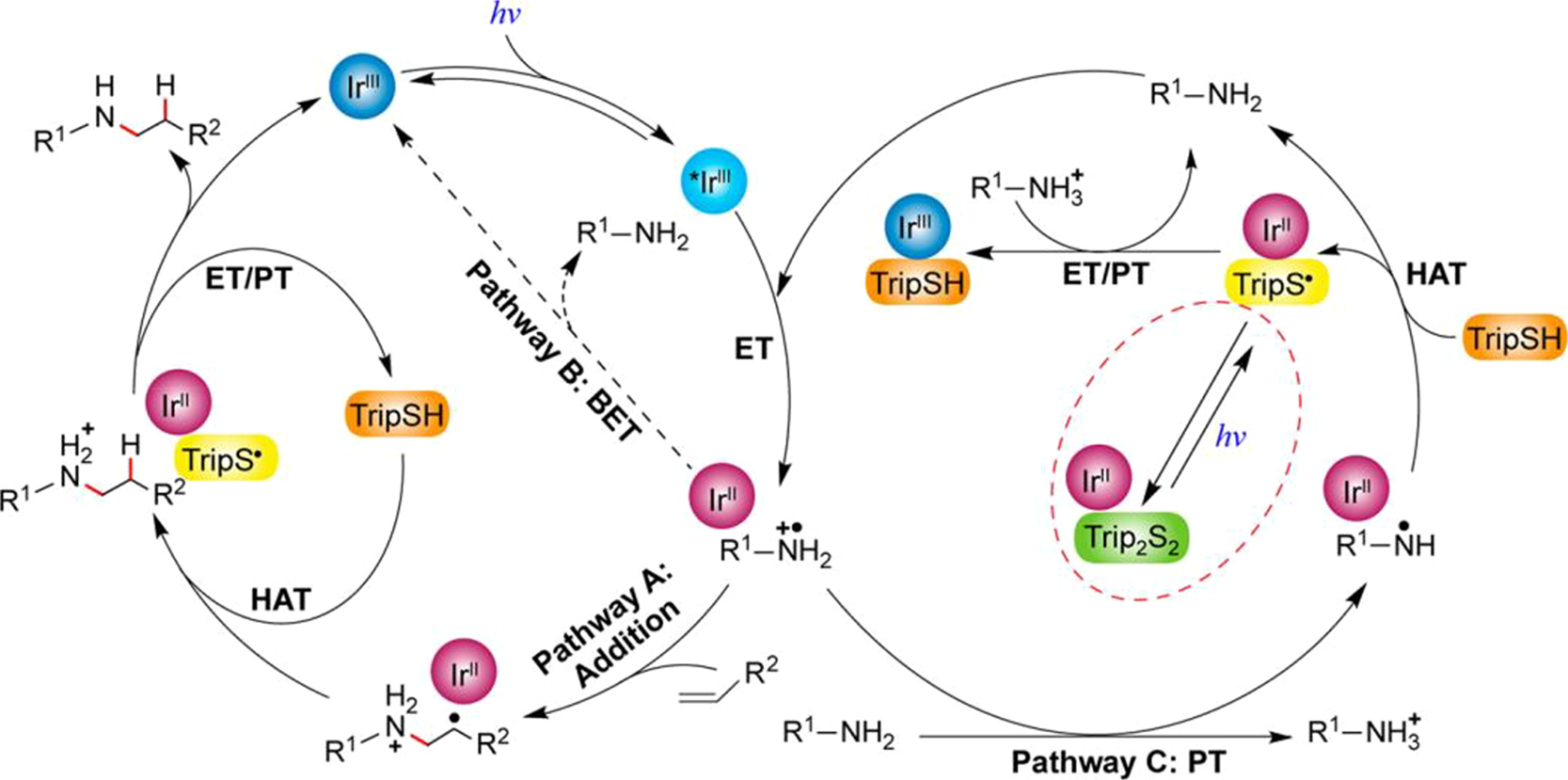
Complete Reaction Cycle for Photoredox Hydroamination ET = electron transfer; BET = back-electron transfer; PT = proton transfer; HAT = hydrogen atom transfer. Reprinted with permission from ref (219). Copyright 2021 American Chemical Society.

Knowles and Nocera used time-resolved laser kinetics measurements to unravel the reaction mechanism and the origin of the selectivity for the intermolecular anti-Markovnikov hydroamination of unactivated alkenes with primary amines with Ir photocatalyst B under blue light irradiation.^219^ The proposed mechanism, depicted in Scheme 76, starts with the excited state photocatalyst *Ir(III), which oxidizes the amine to generate Ir(II) and the aminium radical cation. The latter can react along three possible pathways: (A) addition to alkene in a productive reaction, (B) direct back-electron-transfer (BET) with Ir(II), and (C) a nonproductive cycle via deprotonation to form a neutral radical.

Regioselective anti-Markovnikov hydroamination of 1-octene with ammonia induced by nonthermal atmospheric plasma (NTAP), forming exclusively 1-octylamine, has been reported.^220^ Considering the experimental conditions generated within the NTAP gas, it is likely that a radical mechanism is operating, with ammonia being activated by electron collision.^220^ The anti-Markovnikov hydroamination of 4-vinylpyridine was obtained using aniline and several secondary amines by adding an oxidant which is presumed to have an initiating role in the mechanism (Table 9, entry 11).^221^ Several oxidants, such as *m*-chloroperoxybenzoic acid (*m*-CPBA), *N*-hydroxyphthalimide, *tert*-butanol, hydrogen peroxides, etc., were tested, with the first one giving the best yields.

### 5.2 Biocatalysis and Other Nonradical Hydroamination Processes

A different approach, yet almost unexplored, is the use of biocatalysis. Biocatalytic formal anti-Markovnikov hydroamination of aryl alkenes has been achieved via an epoxidation–isomerization–amination enzyme cascade. Cheap and nontoxic reagents (O_2_, NH_3_, and glucose) and *Escherichia coli* cells are used in the reaction for synthesis of terminal amines and alcohols in high selectivity and yield.^222^

**Scheme 77:**
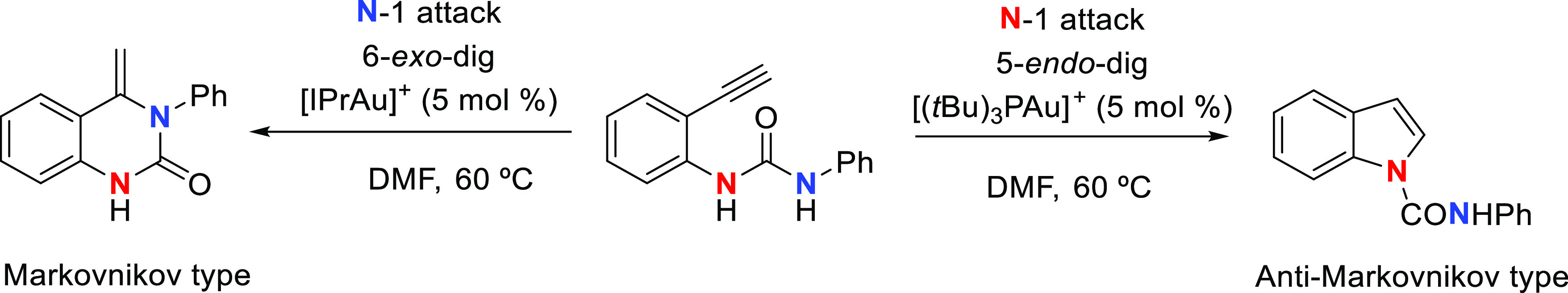
Gold(I) Catalyst Regioselectivity in the Heterocyclization of 1-(*o*-Ethynylaryl)ureas^224,225^

By engineering and evolving an artificial hydroaminase, it has been possible to control the regioselectivity in the heterocyclization of 1-(*o*-ethynylaryl)ureas. Suitable mutants generated by computational modeling and directed evolution can turn the regioselectivity from 99% in favor of the Markovnikov product to 96% in favor of the anti-Markovnikov one.^223^ As for the synthetic case (Scheme 77),^224,225^ the anti-Markovnikov pathway is favored by a dual σ,π-activation of the alkyne, performed by two Au(I) centers.

In addition to radical processes, new approaches have been devised for regioselective anti-Markovnikov hydroaminations. A metal-free organic base-catalyzed regioselective hydroamination of alkynes catalyzed by a cyclic trimeric phosphazene superbase (CTP) was reported by Wen, Li, and co-workers.^226^ An ion pair mechanism was proposed in which the [CTPBH^+^···NR_2_^–^] ion pair initially formed adds to the alkyne with an anti-Markovnikov regioselectivity. With this methodology, a wide variety of *N*-heterocyclic structures was achieved in good to excellent yields and high stereoselectivity using terminal and internal alkynes with *N*-heterocycles as nucleophiles under solvent-free conditions.

### 5.3 Heterogeneous Catalysts

The use of well-established heterogeneous catalysts has experienced a blossoming during the past decades given the environmental advantages associated with the use of these sustainable catalysts; therefore, this is not a new strategy, since it has been well-established. As mentioned in this review, several metals and metal complexes, including alkali and alkaline earth metals, early and late transition metals, and rare earths, have been efficiently used as homogeneous catalysts to promote the nucleophilic addition of amines to multiple C–C bonds; however, the development of heterogeneous catalysts for anti-Markovnikov hydroamination has been limited to a few examples.^227^ In general, solid acids containing either Bro̷nsted or Lewis acidic sites have been described to activate the alkene and facilitate the nucleophilic attack by the amine. However, in most of the cases the selectivity of the hydroamination is a limiting factor, as the diaddition product is formed along with the monoaddition anti-Markovnikov product.

**Scheme 78:**
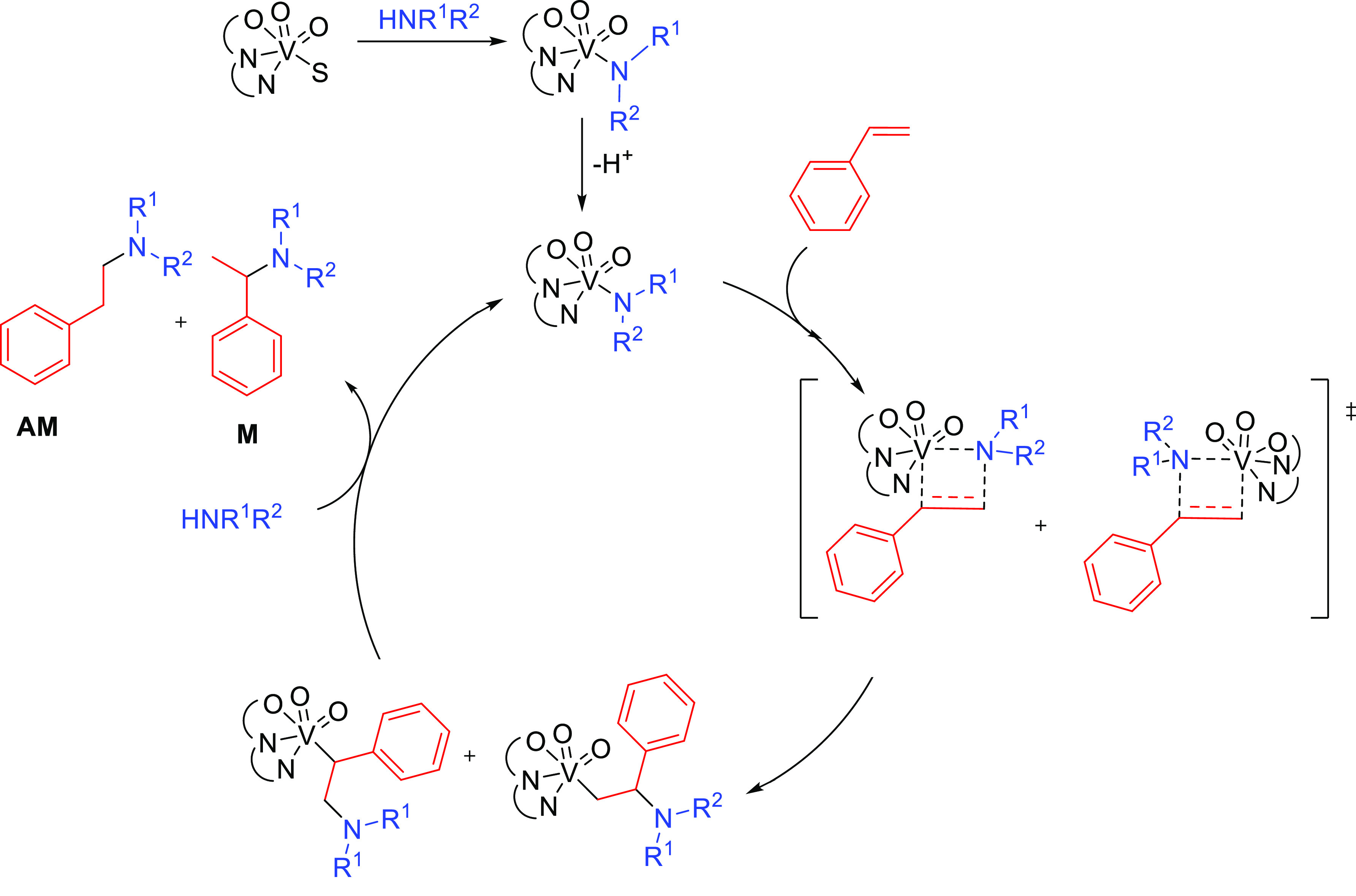
Tentative Catalytic Cycle of the Hydroamination Process Catalyzed by Polymer-Anchored Vanadium Complexes^228^

Maurya, Pessoa, and co-workers reported the hydroamination of styrene and vinyl pyridine with amines catalyzed by polymer-supported oxido and dioxido vanadium complexes.^228^ Under the optimized conditions (toluene, 90 °C, 80 min), polystyrene-bounded catalysts for the hydroamination of styrene and vinyl pyridine with aromatic and aliphatic amines afforded a mixture of two hydroaminated products in good to high yields for aromatic amines (yields for aliphatic amines up to 70%), favoring the formation of the anti-Markovnikov product (selectivity up to 90%). The authors proposed a mechanism in which the acceptor of the proton to form the amide may be one of the N-atoms of the ligand (Scheme 78). The mechanism involves amine activation by the generation of the highly nucleophilic amido species, as proposed for alkali metals catalysis.

**Scheme 79:**
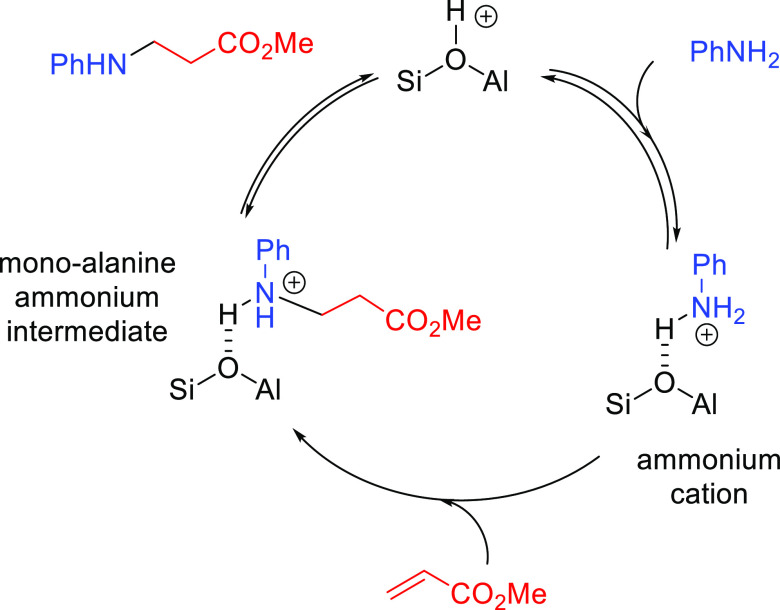
Proposed Reaction Mechanism of the Hydroamination of Methyl Acrylate with Aniline Catalyzed by Zeolites^229^

Sugi and co-workers reported the use of BEA zeolites (β-structured zeolites) as heterogeneous catalysts for the anti-Markovnikov hydroamination of α,β-unsaturated esters with aniline derivatives.^229^ Under the optimized conditions (toluene, 100 °C, 18 h), zeolites with various SiO_2_/Al_2_O_3_ molar ratios (5–25) afforded the hydroamination product with good to moderate yields (around 55–85%), but also the formation of double addition as a byproduct in a 5–10% yield. The authors proposed the amine activation on the acidic sites of the zeolite to form an ammonium cation, followed by the addition of methyl acrylate to give the monoalanine ammonium intermediate which releases the anti-Markovnikov adduct and regenerates the acidic site of catalyst (Scheme 79).

Joseph and co-workers significantly improved the selectivity of the process, obtaining the monoaddition product in high yields (95–97%) using montmorillonite clay K-10.^230^ In a later work, a completely chemoselective anti-Markovnikov hydroamination reaction of activated olefins was achieved by using AlSBA-15 and AlMCM-41 zeolitic catalysts (toluene, 100 °C, 4–10 h), which yielded the monoaddition in yields of 21–95%.^231^ This activity enhancement was attributed to the incorporation of Al atoms on the framework of mesoporous silica, which induced Bronsted and Lewis acidic sites.

Bhanushali and co-workers reported on the use of macroreticular/gel cation exchange resins based as solid heterogeneous catalysts for the anti-Markovnikov hydroamination of vinyl pyridines with aromatic and aliphatic amines.^232^ In this regard, ion exchange resins based on Amberlyst-15/Nafion-NR50 exhibited selective formation of the monoaddition product in moderate to good yields (ethanol, 85–90 °C, 24 h). The recyclability of the catalyst was examined up to four cycles without a significant loss in the activity.

A combination of TiO_2_ nanoparticles stabilized 12-tungstophosphoric acid (TPA) in mesoporous silica (SBA-15) was found to be active for the anti-Markovnikov hydroamination of activated olefins by Vinu and co-workers.^233^ The activity of the catalyst in the hydroamination of ethyl acrylate with aromatic amines was evaluated under various reaction conditions. Under the optimized reaction conditions (toluene, 110 °C, 6 h), conversions up to of 70% were obtained with 100% selectivity for the monoaddition product.

A highly active and selective heterogeneous catalyst with supported Pt was recently used for hydroamination reactions by He and co-workers.^234^ From EPR spectra and in situ FT-IR spectra it has been inferred that the reaction takes places by a cooperation between diverse metal sites (Pt_1_^0^ and Pt_1_^δ+^) in monatomic Pt catalysis. Pt_1_^0^ activates amine to be electrophilic, while Pt_1_^δ+^ activates C=C by π-bonding to make the β-C nucleophilic. The attack of the nucleophilic β-C to the electrophilic amine affords the anti-Markovnikov adduct.^234^

## 6 Conclusions

Despite its inherent difficulty, anti-Markovnikov regioselectivity has been achieved in quite a few hydroamination reactions and in a number of ways, as this review witnesses. Nevertheless, designing a reaction to obtain the anti-Markovnikov adduct is still not a solved issue, mainly because the factors that reverse the usual preference for Markovnikov additions are not well understood. In a direct hydroamination, at some point along the reaction course, the amine lone pair must interact with the π system of the C–C multiple bond in order to form the C–N bond. In a nonsymmetrically substituted unsaturated hydrocarbon, there is a strong preference toward bonding with the more substituted carbon atom of the C–C bond, reminiscent of the Markovnikov rule. However, in systems where barriers for both Markovnikov and anti-Markovnikov additions have been computed, they are usually not very different,^23,69,106,138,172,183^ pointing out that it may be possible to tune the system to favor the anti-Markovnikov pathway. Indeed, in a number of cases, this has been accomplished by changing the substrate.^23,40,122^

A deep knowledge of the factors at work in the step entailing the C–N bond formation is needed to control its regioselectivity, but this requires a good understanding of the reaction mechanism. The analysis of the reaction mechanisms of the hydroaminations performed in this review highlights the disparity of mechanisms by which an N–H bond can add across a C–C multiple bond. This mechanistic diversity is related with the fact that hydroamination catalysts embrace practically almost all of the metal groups of the Periodic Table, from main group metals and early transition metals to late transition metals, lanthanides, and actinides. For the case of alkali, alkaline earth, lanthanide, actinide, and early transition metal catalysts, many of them are attractive because of the affordable price and high abundance. These metal catalysts generally involve having or achieving a M–N bond during the process. Moreover, all these systems share a rather structurally similar transition state for the C–N bond forming step, entailing a four-centered M–N/C–C ring. It seems that this kind of transition state allows an easier reversal of the Markovnikov selectivity than the transition states of other mechanisms, particularly the nucleophilic addition. In such a four-center arrangement, secondary attractive or repulsive interactions between the substituents of the C–C bond and the M–N unit may govern the orientation of the C–N bond formation. Indeed, the experimental preference for the anti-Markovnikov addition in vinyl arenes has been attributed to aryl-directing interactions between the arene π system and the electrophilic metal center.^34^ To form a C–N bond, an empty π*_CC_ orbital must accommodate electron density coming from the amine N lone pair. Accordingly, electronic effects arising from the relative contribution of each carbon atom of the C–C bond to this π*_CC_ orbital and how this orbital and the N lone pair interact may govern the regioselectivity of the addition.^138^

As far as the late transition metals are concerned, their applications to catalyze anti-Markovnikov hydroaminations are still not highly numerous. The complexes are relatively stable in air and tolerant of most of the polar functional groups; therefore, they are convenient to handle and might be applicable to many industrial syntheses in the near future.^33^ The most utilized ones are catalysts based on rhodium and ruthenium metal centers, although other metals, such as Pd, Au, Cu, or Fe, have been also employed. The reason why these systems favor anti-Markovnikov additions are at many times unclear. In these systems there is usually coordination of the C–C π bond to the transition metal, and the attack of the N lone pair must take place at the less substituted carbon, contravening the natural tendency to happen at the more substituted one. Recent theoretical analysis on rhodium- and gold-catalyzed systems pointed out that the origin of the regioselectivity is orbitally driven: anti-Markovnikov attack requires that the π*_CC_ orbital has a significant contribution of a p orbital from the terminal carbon atom. The η^2^ to η^1^ slippage by the alkene displacement of the coordinated olefin localizes the LUMO of the η^2^-C–C complex on the farther carbon atom, enhancing the interaction with the incoming nucleophile. The easiness of this displacement may be at the origin of the regioselectivity.^172,183^

The main difficulties in achieving anti-Markovnikov adducts by direct late transition metal-catalyzed hydroaminations have led to development of several alternative routes named “formal hydroaminations”, which involve a sequence of reactions (such as hydrometalation/electrophilic amination or oxidation/reductive amination) to yield the anti-Markovnikov product. Even though these routes lose the high atom economy of the direct hydroaminations, they have proven to be efficient, mainly for alkene reactions.

In recent years, some new and promising approaches have appeared to perform metal-free or nondirect transition metal-catalyzed anti-Markovnikov hydroaminations. These novel alternatives involve the generation of either *N*-centered or *C*-centered radicals from amine or alkene substrates by a single electron transfer. This fact implies new mechanisms in which the C–N bond formation step is governed by radicals. Biocatalysis involving artificial metalloenzymes, an incipient field with great potential, might also give significant advances in the control of regioselectivity.

Significant practical advances in anti-Markovnikov hydroaminations have been attained in the past few years, but we are still far from “a la carte” design of such processes, as anti-Markovnikov addition reactions are mechanistically very diverse and, therefore, a deeper understanding is still required. In this regard, more combined experimental or spectroscopic and theoretical investigations would be welcome to have a rational understanding of the factors controlling the regioselectivity in hydroamination reactions. We envision that the information collected and analyzed in this review will contribute to that end.
